# Supplementary material for: Unique genetic and risk-factor profiles in clusters of major depressive disorder-related multimorbidity trajectories
Source: Nat Commun. 2024 Aug 21;15:7190. doi: 10.1038/s41467-024-51467-7 (PMC11339304; doi:10.1038/s41467-024-51467-7)
Supplement: Supplementary file 1 — Supplementary Information [file 41467_2024_51467_MOESM1_ESM.pdf]

Supplementary Information

Unique genetic and risk-factor profiles in clusters of major depressive disorder-related multimorbidity trajectories

Andras Gezsi[1 +], Sandra Van der Auwera[2,3 +], Hannu Mäkinen[4], Nora Eszlari[5,6], Gabor Hullam[1,5], Tamas Nagy[1,5,6], Sarah Bonk[2], Rubèn González-Colom[7], Xenia Gonda[5,6,8], Linda Garvert[2], Teemu Paajanen[4], Zsofia Gal[5,6], Kevin Kirchner[2], Andras Millinghoffer[9], Carsten O. Schmidt[10], Bence Bolgar[1], Josep Roca[7], Isaac Cano[7], Mikko Kuokkanen[4,11,12], Peter Antal[1 #], Gabriella Juhasz[5,6 # *]

[+] These authors contributed equally: Andras Gezsi, Sandra Van der Auwera
[#] These authors jointly supervised this work: Peter Antal, Gabriella Juhasz
[*] Corresponding author: Gabriella Juhasz, juhasz.gabriella@semmelweis.hu

[1] Department of Artificial Intelligence and Systems Engineering, Budapest University of Technology and Economics, Műegyetem rkp. 3., H-1111 Budapest, Hungary.
[2] Department of Psychiatry and Psychotherapy, University Medicine Greifswald, 17475 Greifswald, Germany.
[3] German Centre for Neurodegenerative Diseases (DZNE), Site Rostock/Greifswald, 17475 Greifswald, Germany.
[4] Department of Public Health and Welfare, Population Health Unit, Public Health Research Team, Finnish Institute for Health and Welfare, Helsinki, Finland.
[5] Department of Pharmacodynamics, Faculty of Pharmaceutical Sciences, Semmelweis University, Üllői út 26., H-1085 Budapest, Hungary.
[6] NAP3.0-SE Neuropsychopharmacology Research Group, Hungarian Brain Research Program, Semmelweis University, Üllői út 26., H-1085 Budapest, Hungary.
[7] Clínic Barcelona, Fundació de Recerca Clinic Barcelona - Institut d'Investigacions Biomèdiques August Pi i Sunyer (FRCB-IDIBAPS), Universitat de Barcelona, Carrer Rosselló 149-153, Barcelona, 08036 Spain.
[8] Department of Psychiatry and Psychotherapy, Semmelweis University, Üllői út 26., H-1085 Budapest, Hungary.
[9] Abiomics Europe Ltd., Zólyomi út. 23., H-1118 Budapest, Hungary.
[10] Institute for Community Medicine, University Medicine Greifswald, 17475 Greifswald, Germany.
[11] Department of Human Genetics and South Texas Diabetes and Obesity Institute, School of Medicine at University of Texas Rio Grande Valley, Brownsville, TX, United States.
[12] Research Program for Clinical and Molecular Metabolism, Faculty of Medicine, University of Helsinki, Finland.

Content:

- **Ethics statements of participating cohorts** *(p. 4)*

- **Supplementary Figures for results section** *(p. 7)*
    - Figure S1. The distribution of assigned diagnoses per year within the UK Biobank dataset for all conditions and specifically for MDD.
    - Figure S2. The cumulative count of disease diagnoses with increasing age across the three discovery cohorts (UKB, CHSS, THL).

- **Supplementary Figures for results section** *Dynamic Bayesian network analysis reveals seven MDD-related multimorbidity clusters (p. 8)*
    - Figure S3.1. The temporal trajectories of the MDD-associated multimorbidity burden in the discovery cohorts (UKB, THL, CHSS).
    - Figure S3.2. The average onset ages of the consensual diseases according to the seven clusters in the UKB.
    - Figure S4.1-S4.4. Scatter plot of cluster membership variables' coefficients on the onset of all consensual diseases according to UKB versus the other cohorts.
    - Figure S5. Density plot of the age pattern of each cluster using hard cluster assignment for each cohort.
    - Figure S6. Temporal disease patterns of the complete set of psychiatric diseases in the clusters according to UKB (N = 502,504).
    - Figure S7. Kendall's correlation matrix for MDD-related cluster membership probabilities for each cohort.
    - Figures S8.1-S8.7. Distribution of MDD-related cluster membership for Cluster 7 in UKB data.
    - Figure S9. Count distribution of MDD-related cluster membership for Clusters 1-7 in the individual cohorts.

- **Supplementary Figures for results section** *GWAS analysis of MDD-related multimorbidity clusters in the UKB cohort identifies immune system-related genetic profiles (p. 22)*
    - Figure S10.1-S10.7. GWAS results for MDD-related cluster membership in UKB data for Clusters 1-7.
    - Figure S11. Functional overrepresentation analysis of UKB and FinnGen GWAS.
    - Figure S12. Genetic correlation between clusters, MDD case-only clusters, and various depression phenotypes.

- Figure S13. Genetic correlation between clusters, MDD, and the 86 consensual diseases on which the definitions of the clusters are based.
- Figure S14. Comparison of the value of the coefficient in Cox regression of cluster membership on disease onset versus the genetic correlation between the cluster membership and the disease occurrence in the UKB data.
- Figure S15.1-S15.7. Functional modules of the human interactome in Cluster 1-7 that are pleiotropic with MDD.

- **Supplementary Figures for results section** *Non-genetic risk-factor profiles of MDD-related multimorbidity clusters in the UKB cohort (p. 41)*
  - Figure S16. Complex linear regression models involving available non-genetic factors included in the UK Biobank dataset for each cluster.

- **Supplementary Figures for results section** *Validation of MDD-related multimorbidity profiles at the genetic and non-genetic risk-factor levels (p. 42)*
  - Figure S17. Pearson correlation pattern of GWAS derived beta values between MDD-related clusters.
  - Figure S18. Results for polygenic risk score (PRS) analysis in THL.
  - Figure S19.1-S19.7. GWAS results for MDD-related cluster membership in FinnGen data.
  - Figure S20. Scatter plot of gene-level aggregated *p*-values in UKB versus FinnGen.
  - Figure S21. Complex linear regression models involving available non-genetic factors included in the THL data for each cluster.
  - Figure S22. Four measures showing the performance of the clustering method using only a fraction of all consensual disease variables from UKB.
  - Figure S23. Pearson correlation matrix for polygenic risk scores (PRS) in SHIP.
  - Figure S24. Simple linear regression models involving non-genetic risk factors included in the SHIP dataset for each cluster.

- **Supplementary Methods** *(p. 56)*
  - Characterization of the weighted directed MDD-related multimorbidity score *(p. 56)*
  - Determination of the optimal number of clusters *(p. 59)*
  - Transferability of clusters in light of different depression phenotypes in UKB *(p. 76)*

Ethics statements

a. **UK Biobank Ethics statement:** Under the application number *1602* we used data from the UK Biobank (UKB) database resource which includes medical and phenotypic data of recruited participants on the NHS patient registers of people aged 40–69 years (DOI: 10.1371/journal.pone.0075362). Ethical approval was given by the National Research Ethics Service Committee North West – Haydock (11/NW/0382, 21/NW/0157), and all participants gave written informed consent. All procedures were in accordance with the Declaration of Helsinki.

b. **Finngen/THL DF 10 Ethics statement:** Patients and control subjects in FinnGen provided informed consent for biobank research, based on the Finnish Biobank Act. Alternatively, separate research cohorts, collected prior the Finnish Biobank Act came into effect (in September 2013) and start of FinnGen (August 2017), were collected based on study-specific consents and later transferred to the Finnish biobanks after approval by Fimea (Finnish Medicines Agency), the National Supervisory Authority for Welfare and Health. Recruitment protocols followed the biobank protocols approved by Fimea. The Coordinating Ethics Committee of the Hospital District of Helsinki and Uusimaa (HUS) statement number for the FinnGen study is Nr HUS/990/2017.

   The FinnGen study is approved by Finnish Institute for Health and Welfare (permit numbers: THL/2031/6.02.00/2017, THL/1101/5.05.00/2017, THL/341/6.02.00/2018, THL/2222/6.02.00/2018, THL/283/6.02.00/2019, THL/1721/5.05.00/2019 and THL/1524/5.05.00/2020), Digital and population data service agency (permit numbers: VRK43431/2017-3, VRK/6909/2018-3, VRK/4415/2019-3), the Social Insurance Institution (permit numbers: KELA58/522/2017, KELA 131/522/2018, KELA 70/522/2019, KELA 98/522/2019, KELA 134/522/2019, KELA 138/522/2019, KELA 2/522/2020, KELA 16/522/2020), Findata permit numbers THL/2364/14.02/2020, THL/4055/14.06.00/2020, THL/3433/14.06.00/2020, THL/4432/14.06/2020, THL/5189/14.06/2020, THL/5894/14.06.00/2020, THL/6619/14.06.00/2020, THL/209/14.06.00/2021, THL/688/14.06.00/2021, THL/1284/14.06.00/2021,

THL/1965/14.06.00/2021, THL/5546/14.02.00/2020, THL/2658/14.06.00/2021, THL/4235/14.06.00/2021, Statistics Finland (permit numbers: TK-53-1041-17 and TK/143/07.03.00/2020 (earlier TK-53-90-20) TK/1735/07.03.00/2021, TK/3112/07.03.00/2021) and Finnish Registry for Kidney Diseases permission/extract from the meeting minutes on 4thJuly 2019.

The Biobank Access Decisions for FinnGen samples and data utilized in FinnGen Data Freeze 10 include: THL Biobank BB2017_55, BB2017_111, BB2018_19, BB_2018_34, BB_2018_67, BB2018_71, BB2019_7, BB2019_8, BB2019_26, BB2020_1, BB2021_65, Finnish Red Cross Blood Service Biobank 7.12.2017, Helsinki Biobank HUS/359/2017, HUS/248/2020, HUS/150/2022 § 12, §13, §14, §15, §16, §17, §18, and §23, Auria Biobank AB17-5154 and amendment #1 (August 17 2020) and amendments BB_2021-0140, BB_2021-0156 (August 26 2021, Feb 2 2022), BB_2021-0169, BB_2021-0179, BB_2021-0161, AB20-5926 and amendment #1 (April 23 2020)and it´s modification (Sep 22 2021), Biobank Borealis of Northern Finland_2017_1013, 2021_5010, 2021_5018, 2021_5015, 2021_5023, 2021_5017, 2022_6001, Biobank of Eastern Finland 1186/2018 and amendment 22 § /2020, 53§/2021, 13§/2022, 14§/2022, 15§/2022, Finnish Clinical Biobank Tampere MH0004 and amendments (21.02.2020 & 06.10.2020), §8/2021, §9/2022, §10/2022, §12/2022, §20/2022, §21/2022, §22/2022, §23/2022, Central Finland Biobank 1-2017, and Terveystalo Biobank STB 2018001 and amendment 25th Aug 2020, Finnish Hematological Registry and Clinical Biobank decision 18th June 2021, Arctic biobank P0844: ARC_2021_1001.

c. **CHSS Ethics statement:** The Ethics Committee for Human Research at Hospital Clinic de Barcelona approved the study protocol on the 24th of march of 2021 (HCB/2020/1051) in the context of the EU project: ERAPERMED2019-108 - TRAJECTOME. All the data were handled in compliance with the General Data Protection Regulation 2016/679 on data protection and privacy for all individuals within the European Union. The study was conducted in conformity with the Helsinki Declaration (Stronghold Version, Brazil, October 2013) and

in accordance with the protocol and the relevant legal requirements (Biomedical Research Act 14/2007 of 3 July).

d. **SHIP Ethics statement:** The study followed the recommendations of the Declaration of Helsinki. The medical ethics committee of the University of Greifswald approved the study protocol, and oral and written informed consents were obtained from each of the study participants.

Supplementary Figures for results section

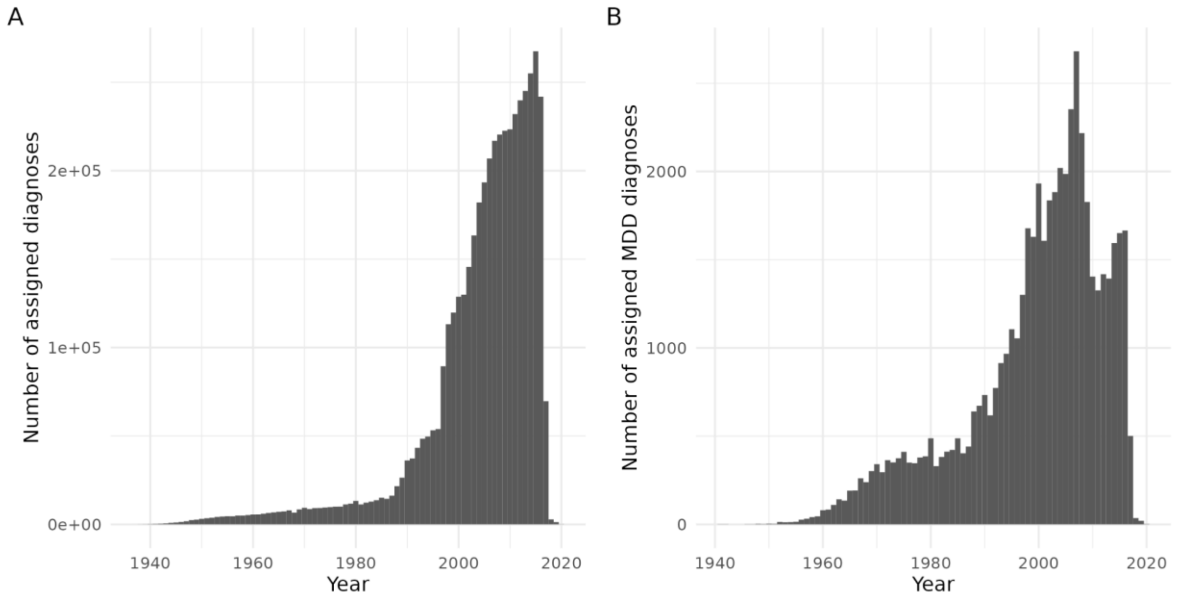

Figure S1. The distribution of assigned diagnoses per year within the UK Biobank dataset for all conditions (A) (N = 502,504) and specifically for MDD (B) (N = 53,473).

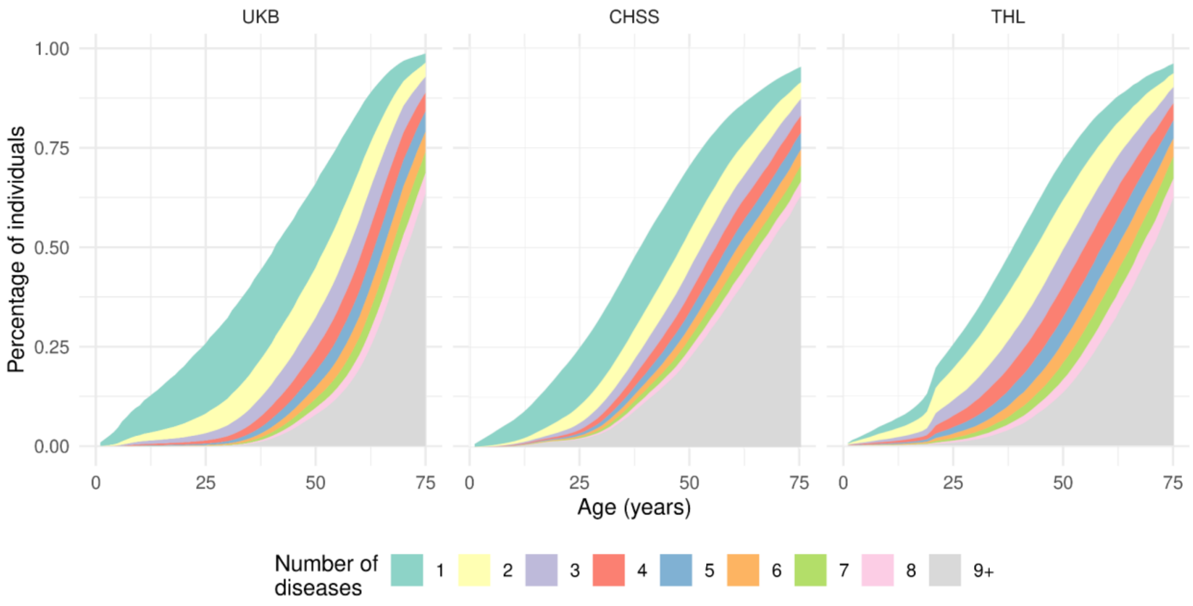

Figure S2. The cumulative count of disease diagnoses with increasing age across the three discovery cohorts (UKB, N = 502,504; CHSS, N = 645,913; THL, N = 41,092).

Supplementary Figures for results section

Dynamic Bayesian network analysis reveals seven MDD-related multimorbidity clusters

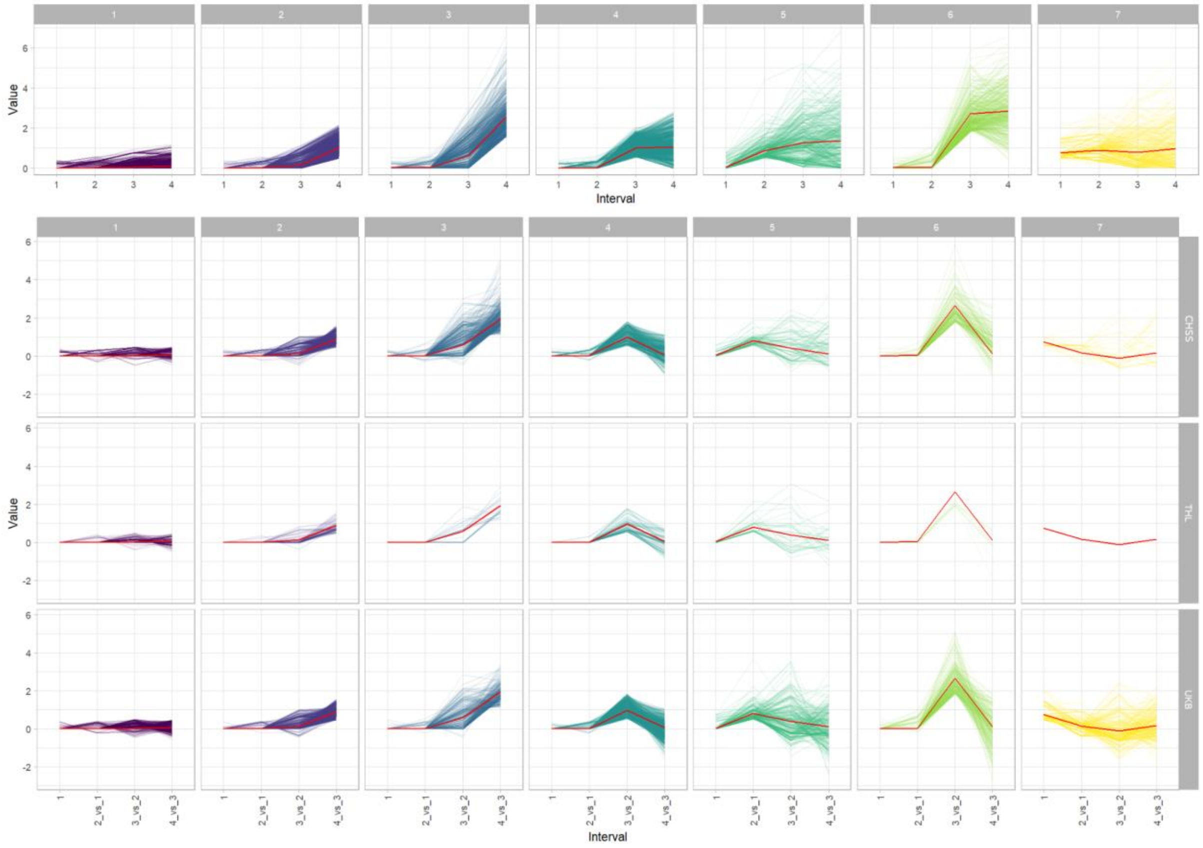

Figure S3.1. The temporal trajectories of the MDD-associated multimorbidity burden in the discovery cohorts (UKB, N = 502,504; CHSS, N = 645,913; THL, N = 41,092). Boxes correspond to clusters. Colored lines denote the trajectories of randomly sampled individuals in each cluster, and the red lines denote the mean trajectories. Top: The trajectories according to all discovery cohorts as a function of the cumulative time intervals. Bottom: The trajectories according to each discovery cohort as a function of the first time interval and the difference between the subsequent consecutive time intervals. Cumulative time intervals 1: 0-20 years, 2: 0-40 years, 3: 0-60 years, 4: 0-70 years.

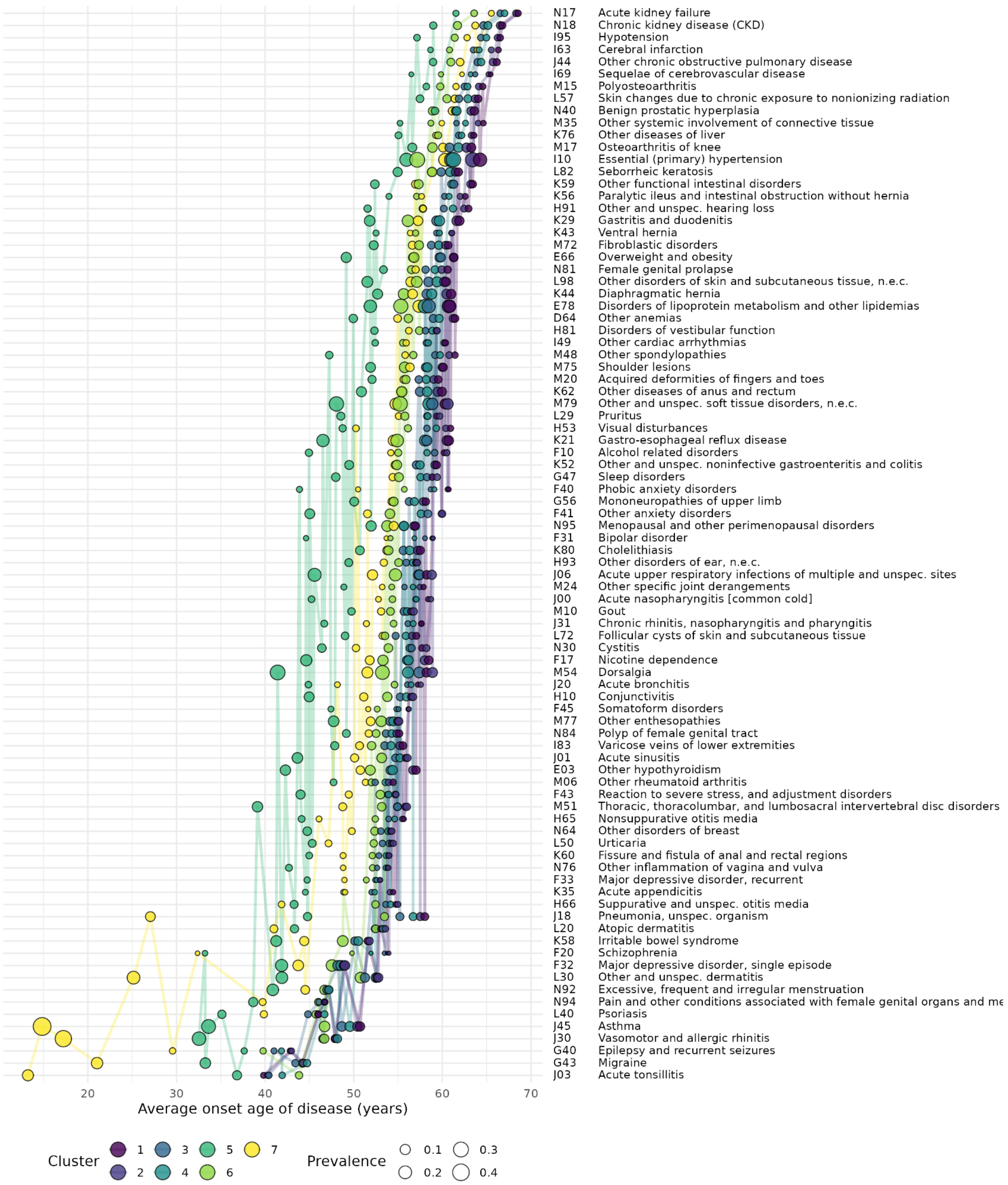

Figure S3.2. The average onset ages of the consensual diseases according to the seven clusters in the UKB (N = 502,504). Average onsets are weighted by the cluster membership probabilities of each participant. The nodes' color indicates the cluster, and the node size is proportional to the observed prevalence of the disease in the cluster.

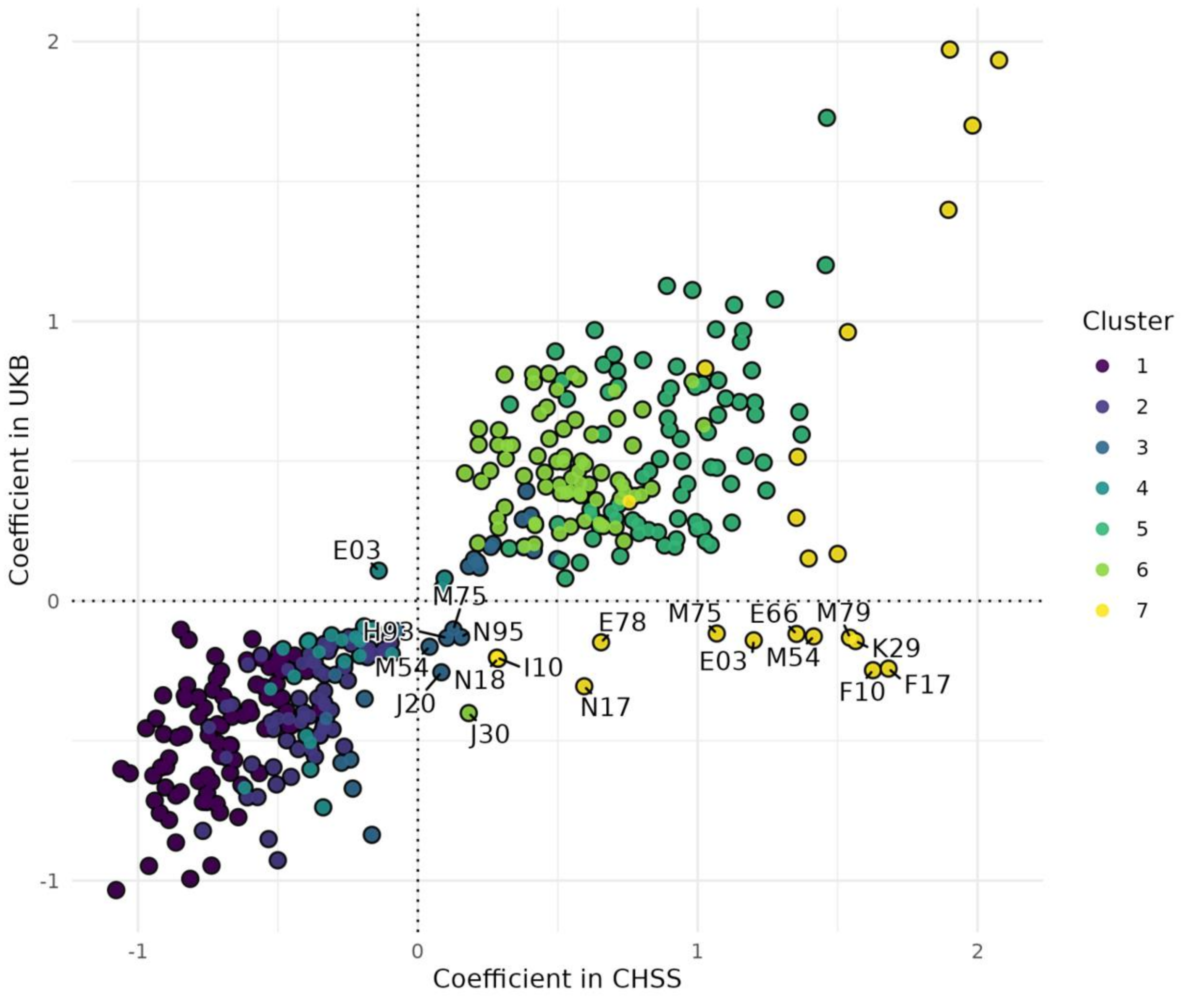

Figure S4.1. Scatter plot of cluster membership variables' coefficients on the onset of all consensual diseases according to CHSS (N = 645,913) versus UKB (N = 502,504). We used weighted Cox regression to determine the disease outcomes in the various clusters (i.e., the hazard ratio of cluster membership regarding disease occurrence) independently for each cohort. Specifically, for each cluster, we constructed Cox proportional hazard models, where the independent variable for a specific individual was a dummy variable created in the following way. We counted each participant twice, summing to a weight of 1. First, we set the value of the dummy variable to 1 and weighted this sample by the posterior probability of cluster membership. Next, we set the value of the dummy variable to 0 and used a weight for this sample equal to 1 minus the probability of cluster membership. The covariates were sex, household income (if available), and the normalized birth year (in the case of the UKB cohort). The dependent variable was disease onset. Participants were right censored for a given target disease at their age if the disease was not diagnosed. We calculated separate models for each cross-cohort disease. P-values of the cluster membership variables were adjusted separately for each cohort using the Benjamini–Hochberg method. Each point represents a disease according to a given cluster (indicated by the point's color). Only those diseases are shown which are statistically significant (adjusted p-value < 0.05) in both cohorts. Diseases for which the sign of the coefficients differ are indicated with labels.

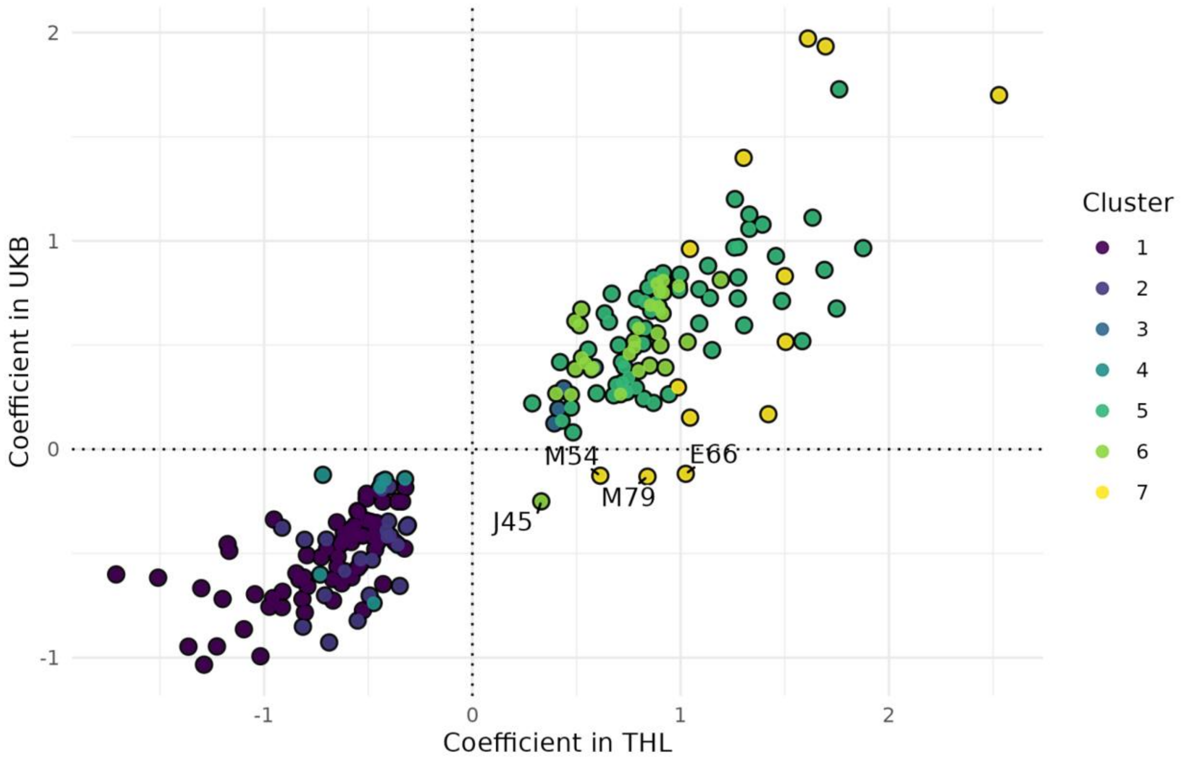

Figure S4.2. Scatter plot of cluster membership variables' coefficients on the onset of all consensual diseases according to THL (N = 41,092) versus UKB (N = 502,504). Each point represents a disease according to a given cluster (indicated by the point's color). Only those diseases are shown which are statistically significant in both cohorts. Diseases for which the sign of the coefficients differ are indicated with labels. See Figure S4.1 for details of the analysis.

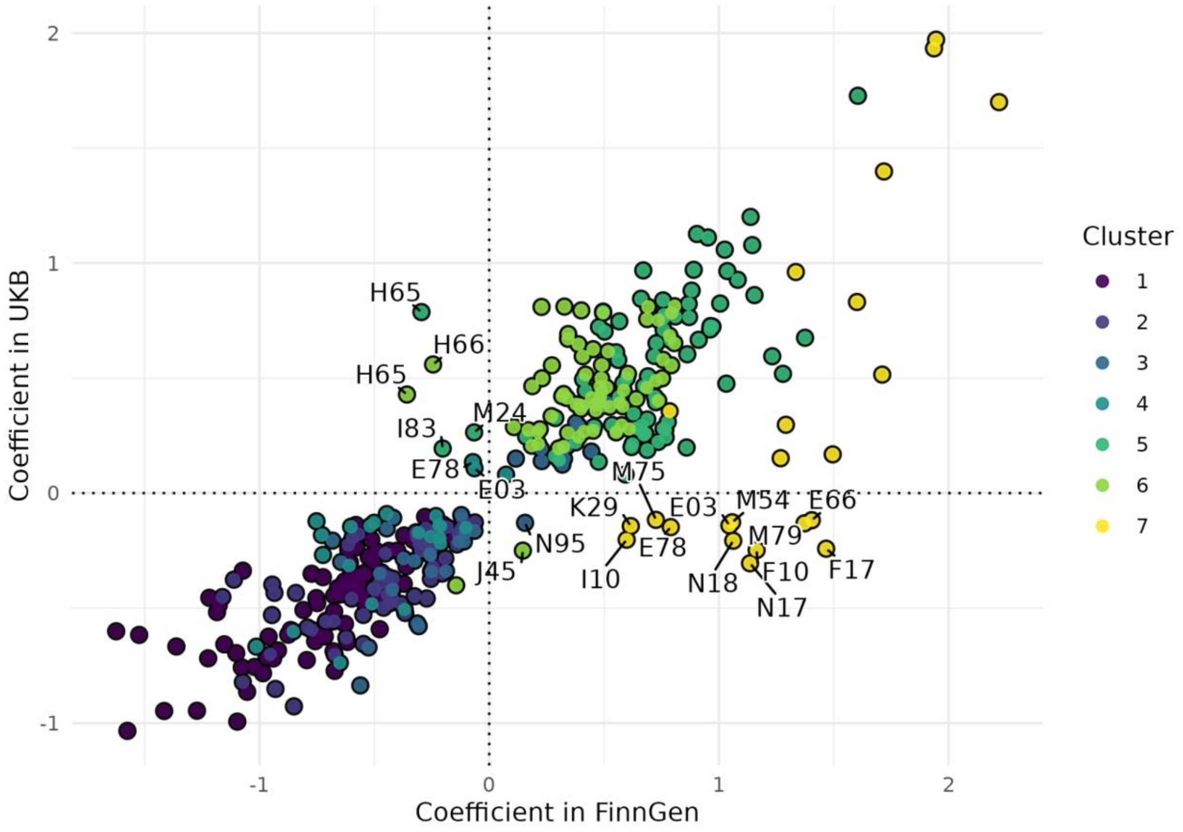

Figure S4.3. Scatter plot of cluster membership variables' coefficients on the onset of all consensual diseases according to FinnGen (N = 385,640) versus UKB (N = 502,504). Each point represents a disease according to a given cluster (indicated by the point's color). Only those diseases are shown which are statistically significant in both cohorts. Diseases for which the sign of the coefficients differ are indicated with labels. See Figure S4.1 for details of the analysis.

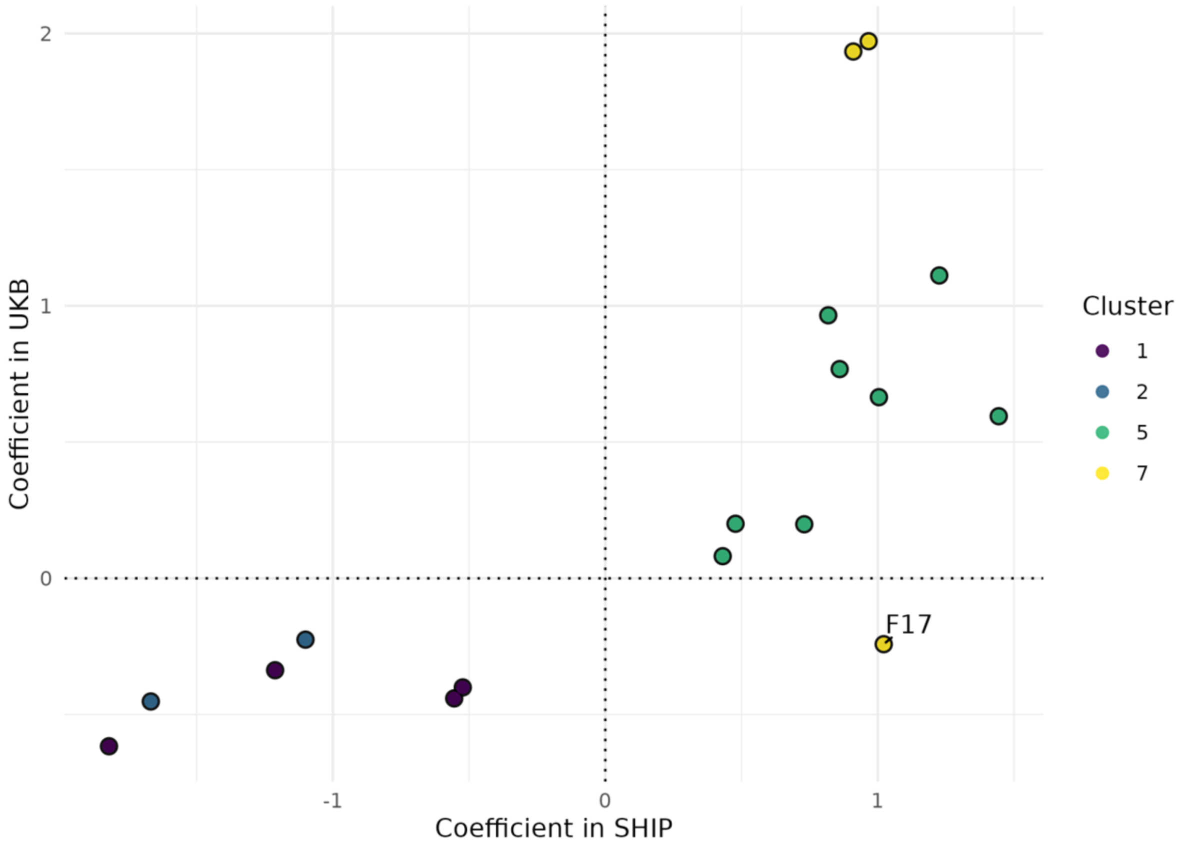

Figure S4.4. Scatter plot of cluster membership variables' coefficients on the onset of all consensual diseases according to SHIP (N = 1449) versus UKB (N = 502,504). Each point represents a disease according to a given cluster (indicated by the point's color). Only those diseases are shown which are statistically significant in both cohorts. The disease for which the sign of the coefficient differs is indicated with a label (F17). See Figure S4.1 for details of the analysis.

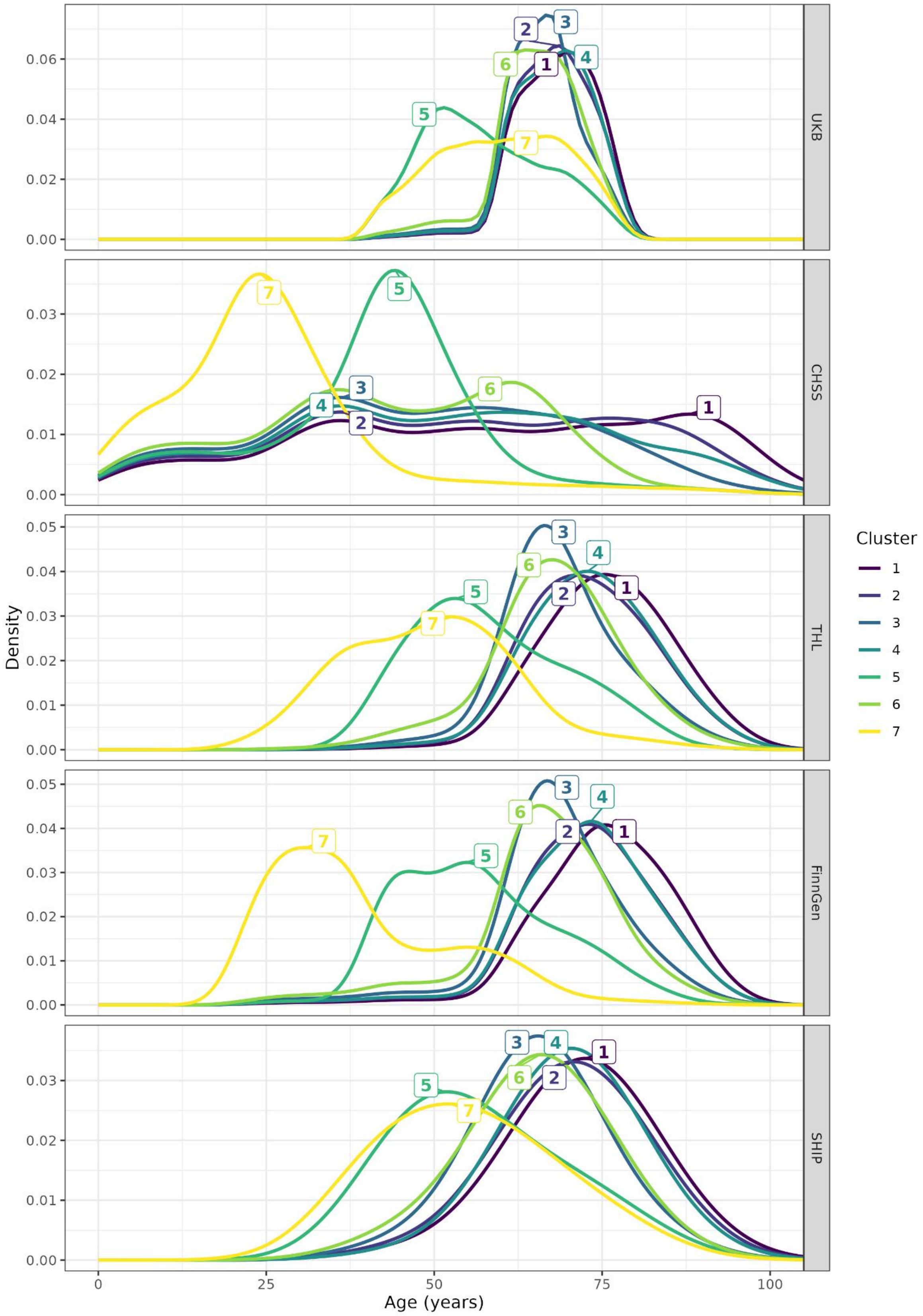

Figure S5. Density plot of the age pattern of each cluster in each cohort. Participants are weighted by the posterior probability of belonging to a cluster. Each colored and labeled line corresponds to a certain cluster in the cohort. Participants were excluded if both being under 60 years and having a maximum posterior probability < 0.25 to any of the clusters. UKB N = 364,008; CHSS N = 645,913; THL N = 23,786; FinnGen N = 277,252; SHIP N = 1126.

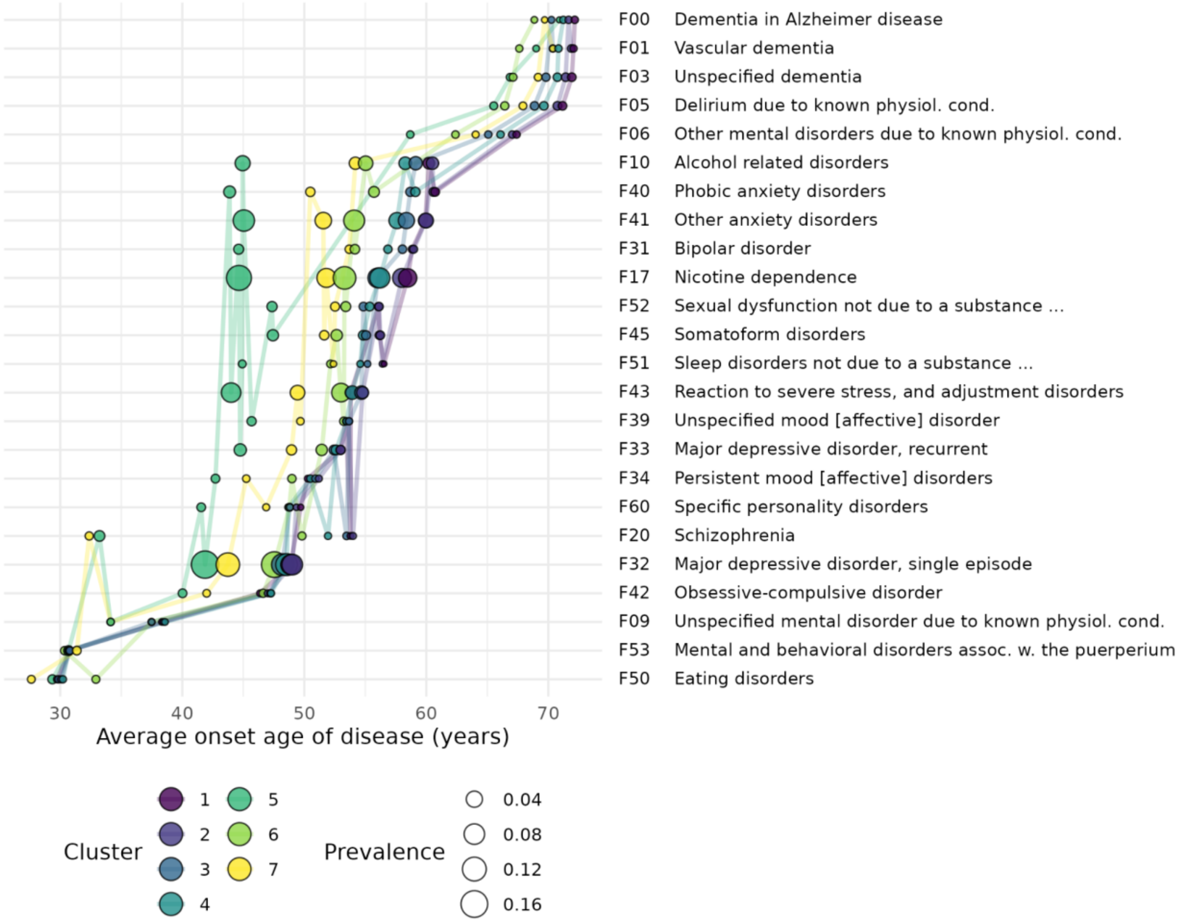

Figure S6. Temporal disease patterns of the complete set of psychiatric diseases in the clusters according to UKB (N = 502,504). The average onset ages of the psychiatric diseases (Chapter V. of ICD-10: F00-F99) per line according to the seven clusters in the UKB cohort. The node color indicates the clusters and the node size is proportional to the observed prevalence of the disease in the cluster.

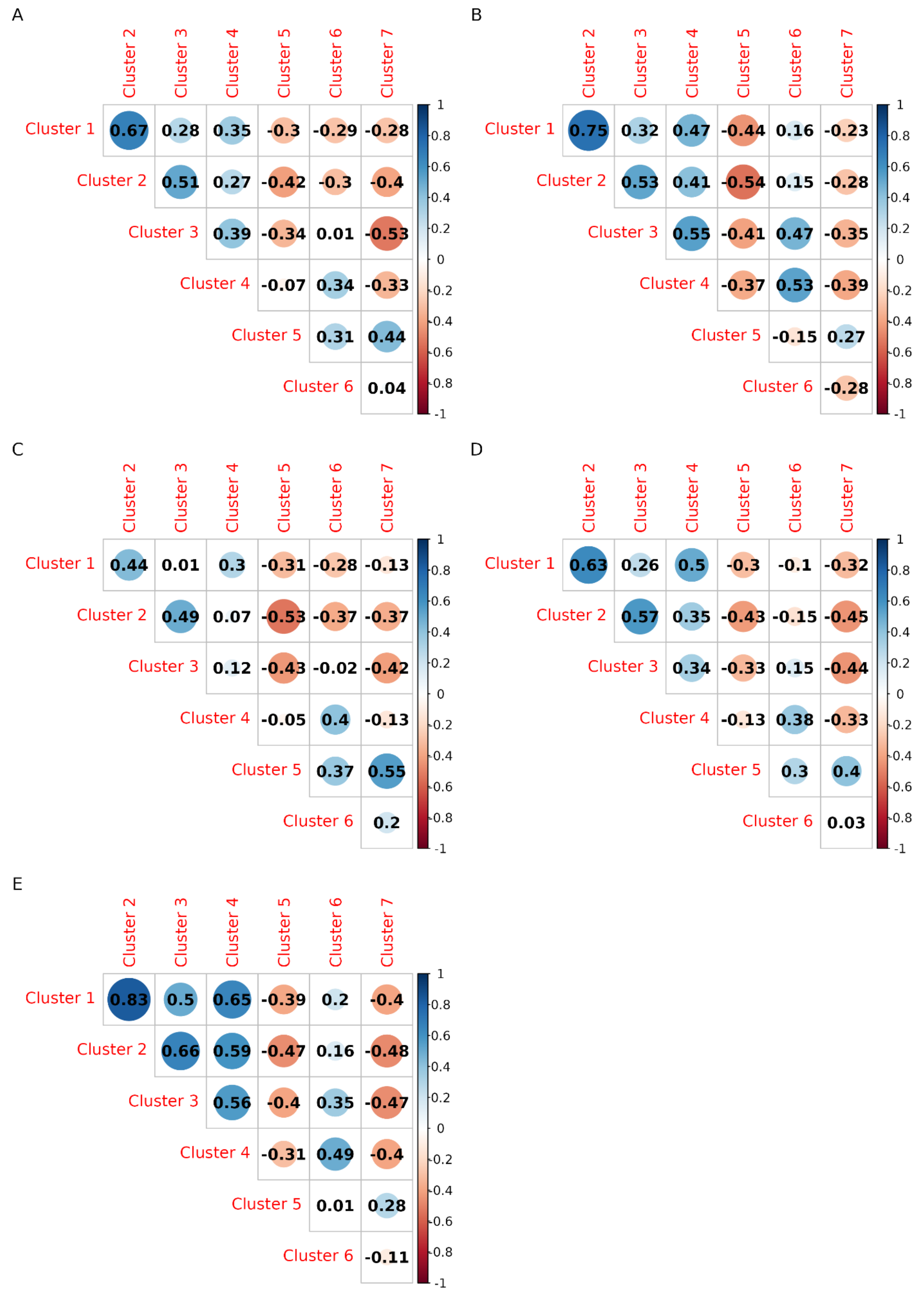

Figure S7. Kendall's correlation matrix for cluster membership probabilities for each cohort. Kendall correlation coefficients were computed on filtered samples; participants were excluded if both being under 60 years and having a maximum posterior probability < 0.25 to any of the clusters. The colour of the dots indicates the value of the correlation. (A) UKB (N = 364,008); (B) CHSS (N = 645,913); (C) THL (N = 23,786); (D) FinnGen (N = 277,252); (E) SHIP (N = 1126).

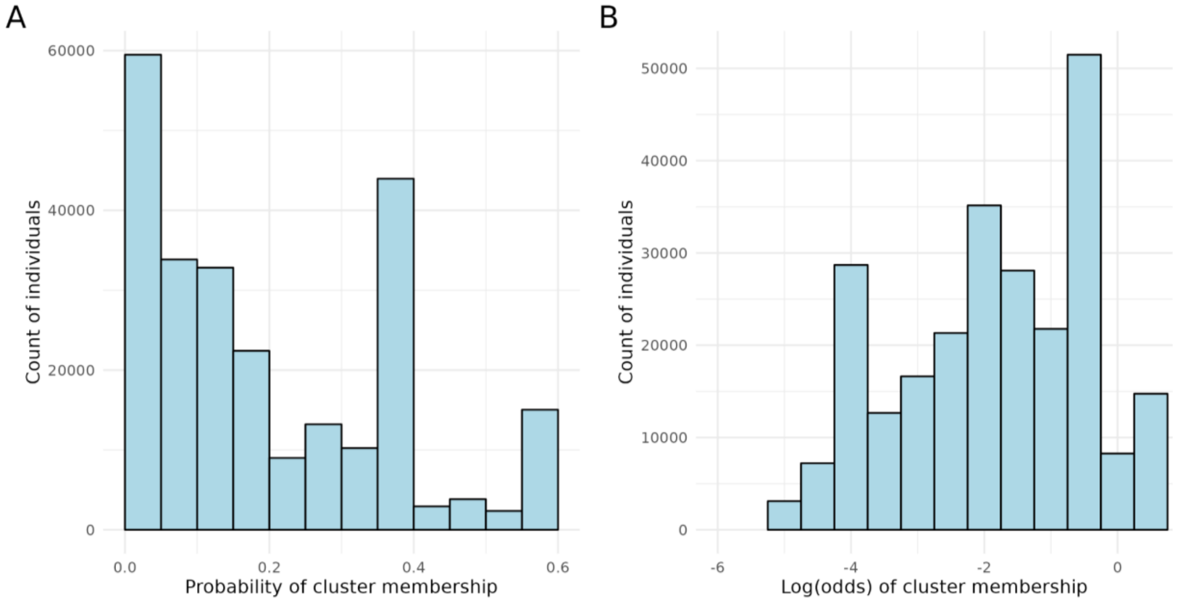

Figure S8.1. Distribution of MDD-related cluster membership for Cluster 1 in UKB data (N = 249,167). Raw probability (A) and posterior log-odds (B).

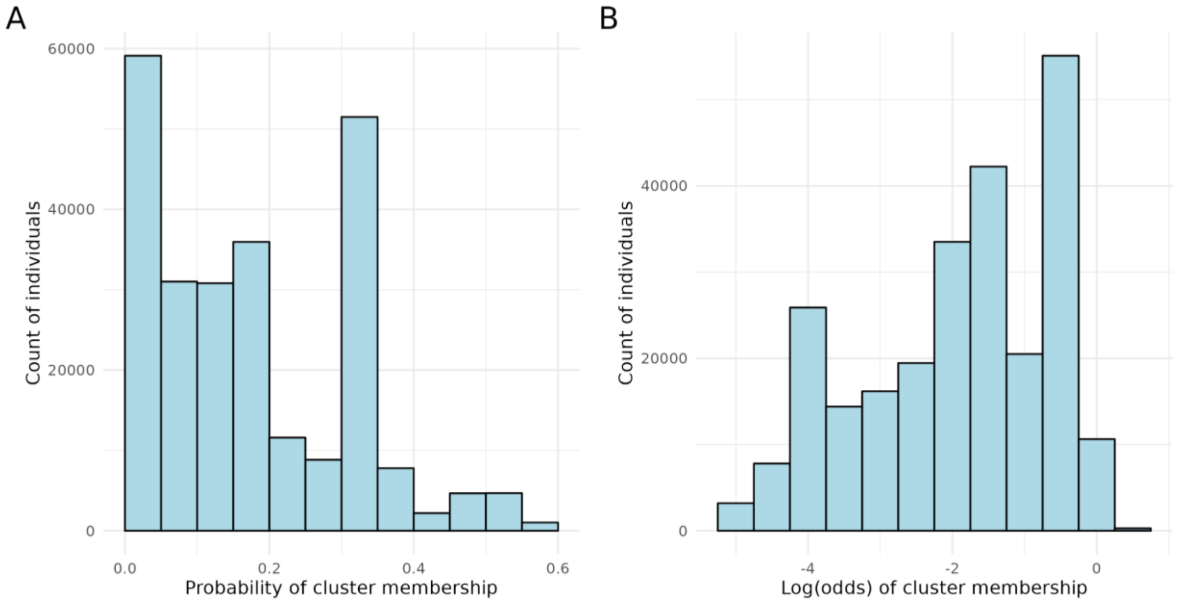

Figure S8.2. Distribution of MDD-related cluster membership for Cluster 2 in UKB data (N = 249,167). Raw probability (A) and posterior log-odds (B).

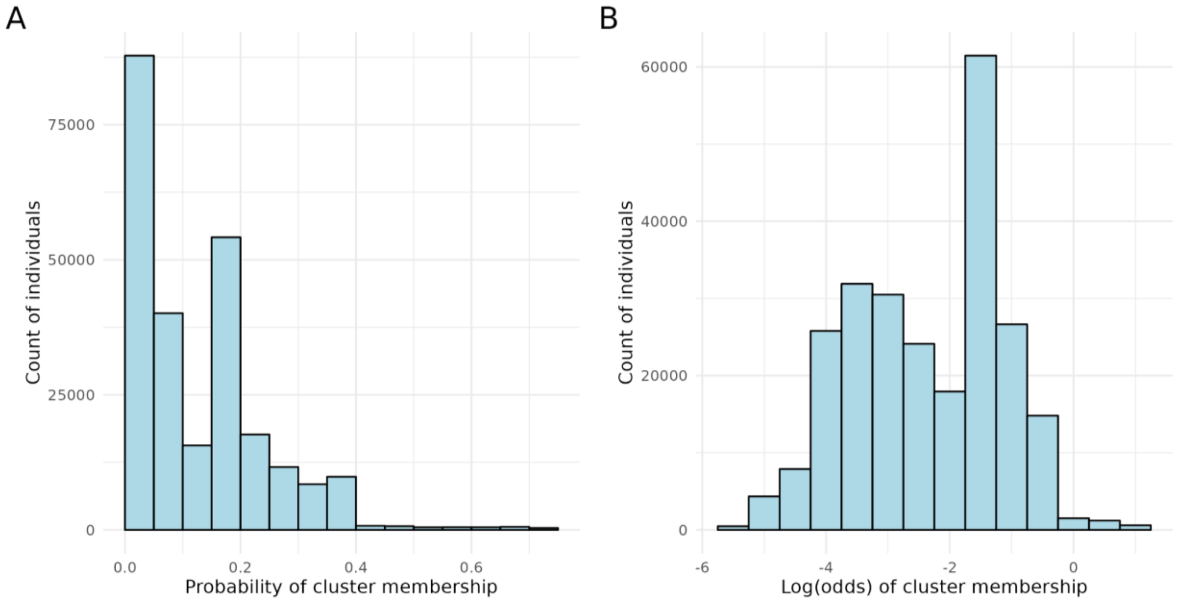

Figure S8.3. Distribution of MDD-related cluster membership for Cluster 3 in UKB data (N = 249,167). Raw probability (A) and posterior log-odds (B).

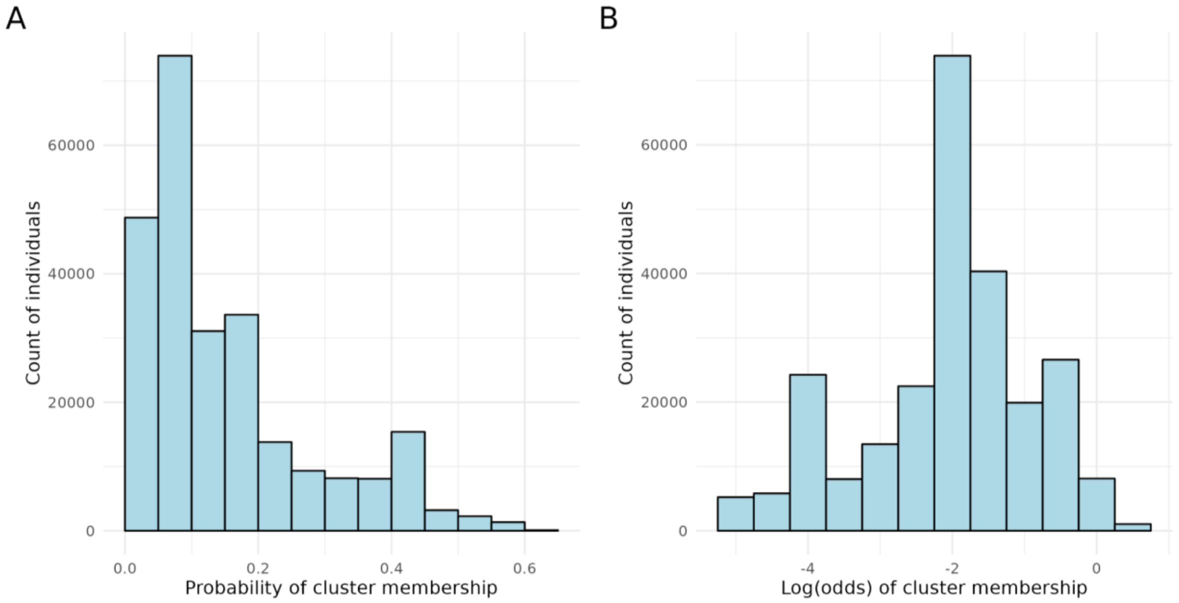

Figure S8.4. Distribution of MDD-related cluster membership for Cluster 4 in UKB data (N = 249,167). Raw probability (A) and posterior log-odds (B).

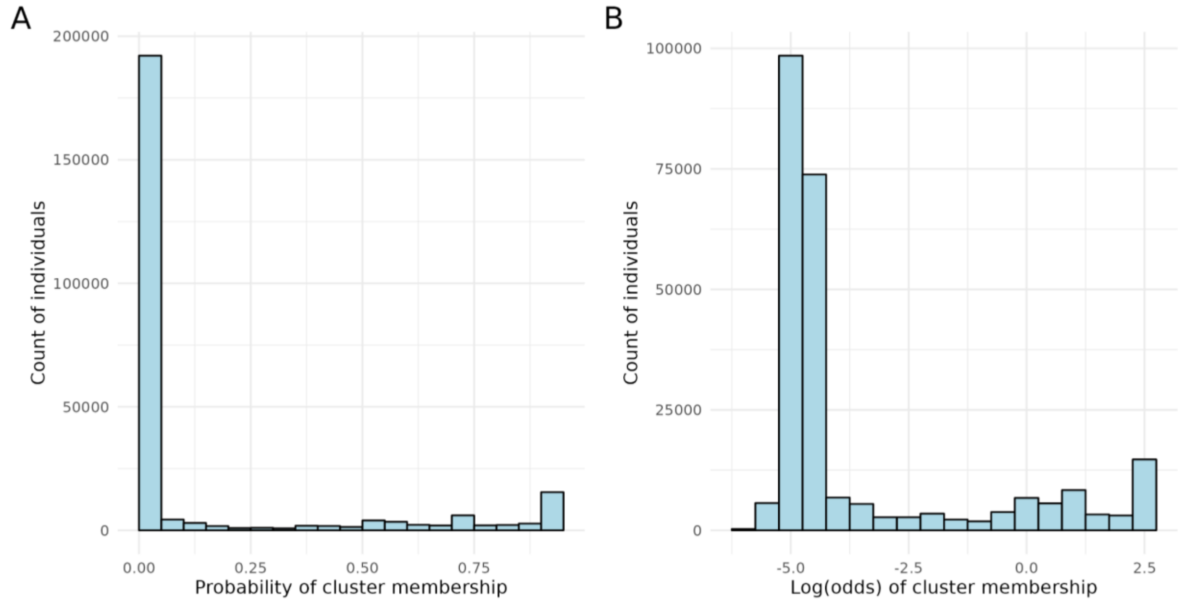

Figure S8.5. Distribution of MDD-related cluster membership for Cluster 5 in UKB data (N = 249,167). Raw probability (A) and posterior log-odds (B).

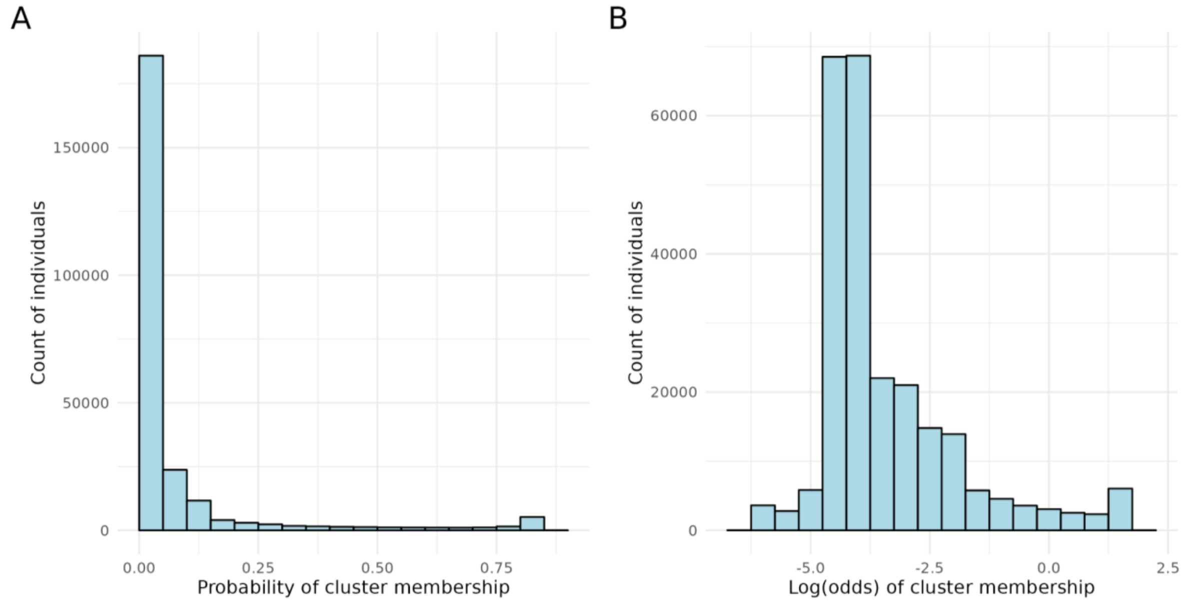

Figure S8.6. Distribution of MDD-related cluster membership for Cluster 6 in UKB data (N = 249,167). Raw probability (A) and posterior log-odds (B).

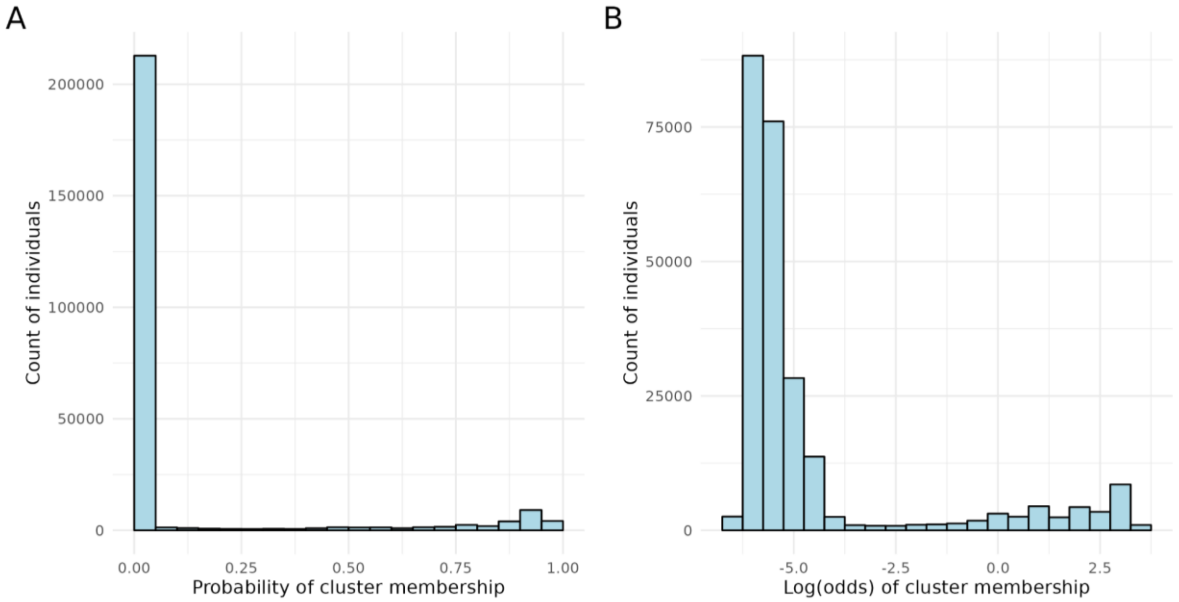

Figure S8.7. Distribution of MDD-related cluster membership for Cluster 7 in UKB data (N = 249,167). Raw probability (A) and posterior log-odds (B).

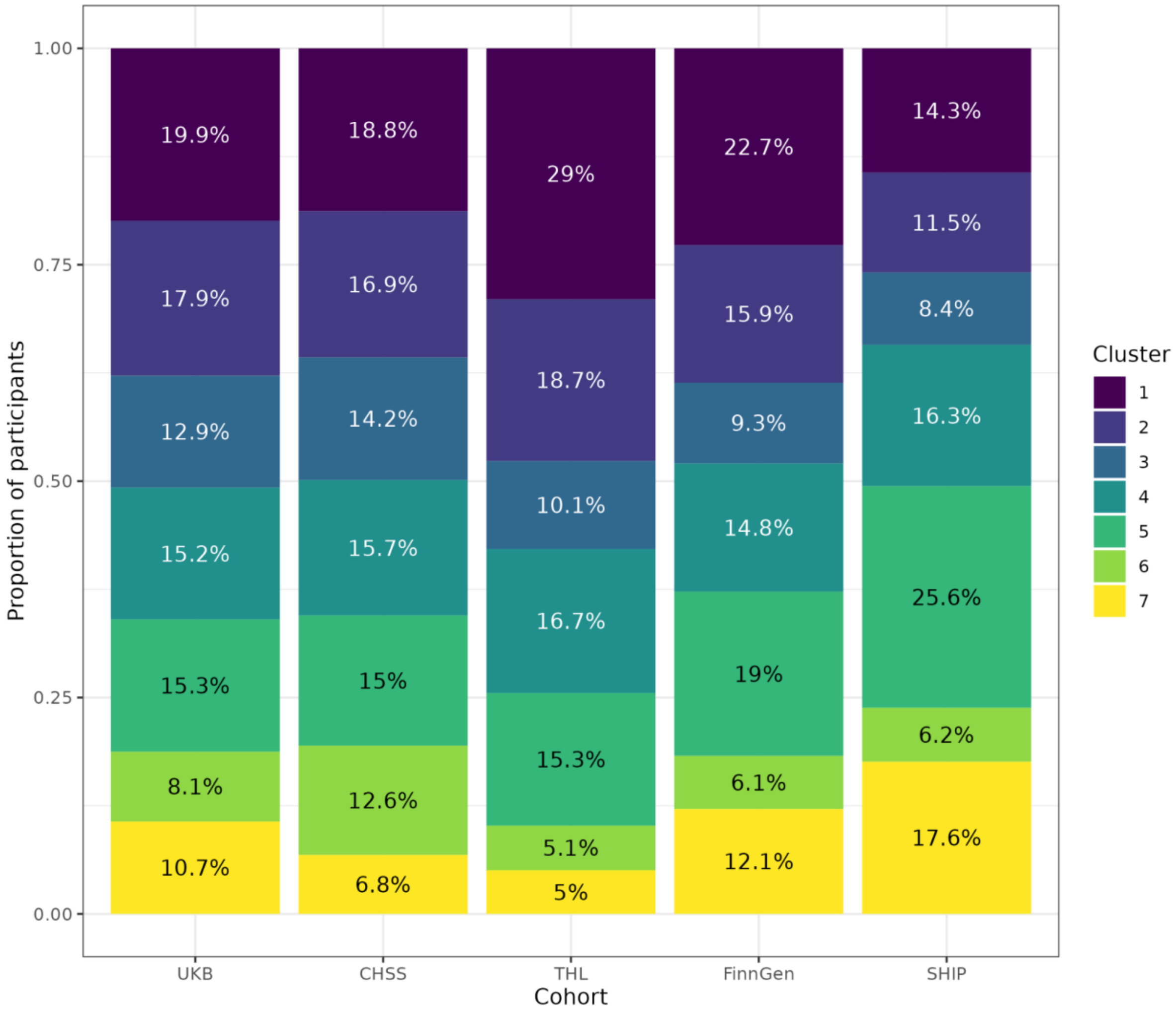

Figure S9. Count distribution of MDD-related cluster membership for Clusters 1-7 in the individual cohorts. Coloured bars show the expected proportion of participants belonging to each cluster (indicated by the colour). Each individual is weighted by their cluster membership probability; i.e., to compute the expected number of participants in a cluster, we summed each participant's corresponding cluster membership probability. UKB N = 502,504; CHSS N = 645,913; THL N = 41,092; FinnGen N = 385,640; SHIP N = 1449

Supplementary Figures for results section

GWAS analysis of MDD-related multimorbidity clusters in the UKB cohort identifies immune system-related genetic profiles

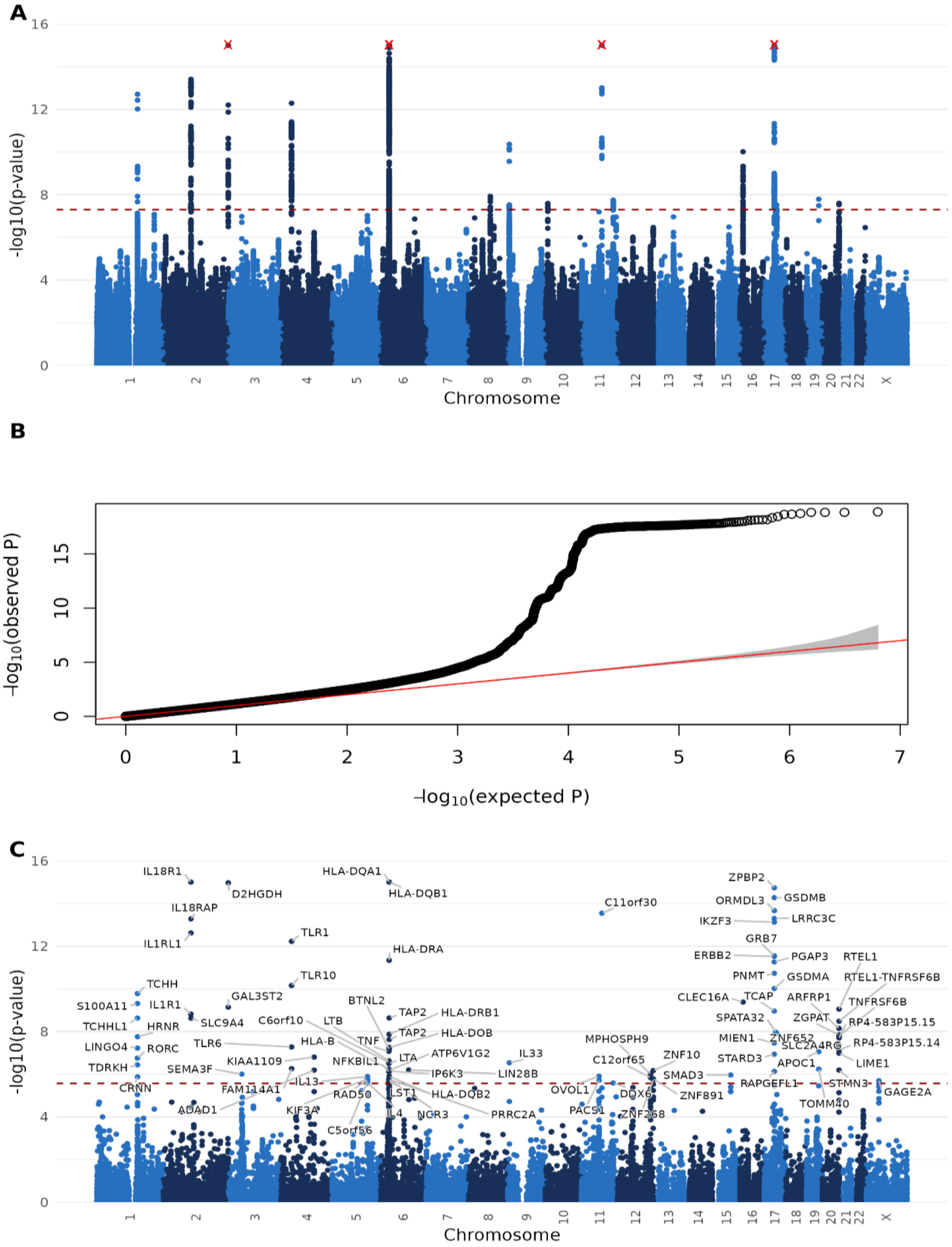

Figure S10.1. GWAS results for MDD-related Cluster 1 membership in UKB data (N = 249,167). (A) SNP-based genome-wide Manhattan plot. Association analyses were performed using linear regression to test the association between each SNP and the posterior log odds of cluster membership, controlling for age, sex, the first ten genetic principal components, and the genotyping array. In the plot, nominal p-values are displayed. Results capped at 10^{-15} are indicated with red crosses. The significance threshold (5×10^{-8}) is depicted with a dashed dark red line. (B) QQ-plot. (C) Gene-based genome-wide Manhattan plot. MAGMA gene-level analysis was performed to identify putative significant genes using a SNPwise-multi model, defining the SNP set of each gene with a ± 10 kb window. In the plot, nominal p-values are displayed. The statistically significant genes are indicated with labels. The significance threshold (2.7×10^{-6}) is depicted with a dashed dark red line.

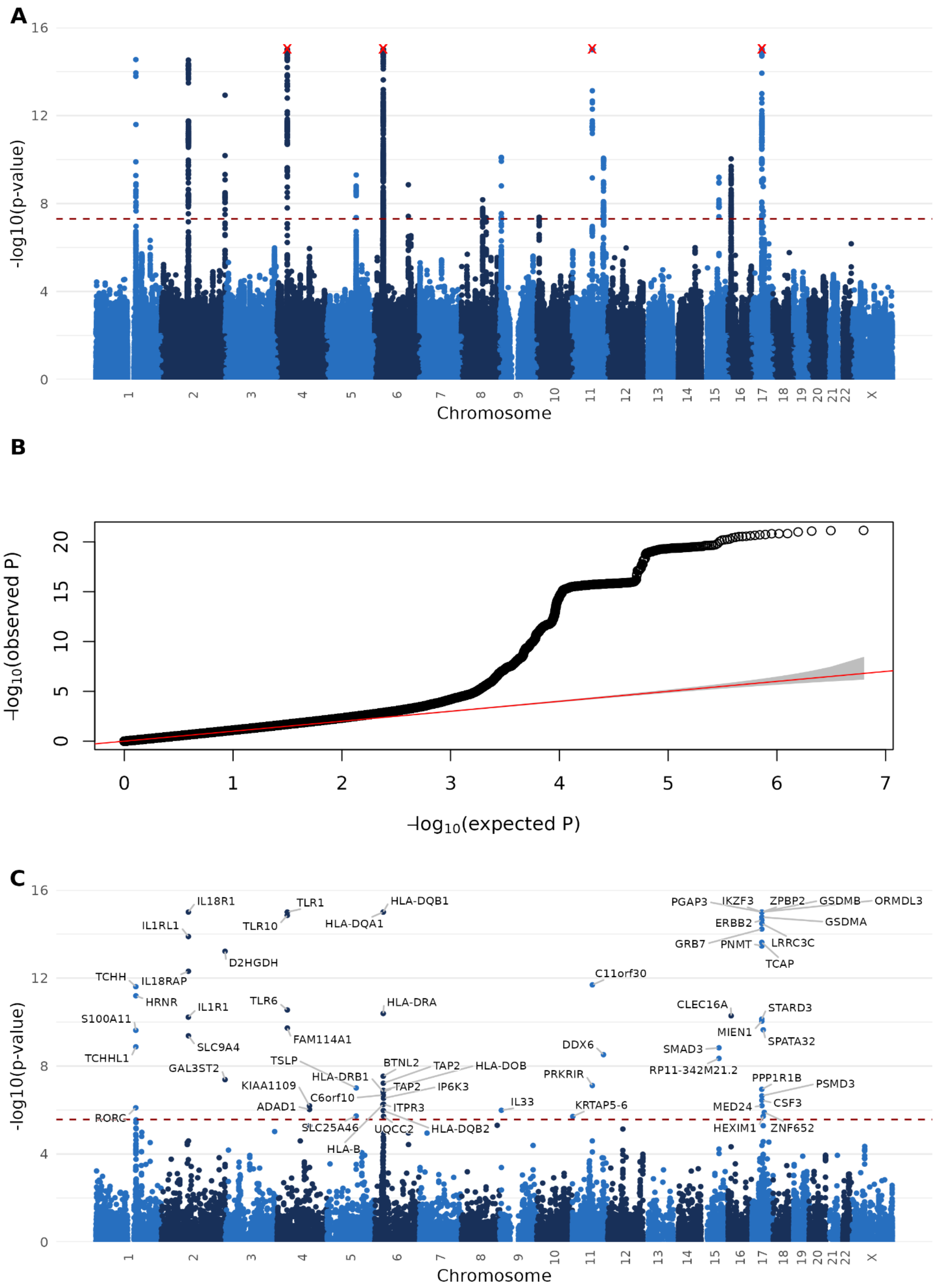

Figure S10.2. GWAS results for MDD-related Cluster 2 membership in UKB data (N = 249,167). (A) SNP-based genome-wide Manhattan plot. Association analyses were performed using linear regression to test the association between each SNP and the posterior log odds of cluster membership, controlling for age, sex, the first ten genetic principal components, and the genotyping array. In the plot, nominal p-values are displayed. Results capped at 10^{-15} are indicated with red crosses. The significance threshold (5×10^{-8}) is depicted with a dashed dark red line. (B) QQ-plot. (C) Gene-based genome-wide Manhattan plot. MAGMA gene-level analysis was performed to identify putative significant genes using a SNPwise-multi model, defining the SNP set of each gene with a ± 10 kb window. In the plot, nominal p-values are displayed. The statistically significant genes are indicated with labels. The significance threshold (2.7×10^{-6}) is depicted with a dashed dark red line.

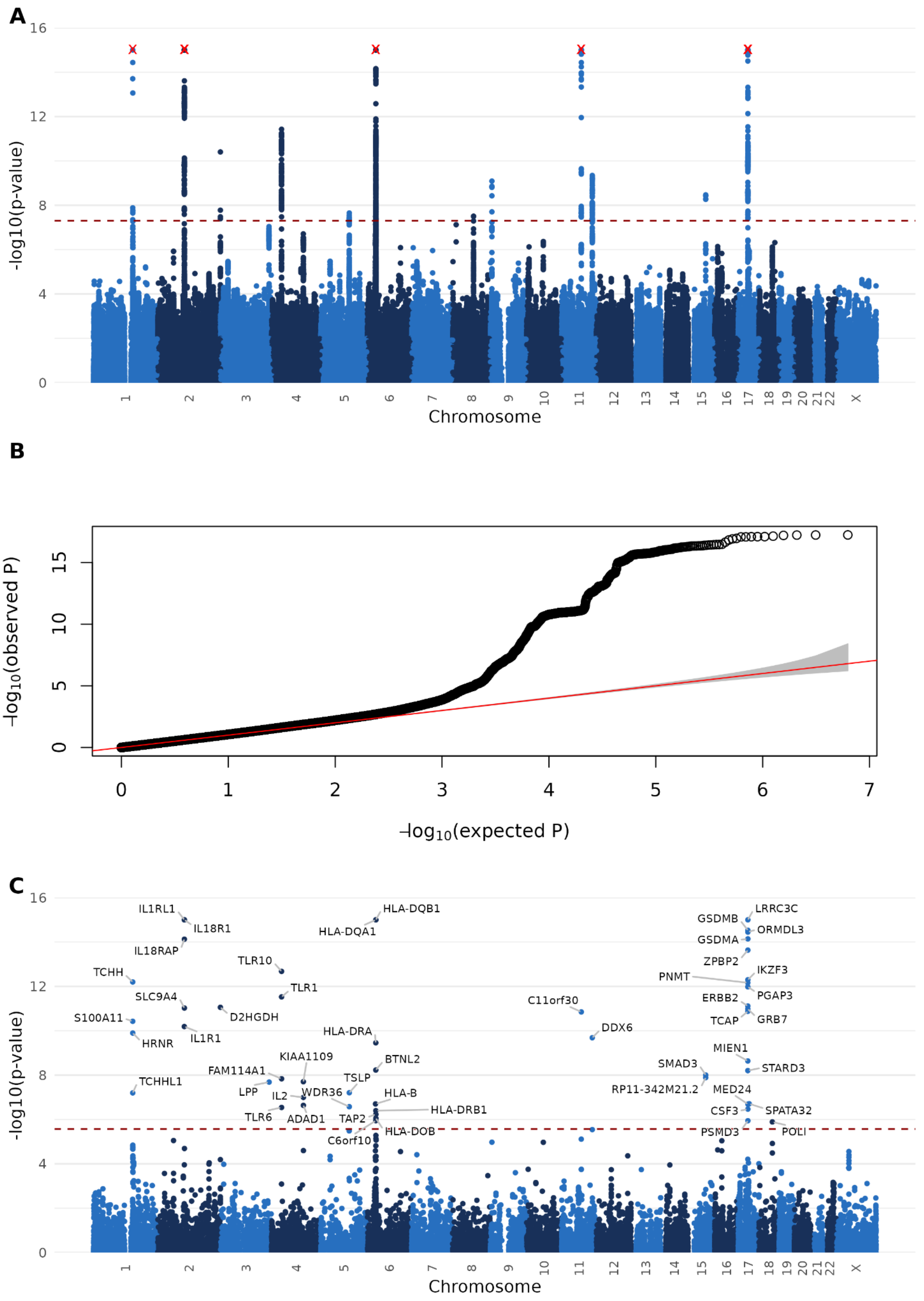

Figure S10.3. GWAS results for MDD-related Cluster 3 membership in UKB data (N = 249,167). (A) SNP-based genome-wide Manhattan plot. Association analyses were performed using linear regression to test the association between each SNP and the posterior log odds of cluster membership, controlling for age, sex, the first ten genetic principal components, and the genotyping array. In the plot, nominal p-values are displayed. Results capped at 10^{-15} are indicated with red crosses. The significance threshold (5×10^{-8}) is depicted with a dashed dark red line. (B) QQ-plot. (C) Gene-based genome-wide Manhattan plot. MAGMA gene-level analysis was performed to identify putative significant genes using a SNPwise-multi model, defining the SNP set of each gene with a ± 10 kb window. In the plot, nominal p-values are displayed. The statistically significant genes are indicated with labels. The significance threshold (2.7×10^{-6}) is depicted with a dashed dark red line.

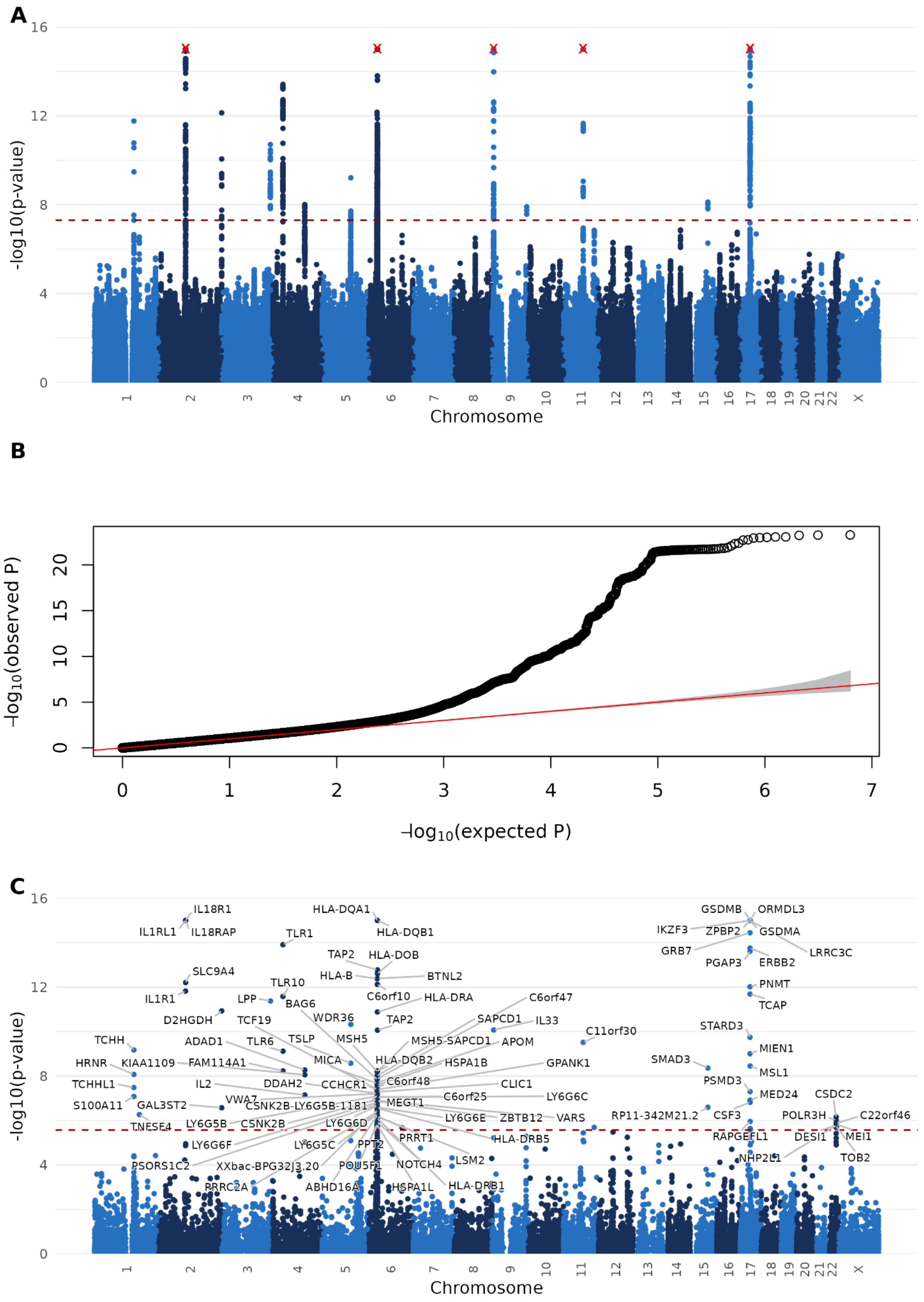

Figure S10.4. GWAS results for MDD-related Cluster 4 membership in UKB data (N = 249,167). (A) SNP-based genome-wide Manhattan plot. Association analyses were performed using linear regression to test the association between each SNP and the posterior log odds of cluster membership, controlling for age, sex, the first ten genetic principal components, and the genotyping array. In the plot, nominal p-values are displayed. Results capped at 10^{-15} are indicated with red crosses. The significance threshold (5×10^{-8}) is depicted with a dashed dark red line. (B) QQ-plot. (C) Gene-based genome-wide Manhattan plot. MAGMA gene-level analysis was performed to identify putative significant genes using a SNPwise-multi model, defining the SNP set of each gene with a ± 10 kb window. In the plot, nominal p-values are displayed. The statistically significant genes are indicated with labels. The significance threshold (2.7×10^{-6}) is depicted with a dashed dark red line.

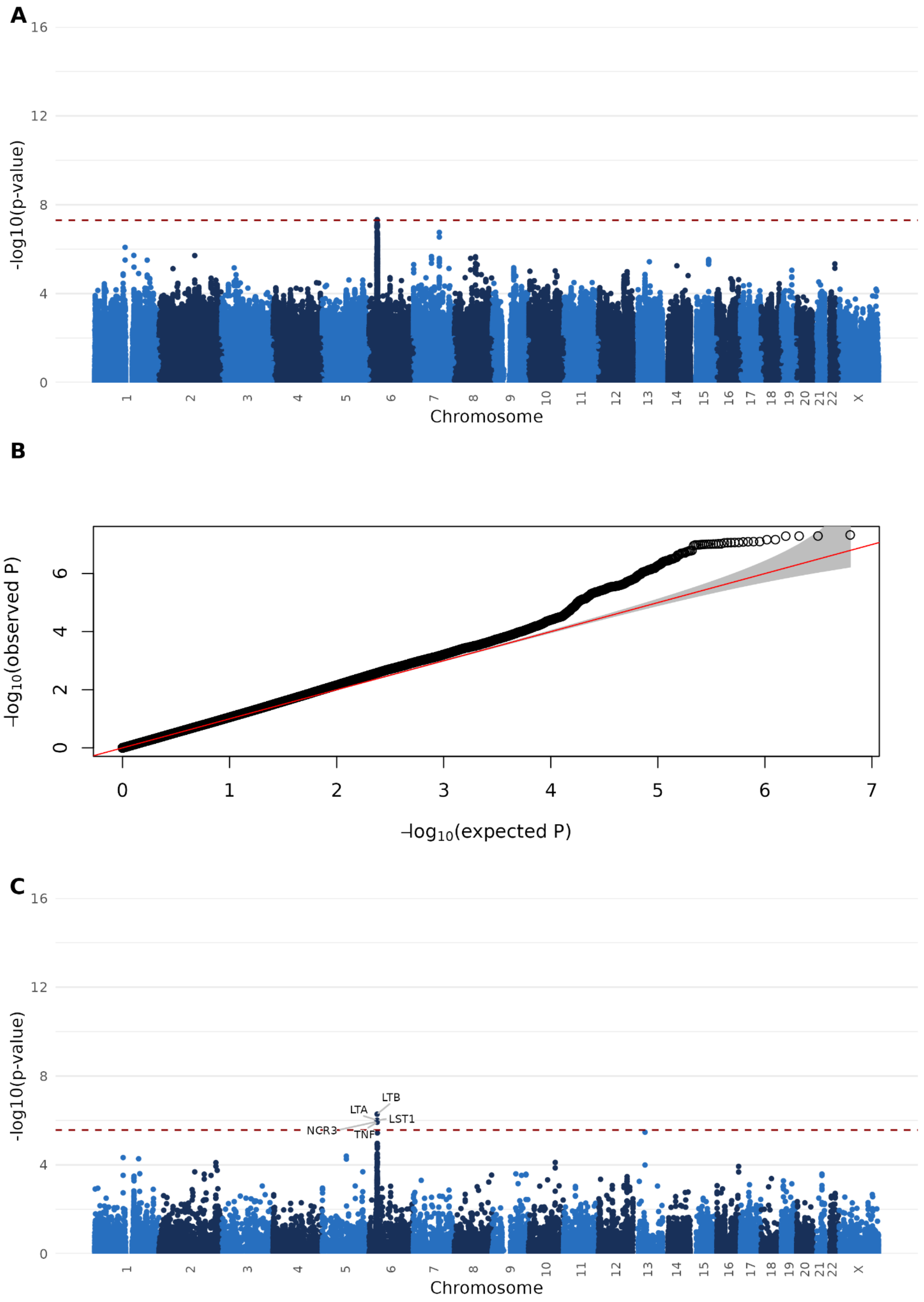

Figure S10.5. GWAS results for MDD-related Cluster 5 membership in UKB data (N = 249,167). (A) SNP-based genome-wide Manhattan plot. Association analyses were performed using linear regression to test the association between each SNP and the posterior log odds of cluster membership, controlling for age, sex, the first ten genetic principal components, and the genotyping array. In the plot, nominal p-values are displayed. The significance threshold (5×10^{-8}) is depicted with a dashed dark red line. (B) QQ-plot. (C) Gene-based genome-wide Manhattan plot. MAGMA gene-level analysis was performed to identify putative significant genes using a SNPwise-multi model, defining the SNP set of each gene with a ± 10 kb window. In the plot, nominal p-values are displayed. The statistically significant genes are indicated with labels. The significance threshold (2.7×10^{-6}) is depicted with a dashed dark red line.

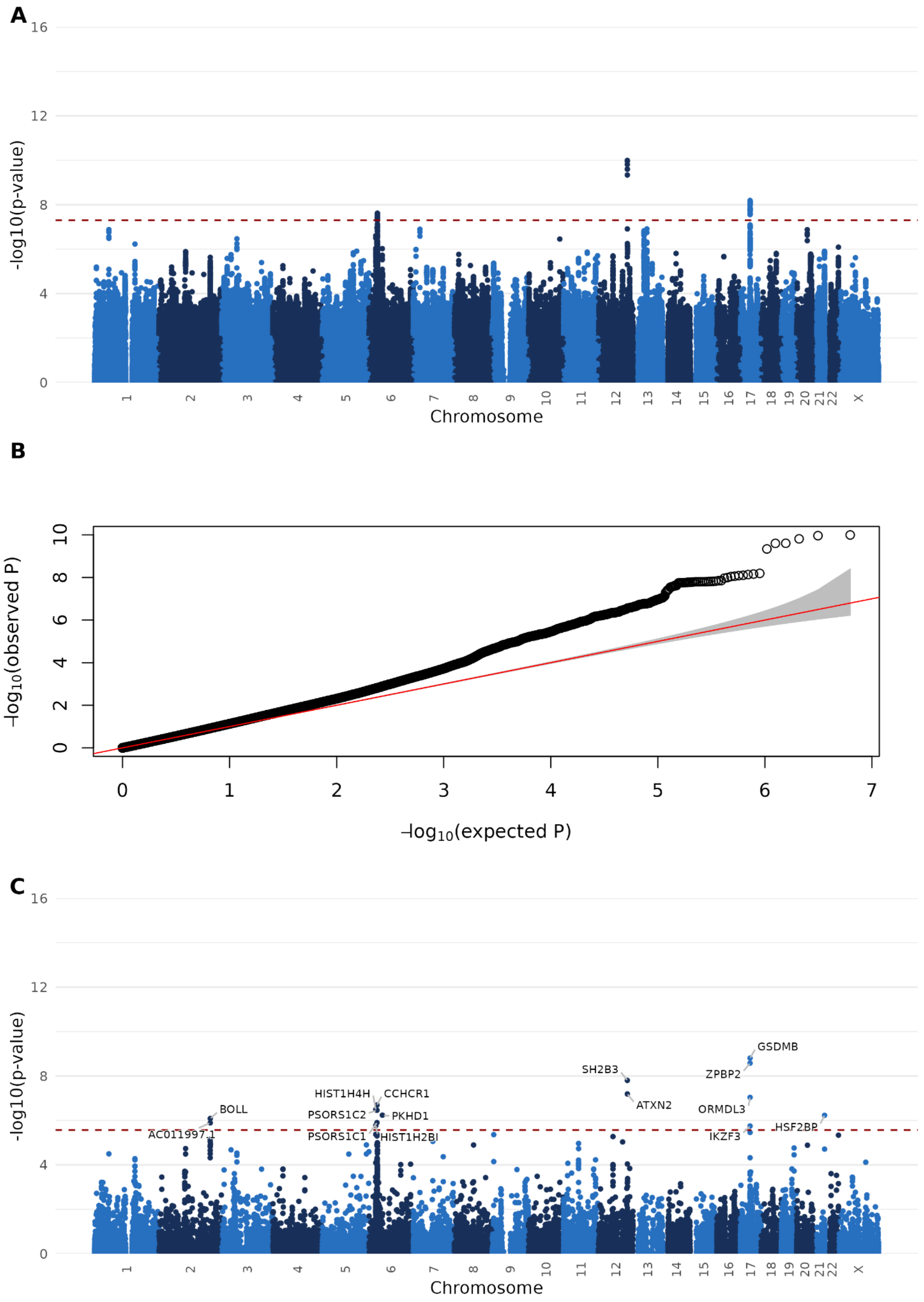

Figure S10.6. GWAS results for MDD-related Cluster 6 membership in UKB data (N = 249,167). (A) SNP-based genome-wide Manhattan plot. Association analyses were performed using linear regression to test the association between each SNP and the posterior log odds of cluster membership, controlling for age, sex, the first ten genetic principal components, and the genotyping array. In the plot, nominal p-values are displayed. The significance threshold (5×10^{-8}) is depicted with a dashed dark red line. (B) QQ-plot. (C) Gene-based genome-wide Manhattan plot. MAGMA gene-level analysis was performed to identify putative significant genes using a SNPwise-multi model, defining the SNP set of each gene with a ± 10 kb window. In the plot, nominal p-values are displayed. The statistically significant genes are indicated with labels. The significance threshold (2.7×10^{-6}) is depicted with a dashed dark red line.

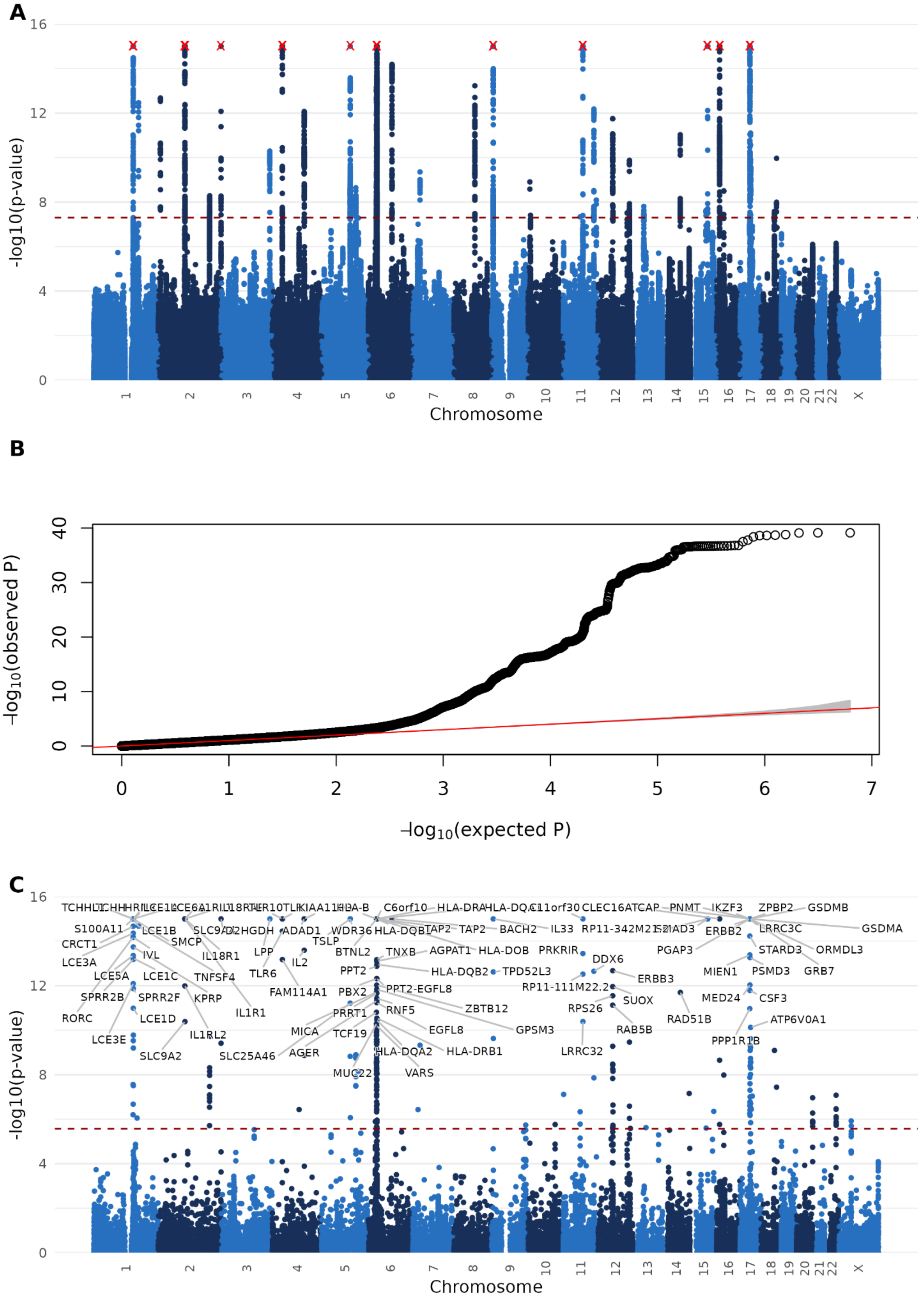

Figure S10.7. GWAS results for MDD-related Cluster 7 membership in UKB data (N = 249,167). (A) SNP-based genome-wide Manhattan plot. Association analyses were performed using linear regression to test the association between each SNP and the posterior log odds of cluster membership, controlling for age, sex, the first ten genetic principal components, and the genotyping array. In the plot, nominal p-values are displayed. Results capped at 10^{-15} are indicated with red crosses. The significance threshold ($5{\times}10^{-8}$) is depicted with a dashed dark red line. (B) QQ-plot. (C) Gene-based genome-wide Manhattan plot. MAGMA gene-level analysis was performed to identify putative significant genes using a SNPwise-multi model, defining the SNP set of each gene with a ± 10 kb window. In the plot, nominal p-values are displayed. The statistically significant genes are indicated with labels. The significance threshold ($2.7{\times}10^{-6}$) is depicted with a dashed dark red line.

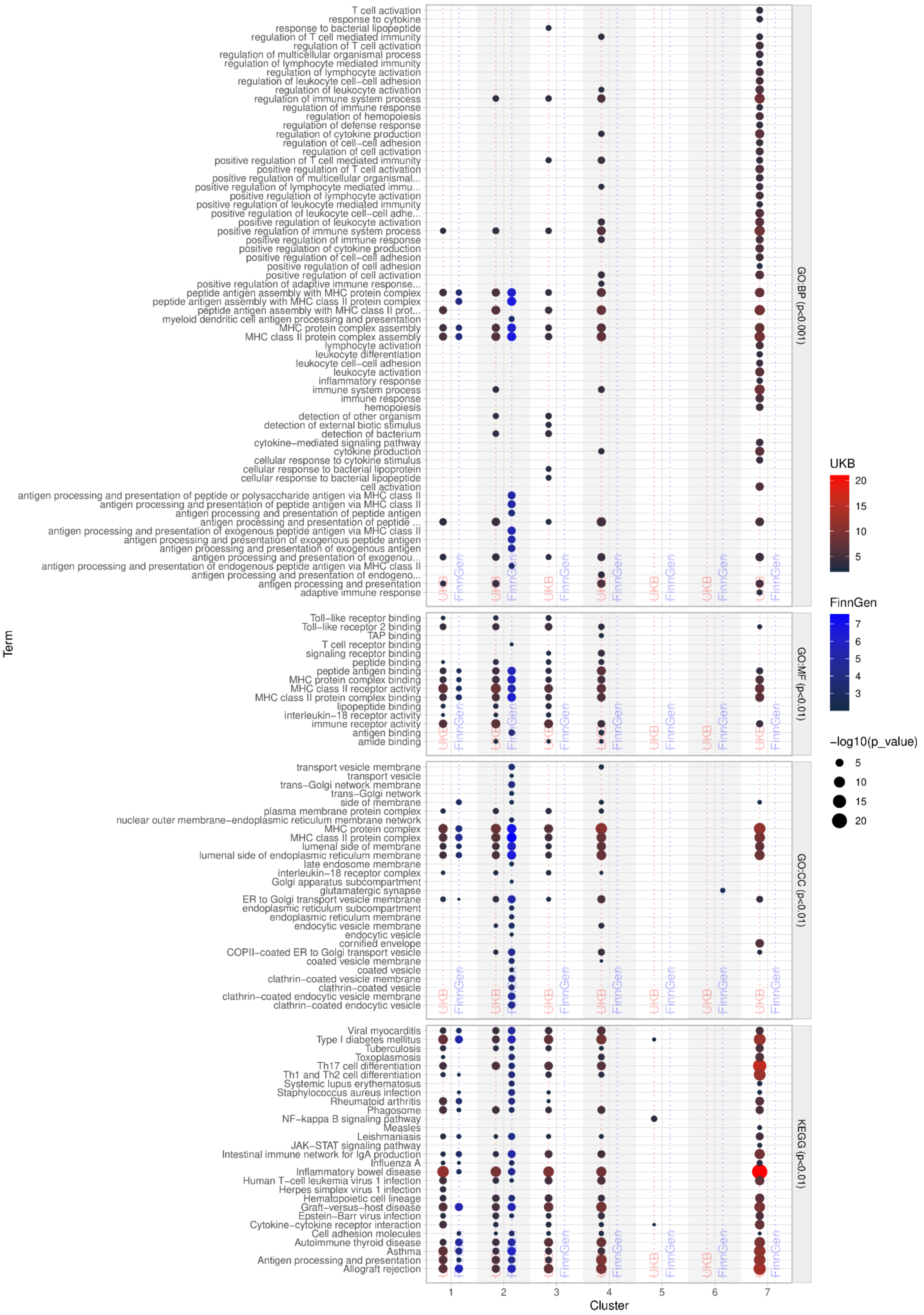

Figure S11. Functional overrepresentation analysis of UKB (N = 249,167) and FinnGen (N = 277,252) GWAS. Results of overrepresentation analysis based on the g:Profiler method. Each dot represents a statistically significantly overrepresented Gene Ontology term or KEGG pathway (adjusted *p*-value < 0.001 (for GO:BP) and adjusted *p*-value < 0.01 (for others), based on g:SCS method). The color scales correspond to the cohorts, red dots indicate UKB results (at the left part of each column), and the blue dots indicate the FinnGen results (shown at the right part of each column). The size of the dots indicates the strength of the significance, measured by the negative log-transformed *p*-values.

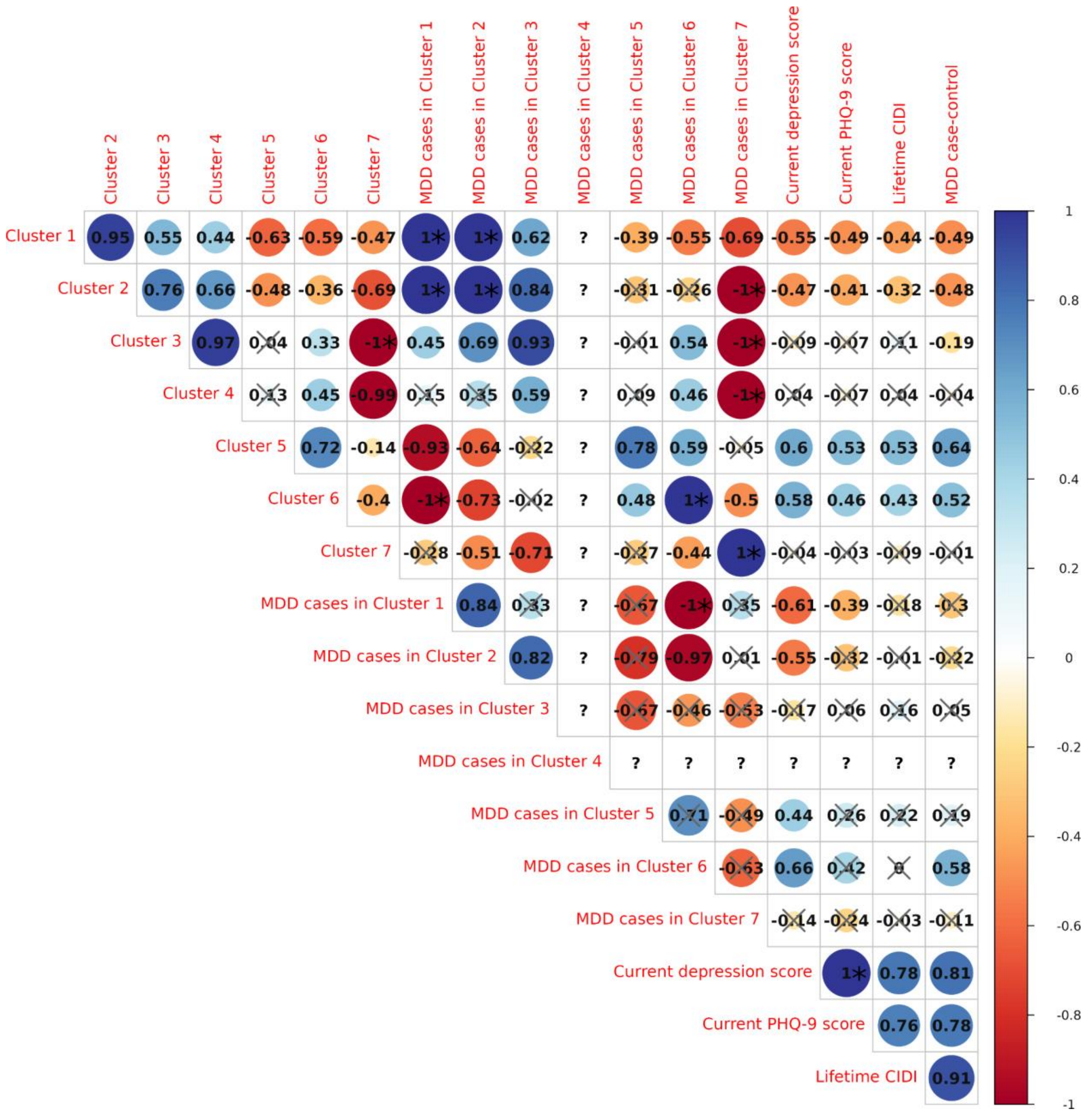

Figure S12. Genetic correlation between clusters, MDD case-only clusters, and various depression phenotypes. We performed a case-only analysis on the UKB cohort, focusing exclusively on individuals diagnosed with MDD and conducting a GWAS (N=28,853) on these filtered clusters using the same methodology as with the full population. The plot shows the genetic correlation computed with the LD Score Regression (LDSC) method among the original clusters, the MDD case-only clusters, and various depression phenotypes. Genetic correlation values not computable due to low heritability are denoted with a question mark. All estimated genetic correlations are bounded between -1 and +1, with values beyond this range marked by stars. Additionally, genetic correlations deemed not significant, following Benjamini-Hochberg correction with an adjusted *p*-value <0.05, are crossed out.

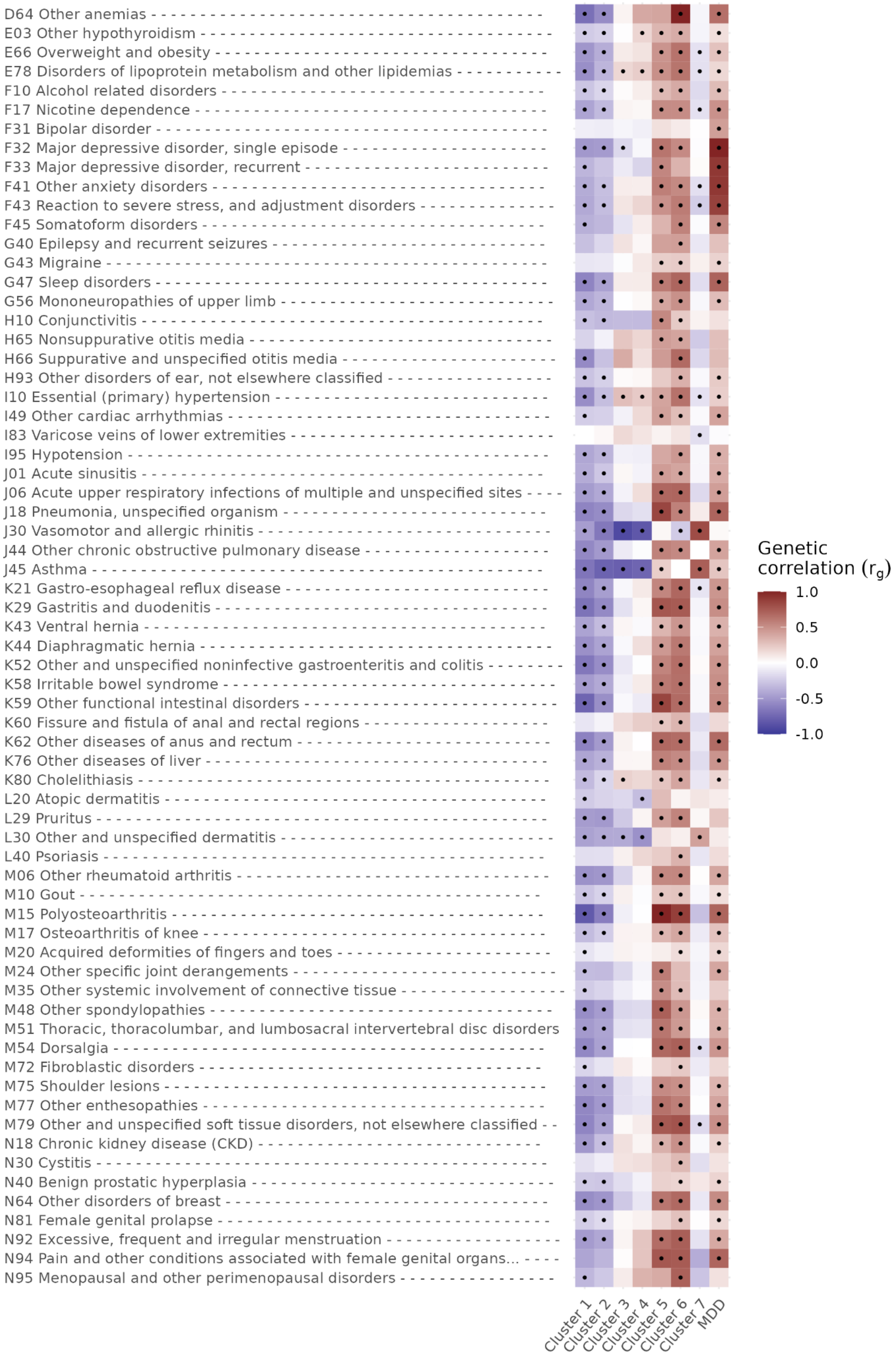

Figure S13. Genetic correlation between clusters, MDD, and the consensual diseases on which the definitions of the clusters are based (UKB, N = 249,167). First, we performed GWAS analyses using logistic regression of the binarized presence/absence of disease onset for each 86 cross-cohort disease categories, and specifically for MDD. All filters and settings were the same as for the cluster membership analysis. Next, we computed genetic correlation between the clusters and these diseases using the LDSC method. Only those diseases are shown for which MDD or at least one cluster showed a statistically significant (adjusted p-value < 0.05) genetic correlation. p-values were adjusted using Benjamini-Hochberg method. Significant genetic correlations are indicated with dots. The GWAS of the cluster memberships and all diseases were performed on the UKB cohort using the same covariates and filtering procedure (see Methods).

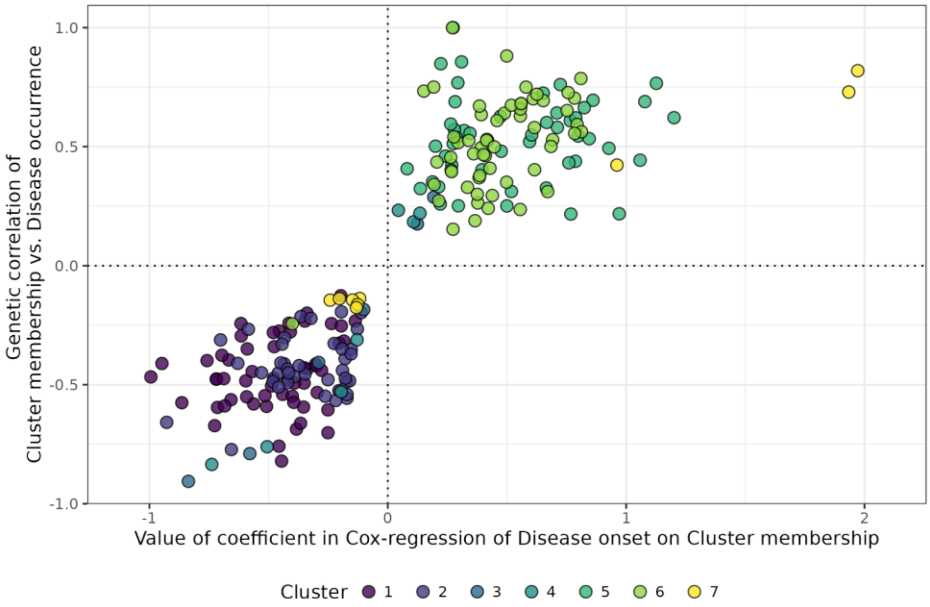

Figure S14. Comparison of the value of the coefficient in Cox regression of cluster membership on disease onset versus the genetic correlation between the cluster membership and the disease occurrence in the UKB data (N = 249,167). The **x axis** shows the value of coefficient in Cox regression of disease onset on cluster membership. To compute these, we used weighted Cox regression to determine the disease outcomes in the various clusters (i.e., the hazard ratio of cluster membership regarding disease occurrence). Specifically, for each cluster, we constructed Cox proportional hazard models, where the independent variable for a specific individual was a dummy variable created in the following way. We counted each participant twice, summing to a weight of 1. First, we set the value of the dummy variable to 1 and weighted this sample by the posterior probability of cluster membership. Next, we set the value of the dummy variable to 0 and used a weight for this sample equal to 1 minus the probability of cluster membership. The covariates were sex, household income, and the normalized birth year. The dependent variable was disease onset. Participants were right censored for a given target disease at their age if the disease was not diagnosed. We calculated separate models for each cross-cohort disease. P-values of the cluster membership variables were adjusted separately for each cohort using the Benjamini–Hochberg method. The **y axis** shows the genetic correlation between the cluster membership and the occurrence of a cross-cohort disease. To compute these, we performed GWAS analyses using logistic regression of the binarized presence/absence of disease onset for each 86 cross-cohort disease categories, and for MDD. All filters and settings were the same as for the cluster membership analysis. Next, we computed genetic correlation between the clusters and these diseases using the LDSC method. Each point represents a disease according to a given cluster (indicated by the color of the point). Only those diseases are shown that are statistically significant according to both methods.

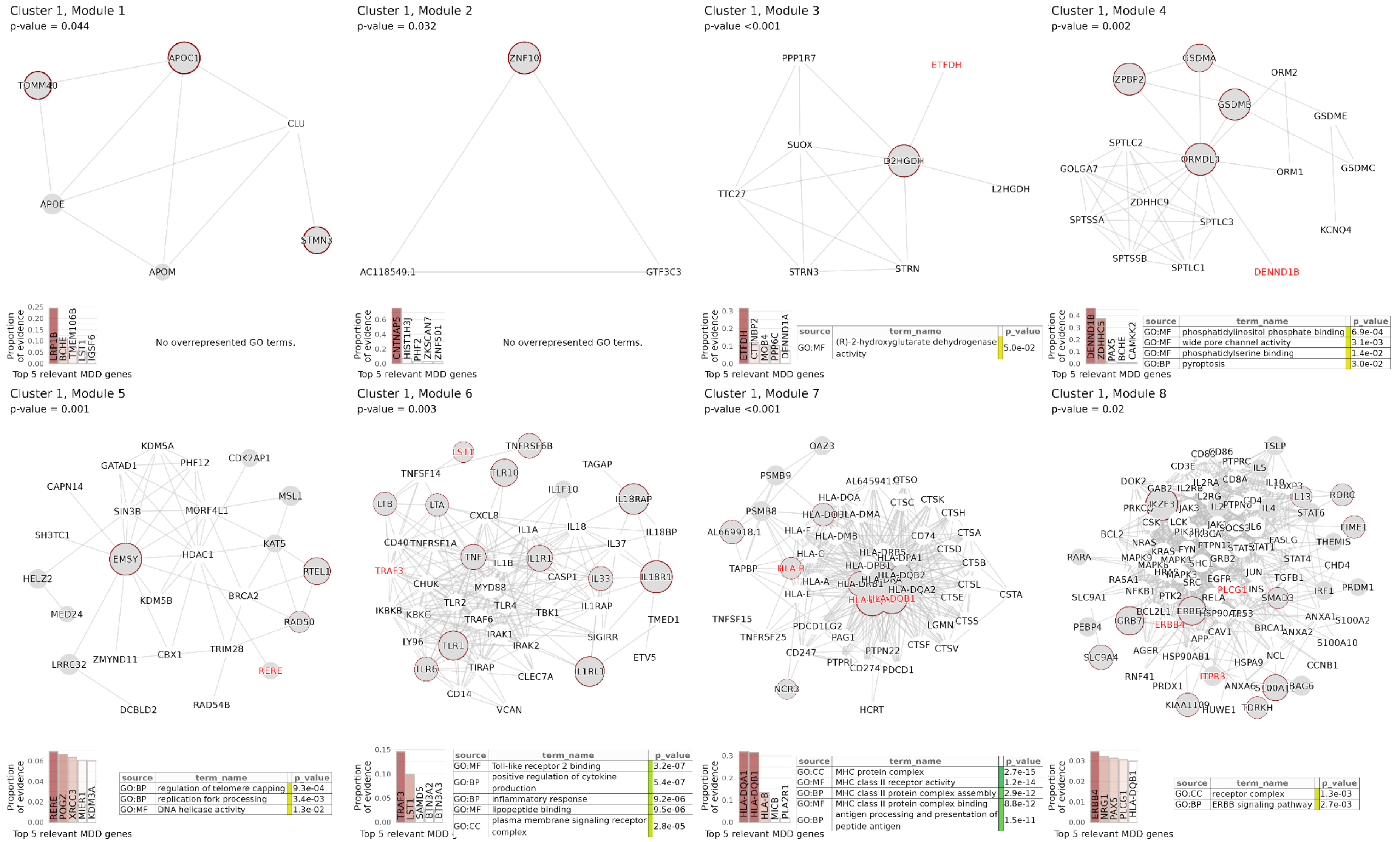

Figure S15.1. Functional modules of the human interactome in Cluster 1 that are pleiotropic with MDD (according to UKB data, N = 249,167). Top: Pleiotropic functional modules. Gray nodes were used as evidence nodes in a network propagation algorithm used to score all genes in the STRING PPI network. The size of the nodes indicates the propagated score. Nodes with red borders are statistically significant according to the GWAS of Cluster 1. Modules of the top-scoring genes were identified, and those modules are shown in which the propagated effect of MDD-associated genes (shown with red labels, according to a GWAS meta-analysis of Howard et al.) are significantly enriched. Bottom left: The relative contribution of the top 5 MDD-associated genes to the module. Bottom right: The at most top 5 most relevant statistically significant Gene Ontology terms overrepresented in the module. See Methods for details.

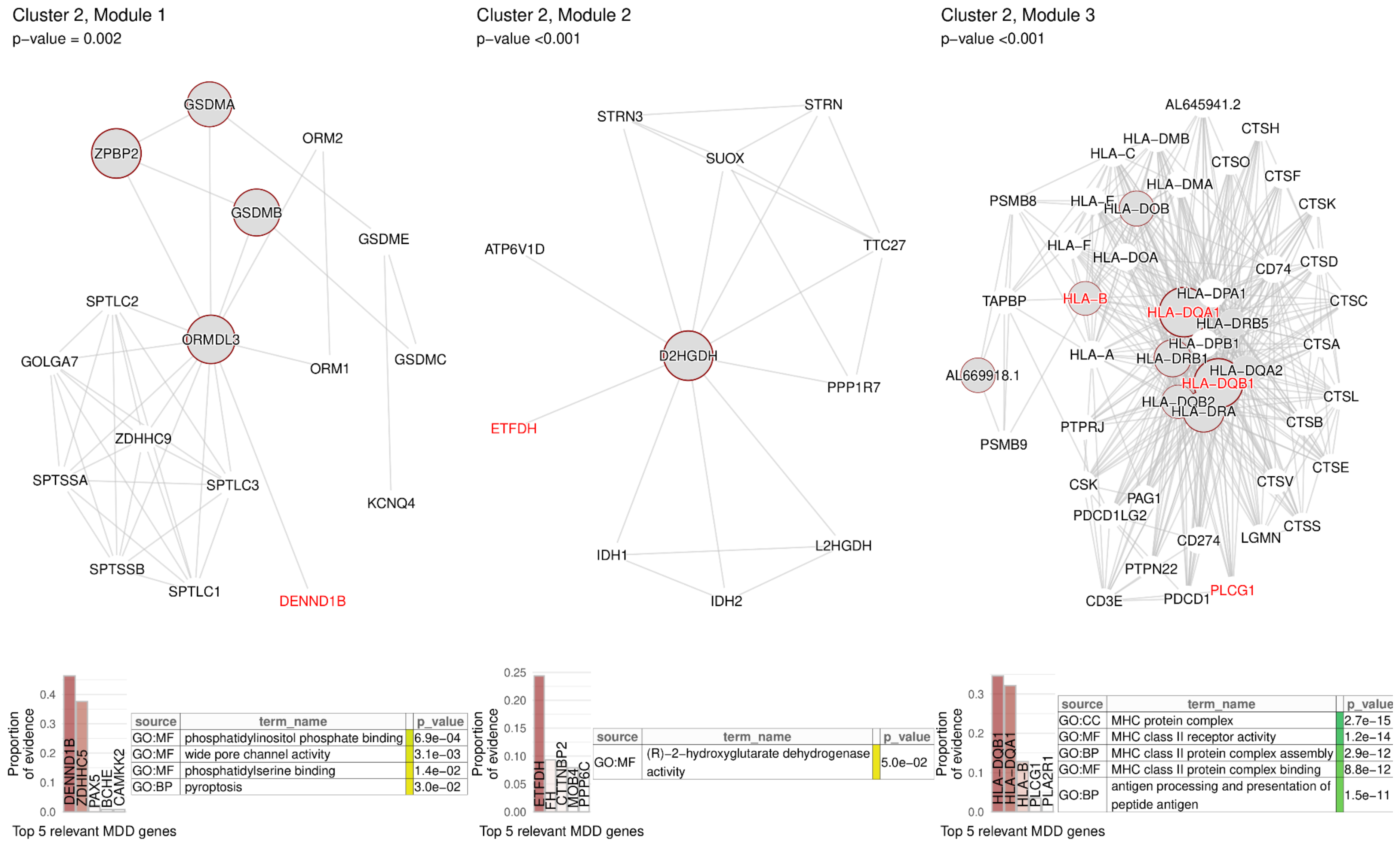

Figure S15.2. Functional modules of the human interactome in Cluster 2 that are pleiotropic with MDD. See the caption of Figure S15.1. for details.

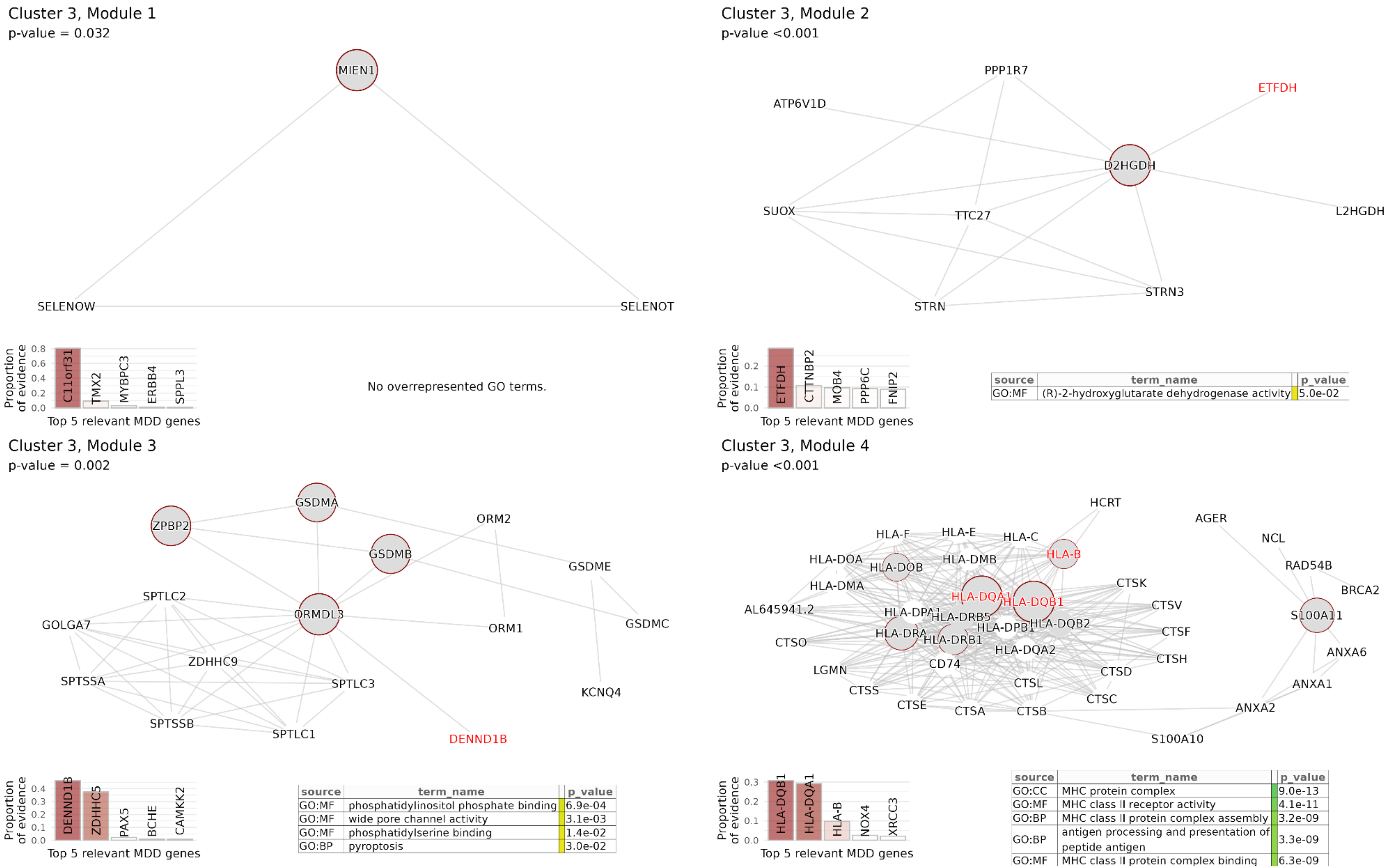

Figure S15.3. Functional modules of the human interactome in Cluster 3 that are pleiotropic with MDD. See the caption of Figure S15.1. for details.

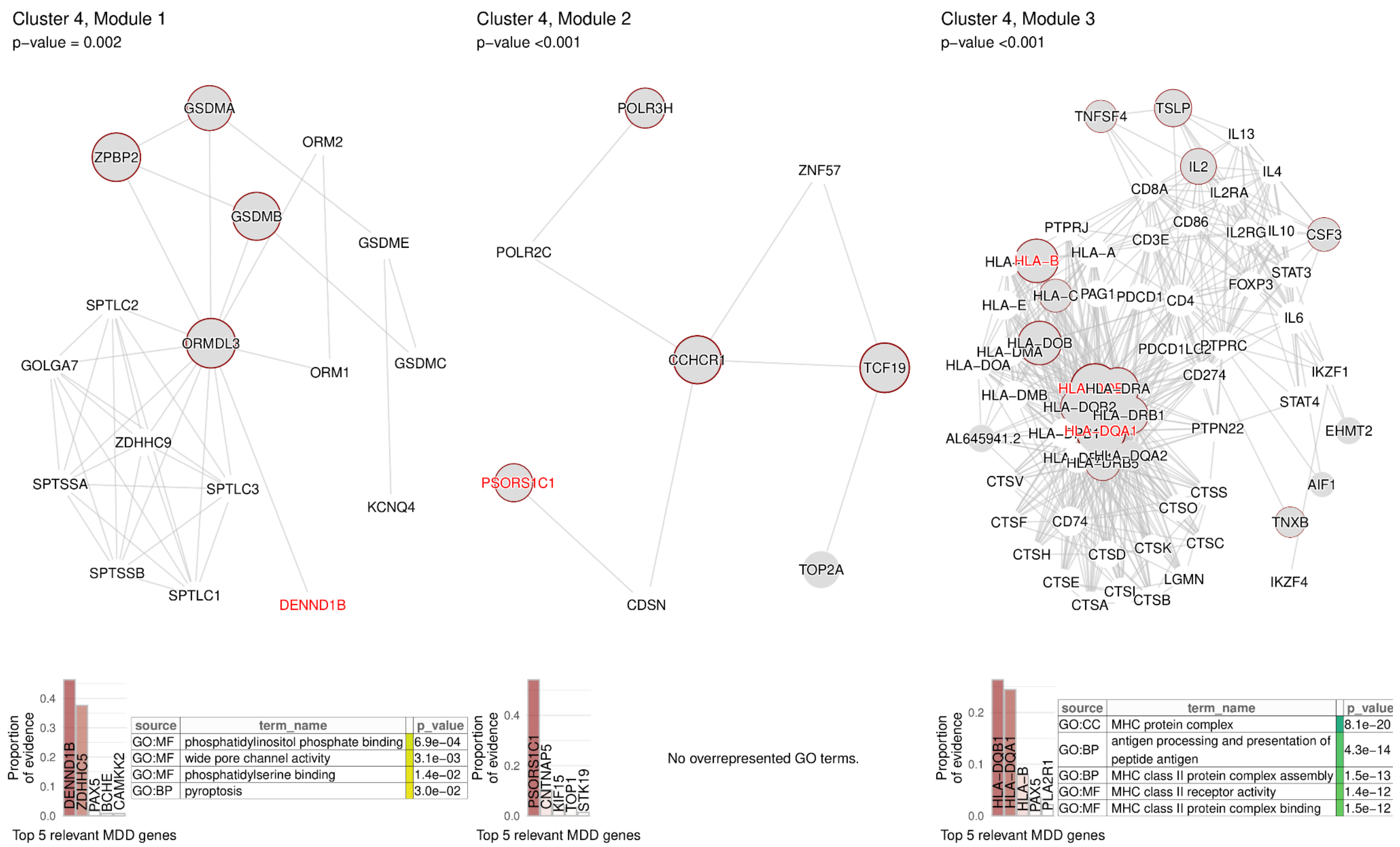

source	term_name	p_value
GO:MF	phosphatidylinositol phosphate binding	6.9e–04
GO:MF	wide pore channel activity	3.1e–03
GO:MF	phosphatidylserine binding	1.4e–02
GO:BP	pyroptosis	3.0e–02

source	term_name	p_value
GO:CC	MHC protein complex	8.1e–20
GO:BP	antigen processing and presentation of peptide antigen	4.3e–14
GO:BP	MHC class II protein complex assembly	1.5e–13
GO:MF	MHC class II receptor activity	1.4e–12
GO:MF	MHC class II protein complex binding	1.5e–12

Figure S15.4. Functional modules of the human interactome in Cluster 4 that are pleiotropic with MDD. See the caption of Figure S15.1. for details.

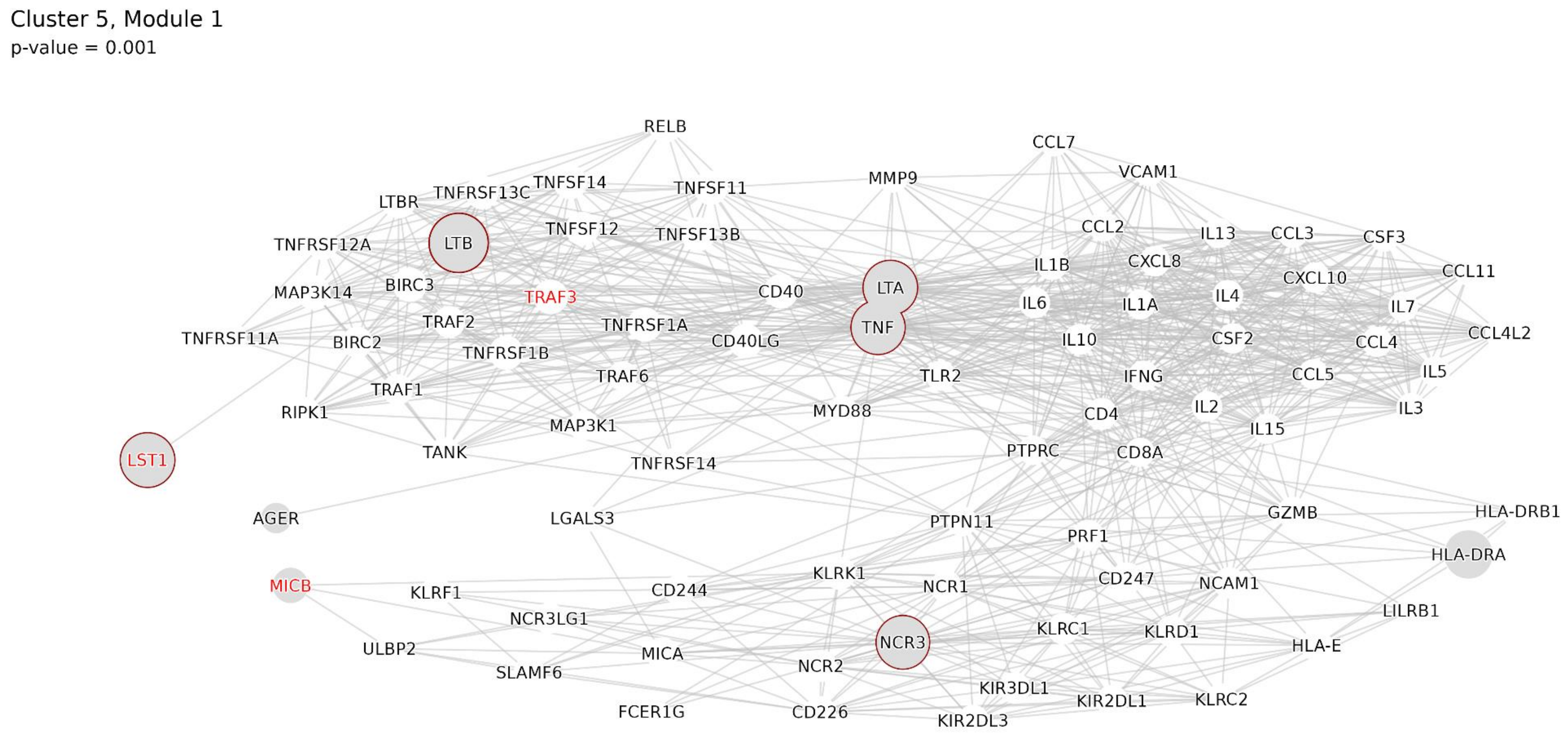

source	term_name		p_value
GO:MF	tumor necrosis factor receptor binding		6.5e-04
GO:CC	plasma membrane		1.7e-02
GO:BP	regulation of immune system process		4.1e-02

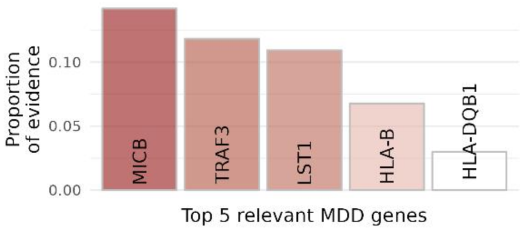

Figure S15.5. Functional module of the human interactome in Cluster 5 that is pleiotropic with MDD. See the caption of Figure S15.1. for details.

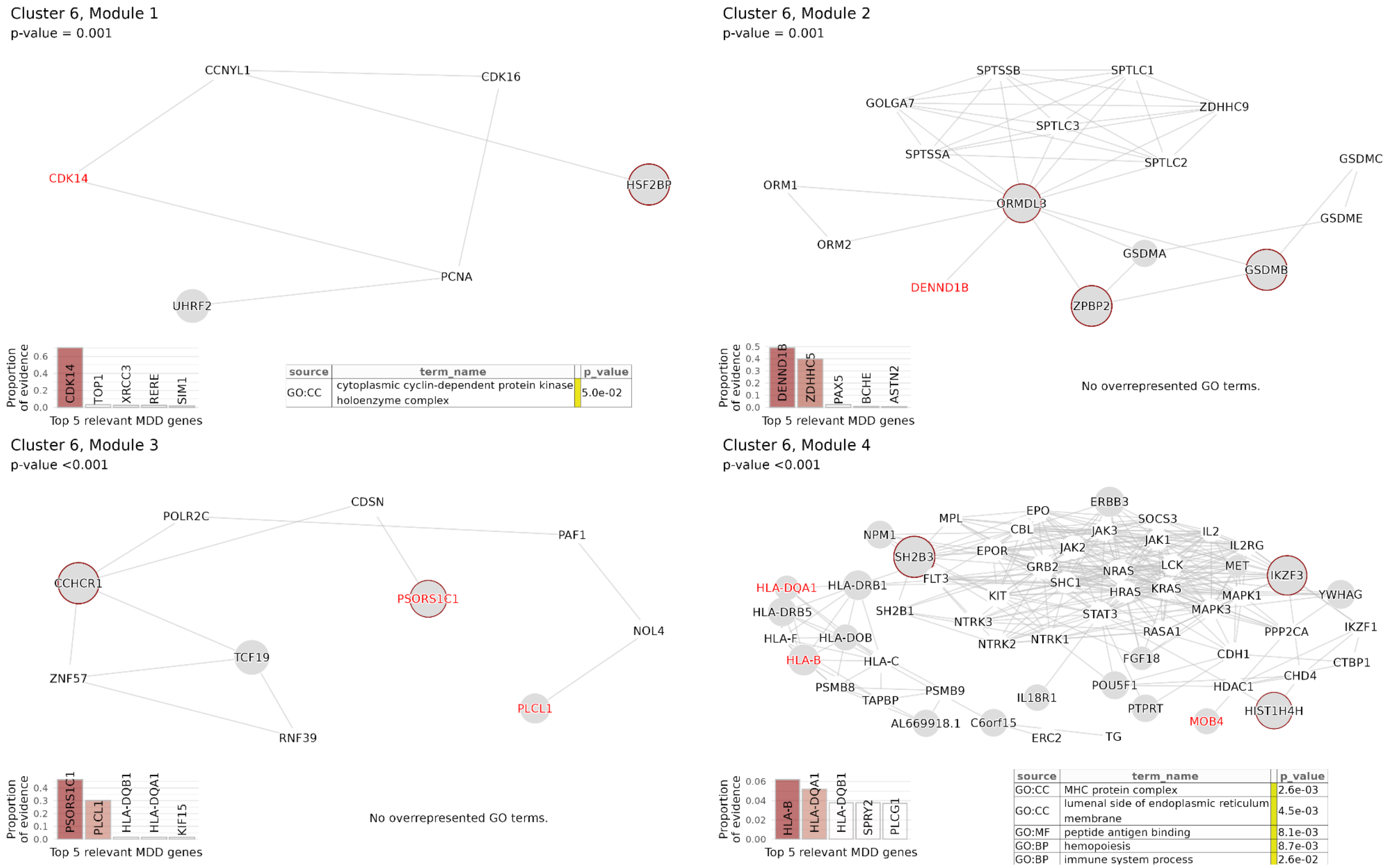

source	term_name	p_value
GO:CC	cytoplasmic cyclin-dependent protein kinase holoenzyme complex	5.0e-02

source	term_name	p_value
GO:CC	MHC protein complex	2.6e-03
GO:CC	lumenal side of endoplasmic reticulum membrane	4.5e-03
GO:MF	peptide antigen binding	8.1e-03
GO:BP	hemopoiesis	8.7e-03
GO:BP	immune system process	2.6e-02

Figure S15.6. Functional modules of the human interactome in Cluster 6 that are pleiotropic with MDD. See the caption of Figure S15.1. for details.

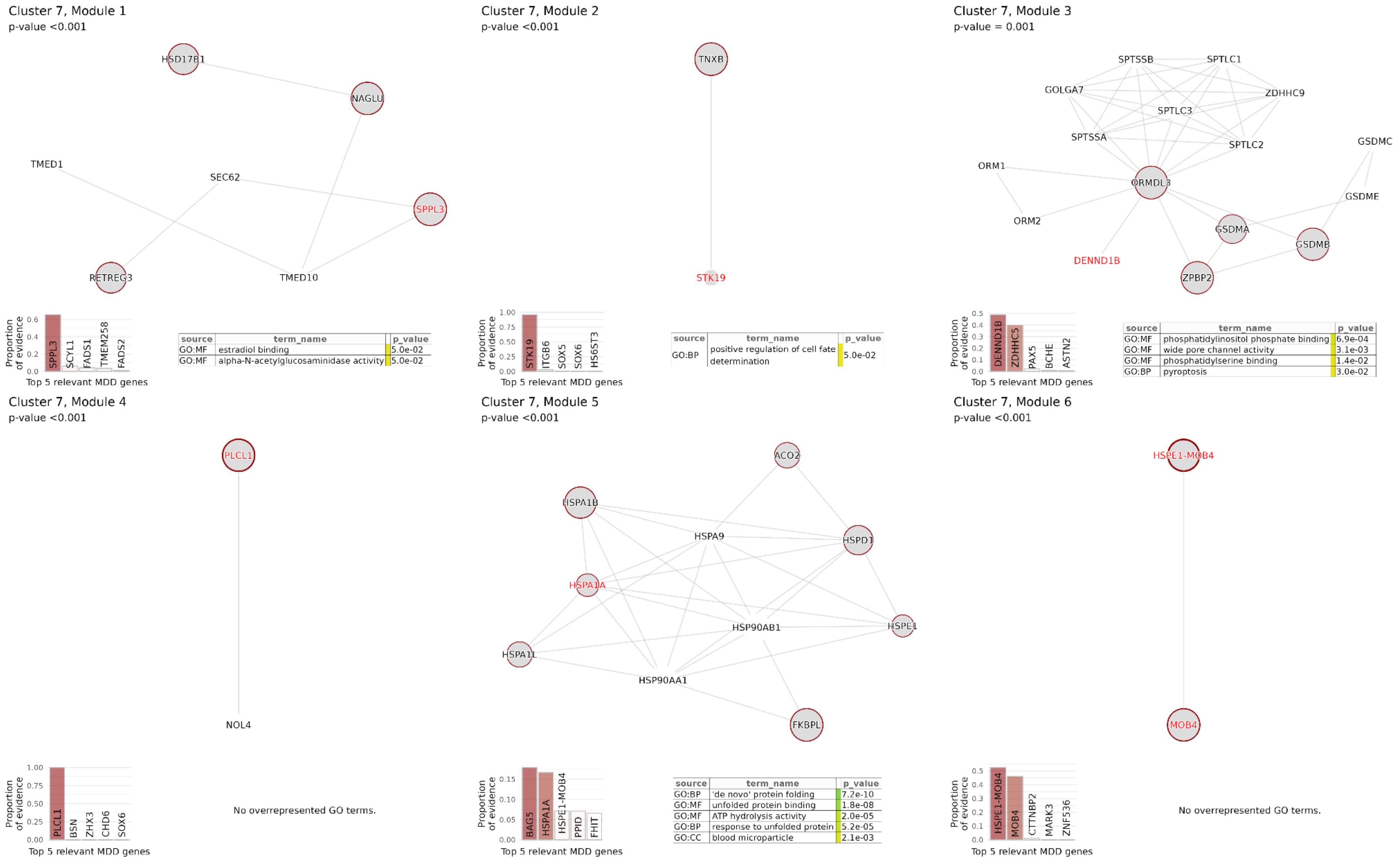

Figure S15.7a. Functional modules of the human interactome in Cluster 7 that are pleiotropic with MDD. See the caption of Figure S15.1. for details.

Cluster 7, Module 7

p-value = 0.007

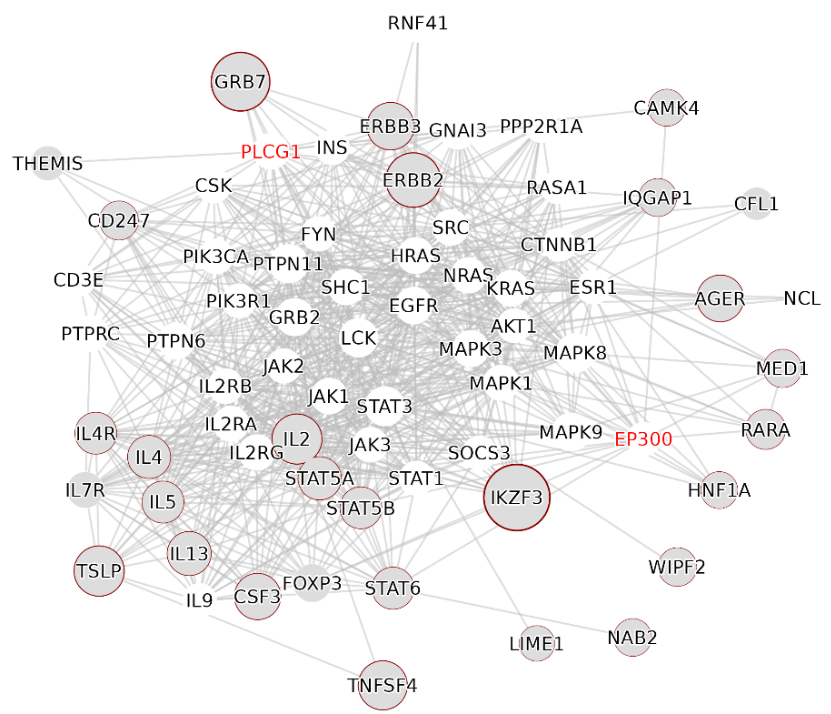

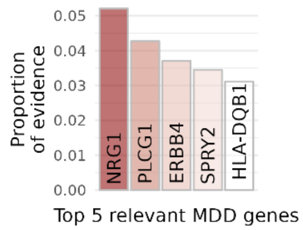

source	term_name	p_value
GO:BP	positive regulation of biological process	2.0e-08
GO:BP	regulation of hemopoiesis	2.9e-08
GO:MF	cytokine receptor binding	1.9e-05
GO:MF	transcription coactivator binding	9.4e-05
GO:CC	ERBB3:ERBB2 complex	4.8e-04

Cluster 7, Module 8

p-value <0.001

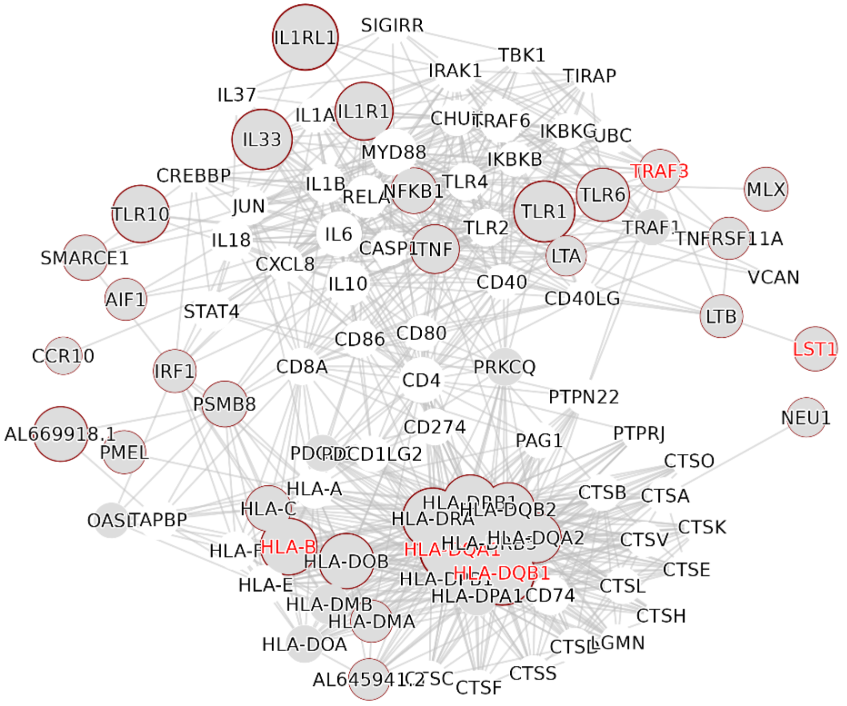

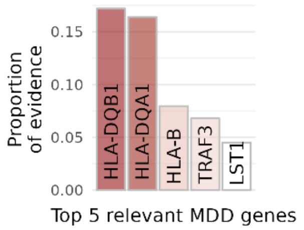

source	term_name	p_value
GO:CC	MHC protein complex	2.8e-21
GO:BP	MHC class II protein complex assembly	1.4e-16
GO:BP	positive regulation of immune system process	2.0e-15
GO:MF	MHC class II protein complex binding	8.5e-15
GO:MF	MHC class II receptor activity	1.8e-13

Figure S15.7b. Functional modules of the human interactome in Cluster 7 that are pleiotropic with MDD. See the caption of Figure S15.1. for details.

Supplementary Figures for results section

Non-genetic risk-factor profiles of MDD-related multimorbidity clusters in the UKB cohort

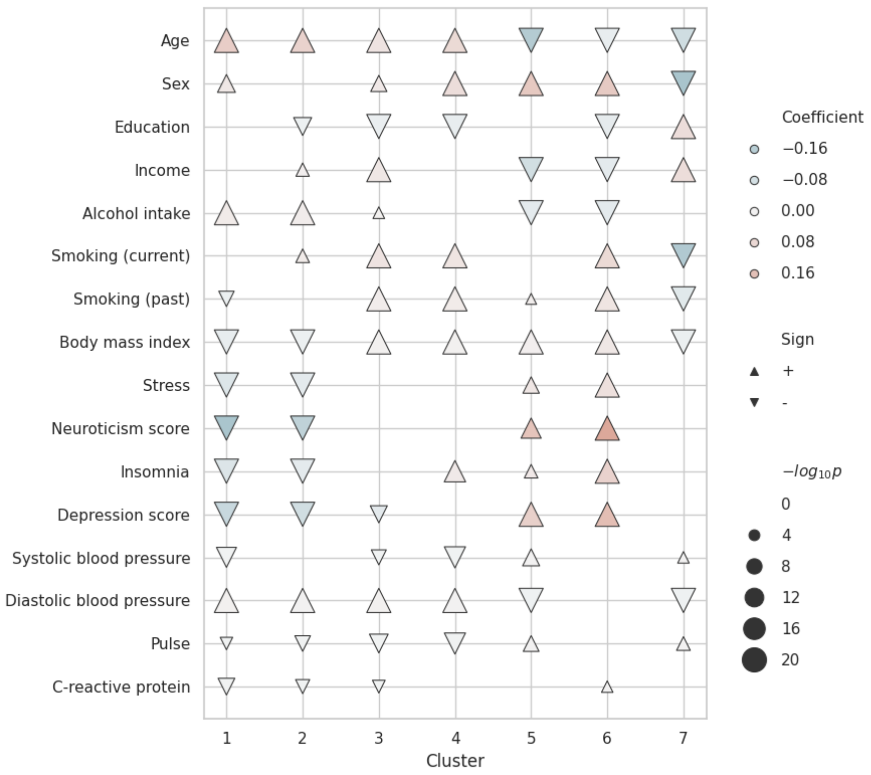

Figure S16. Complex linear regression models involving available non-genetic factors included in the UK Biobank dataset for each cluster (N = 249,167). The "complex regression model" uses all available risk factors at once in a single model. The posterior log odds of being in a given cluster serves as the target variable. The direction of the triangles corresponds to the sign of the coefficient (positive - upwards; negative - downwards) and the color indicated the magnitude. The size of the triangles is proportional to the -log10 *p*-value, and only nominally significant values are shown (-log10*p* > 4). CRP: C-reactive protein; bp: blood pressure; sex: 1-males, 2-females.

Supplementary Figures for results section

Validation of MDD-related multimorbidity profiles at the genetic and non-genetic risk-factor levels

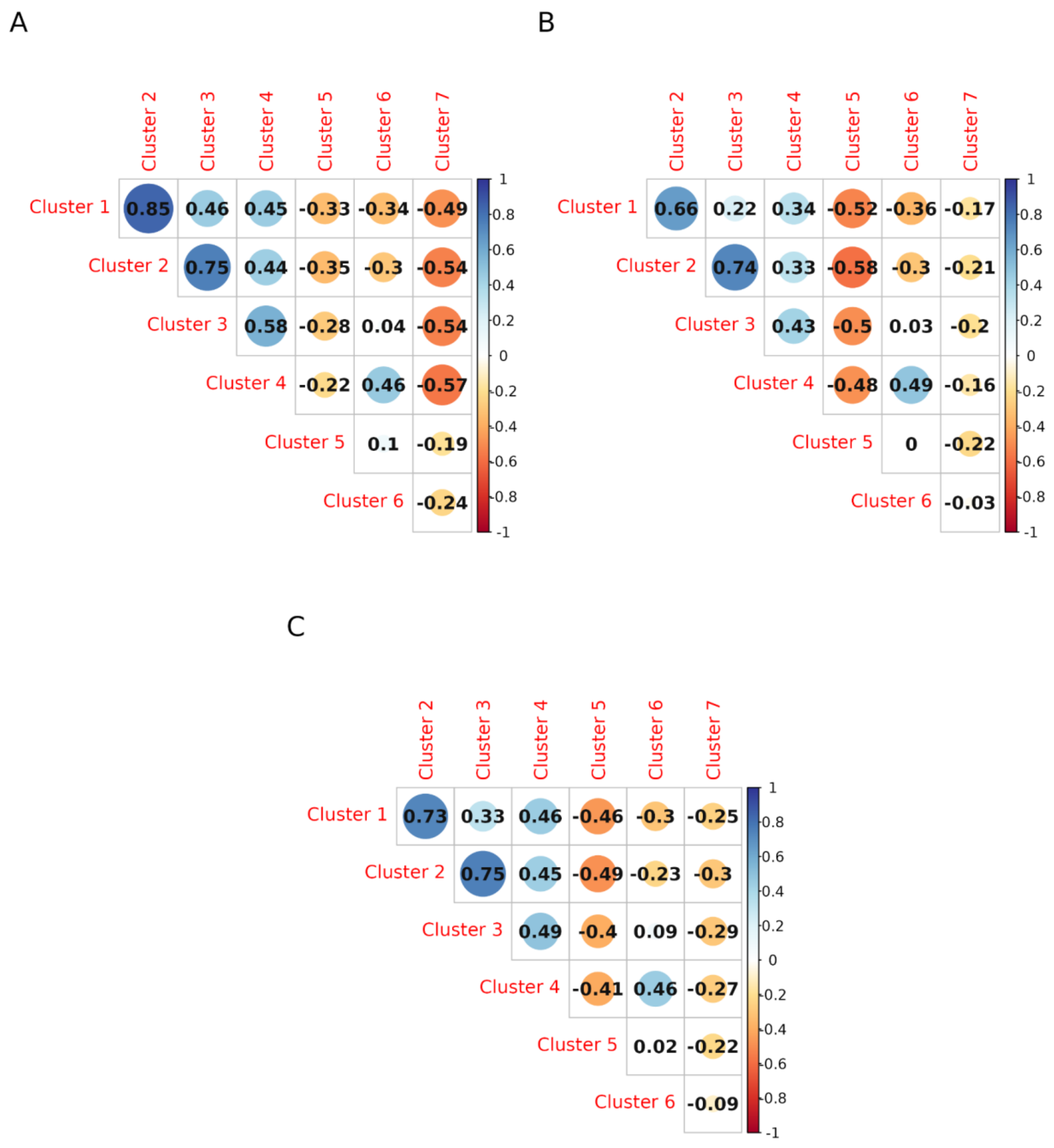

Figure S17. Pearson correlation pattern of GWAS derived beta values between MDD-related clusters. (A) UK Biobank (N = 249,167); (B) THL (N = 23,786); (C) FinnGen (N = 277,252).

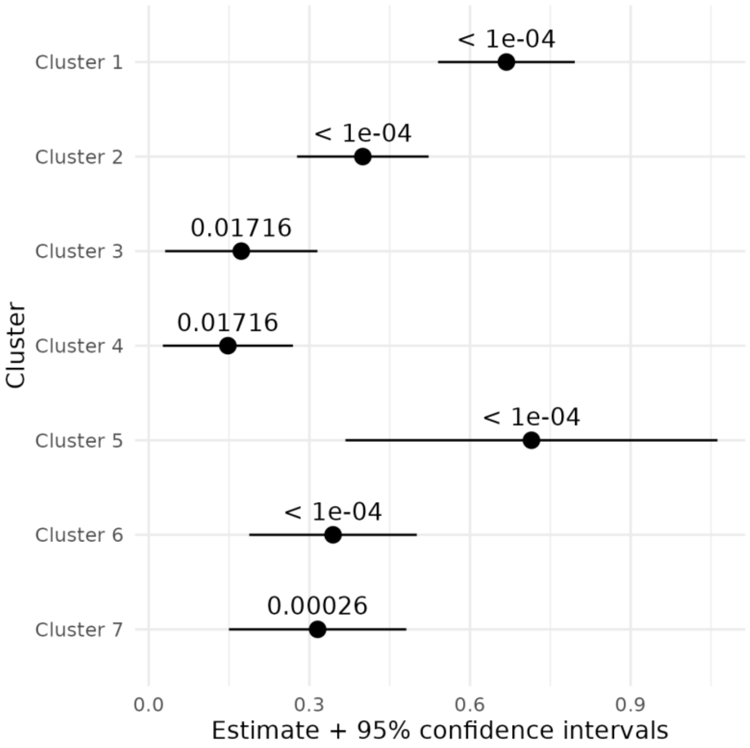

Figure S18. Results for polygenic risk score (PRS) analysis in THL (N = 30,961). Results of linear regression analyses using posterior log-odds cluster membership as the dependent variable and PRS for cluster membership based on UKB GWAS results as predictor variable for each of the seven clusters. Adjusted for age, sex, cohort, batch, east/west-background. Estimated coefficients and 95% confidence intervals are shown. P-values were adjusted using Benjamini-Hochberg method and are shown above the point estimates.

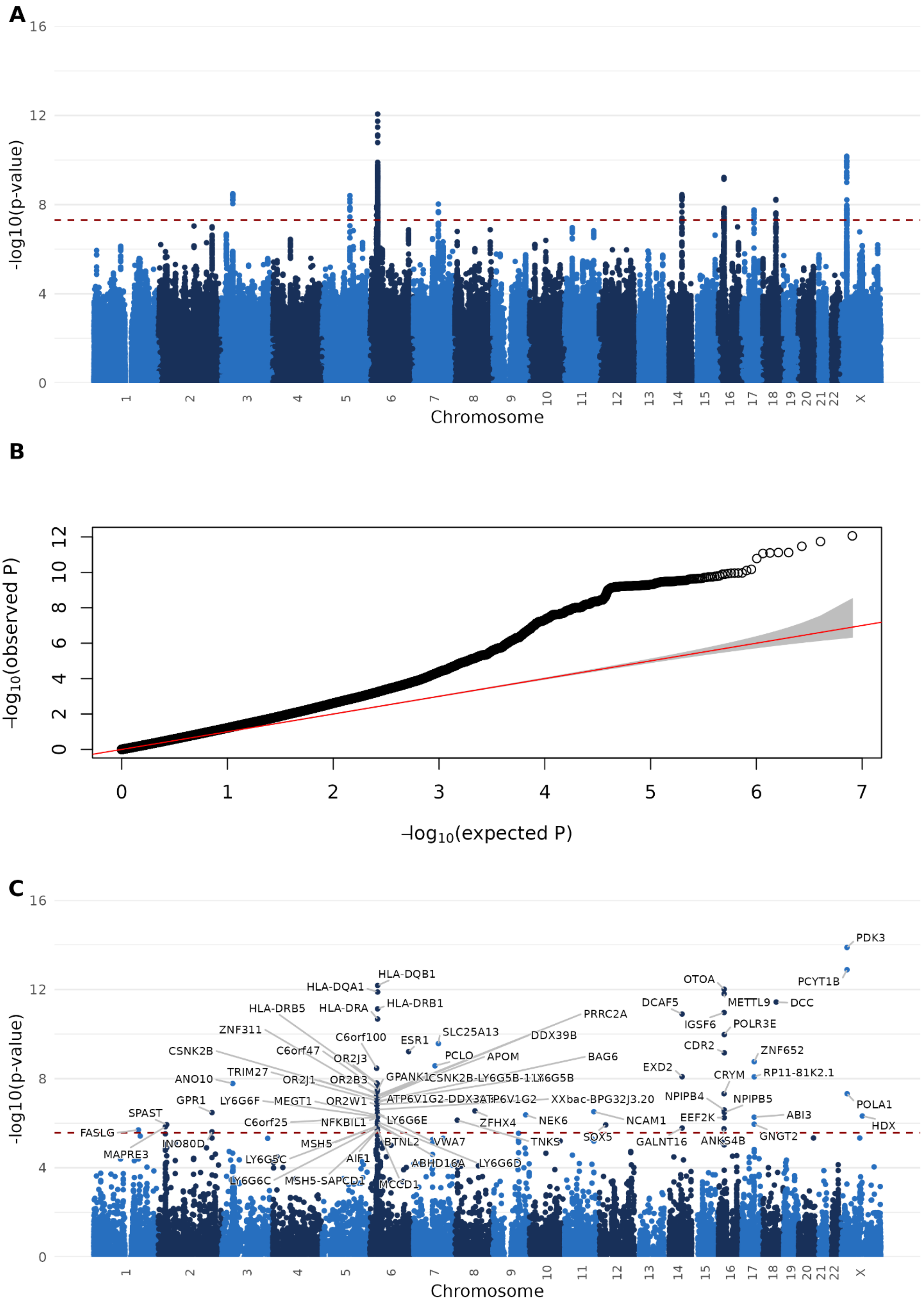

Figure S19.1. GWAS results for MDD-related Cluster 1 membership in FinnGen data (N = 277,252). (A) SNP-based genome-wide Manhattan plot. Association analyses were performed using linear regression to test the association between each SNP and the posterior log odds of cluster membership, controlling for age, sex, the first ten genetic principal components, and the genotyping array. In the plot, nominal p-values are displayed. The significance threshold (5×10^{-8}) is depicted with a dashed dark red line. (B) QQ-plot. (C) Gene-based genome-wide Manhattan plot. MAGMA gene-level analysis was performed to identify putative significant genes using a SNPwise-multi model, defining the SNP set of each gene with a ± 10 kb window. In the plot, nominal p-values are displayed. The statistically significant genes are indicated with labels. The significance threshold (2.7×10^{-6}) is depicted with a dashed dark red line.

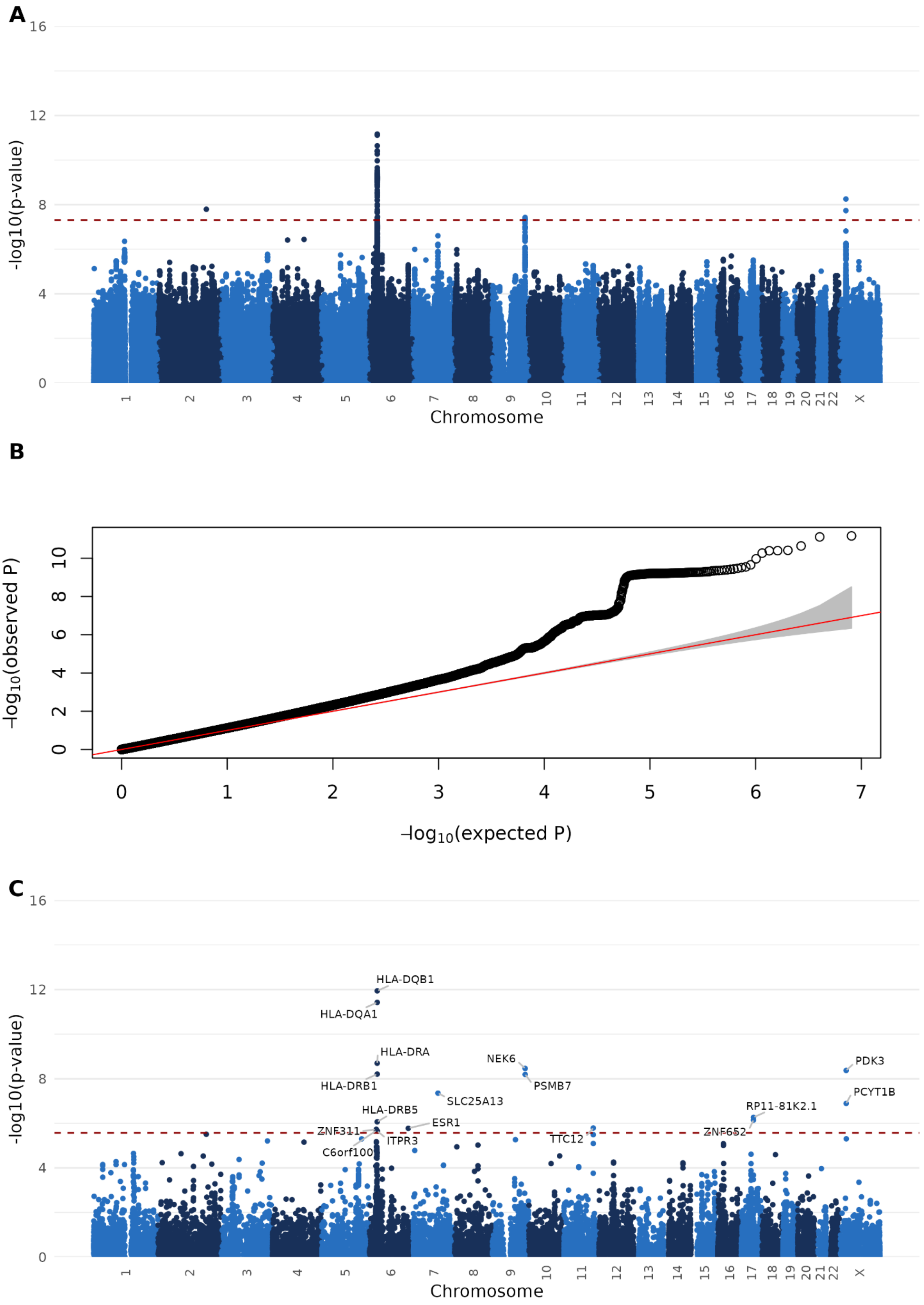

Figure S19.2. GWAS results for MDD-related Cluster 2 membership in FinnGen data (N = 277,252). (A) SNP-based genome-wide Manhattan plot. Association analyses were performed using linear regression to test the association between each SNP and the posterior log odds of cluster membership, controlling for age, sex, the first ten genetic principal components, and the genotyping array. In the plot, nominal p-values are displayed. The significance threshold (5×10^{-8}) is depicted with a dashed dark red line. (B) QQ-plot. (C) Gene-based genome-wide Manhattan plot. MAGMA gene-level analysis was performed to identify putative significant genes using a SNPwise-multi model, defining the SNP set of each gene with a ± 10 kb window. In the plot, nominal p-values are displayed. The statistically significant genes are indicated with labels. The significance threshold (2.7×10^{-6}) is depicted with a dashed dark red line.

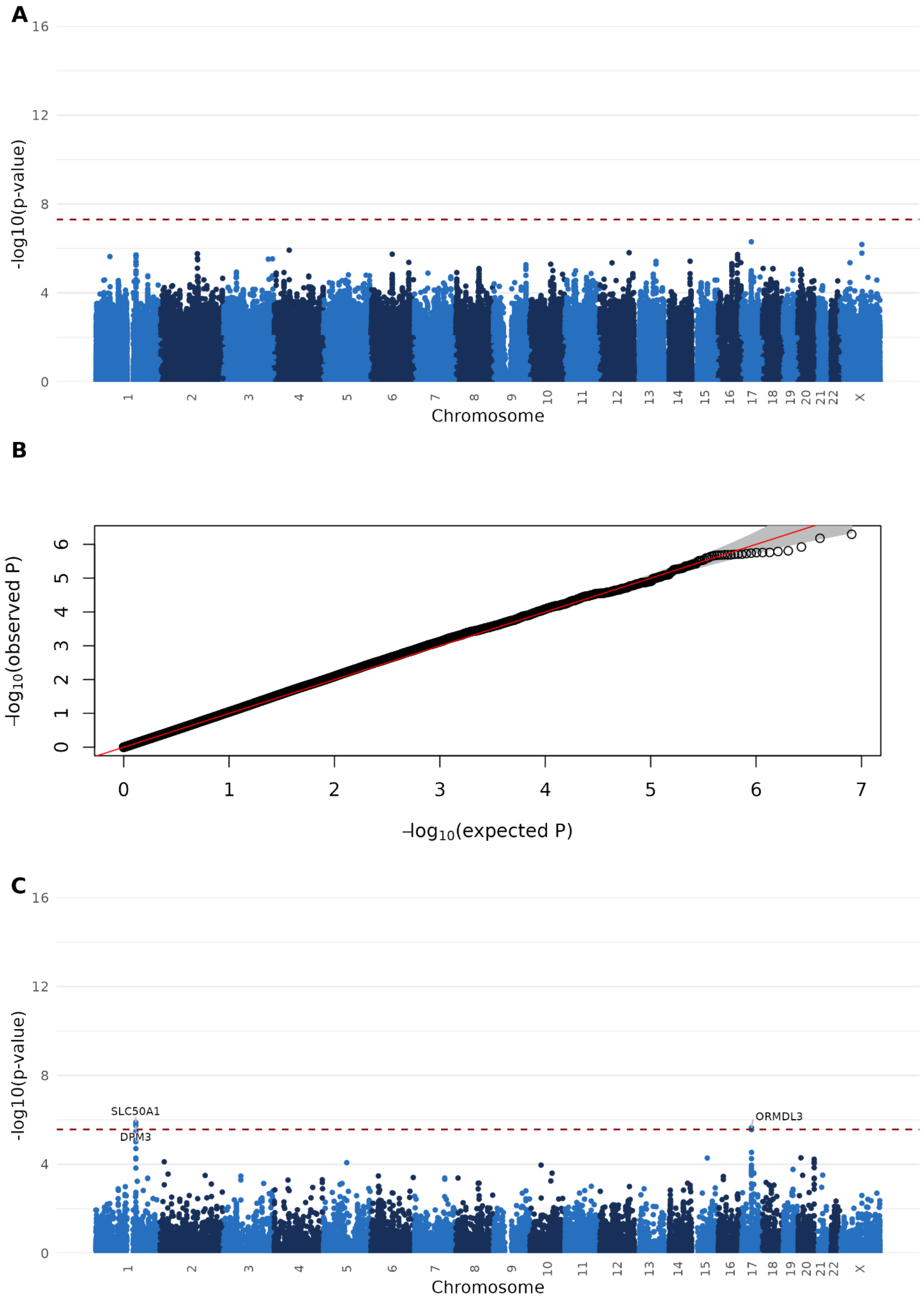

Figure S19.3. GWAS results for MDD-related Cluster 3 membership in FinnGen data (N = 277,252). (A) SNP-based genome-wide Manhattan plot. Association analyses were performed using linear regression to test the association between each SNP and the posterior log odds of cluster membership, controlling for age, sex, the first ten genetic principal components, and the genotyping array. In the plot, nominal p-values are displayed. Results capped at 10^{-15} are indicated with red crosses. The significance threshold (5×10^{-8}) is depicted with a dashed dark red line. (B) QQ-plot. (C) Gene-based genome-wide Manhattan plot. MAGMA gene-level analysis was performed to identify putative significant genes using a SNPwise-multi model, defining the SNP set of each gene with a ± 10 kb window. In the plot, nominal p-values are displayed. The statistically significant genes are indicated with labels. The significance threshold (2.7×10^{-6}) is depicted with a dashed dark red line.

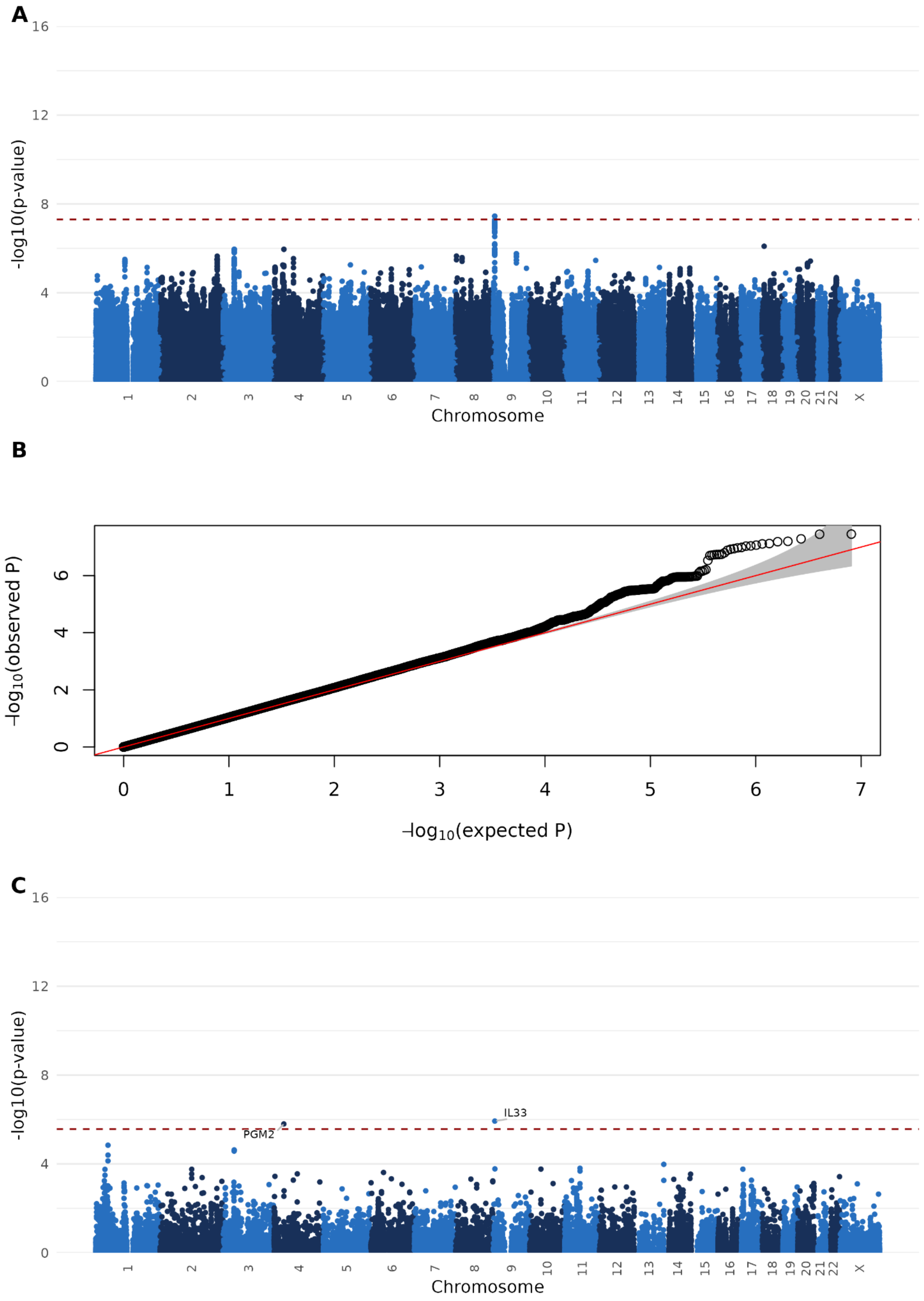

Figure S19.4. GWAS results for MDD-related Cluster 4 membership in FinnGen data (N = 277,252). (A) SNP-based genome-wide Manhattan plot. Association analyses were performed using linear regression to test the association between each SNP and the posterior log odds of cluster membership, controlling for age, sex, the first ten genetic principal components, and the genotyping array. In the plot, nominal p-values are displayed. The significance threshold (5×10^{-8}) is depicted with a dashed dark red line. (B) QQ-plot. (C) Gene-based genome-wide Manhattan plot. MAGMA gene-level analysis was performed to identify putative significant genes using a SNPwise-multi model, defining the SNP set of each gene with a ± 10 kb window. In the plot, nominal p-values are displayed. The statistically significant genes are indicated with labels. The significance threshold (2.7×10^{-6}) is depicted with a dashed dark red line.

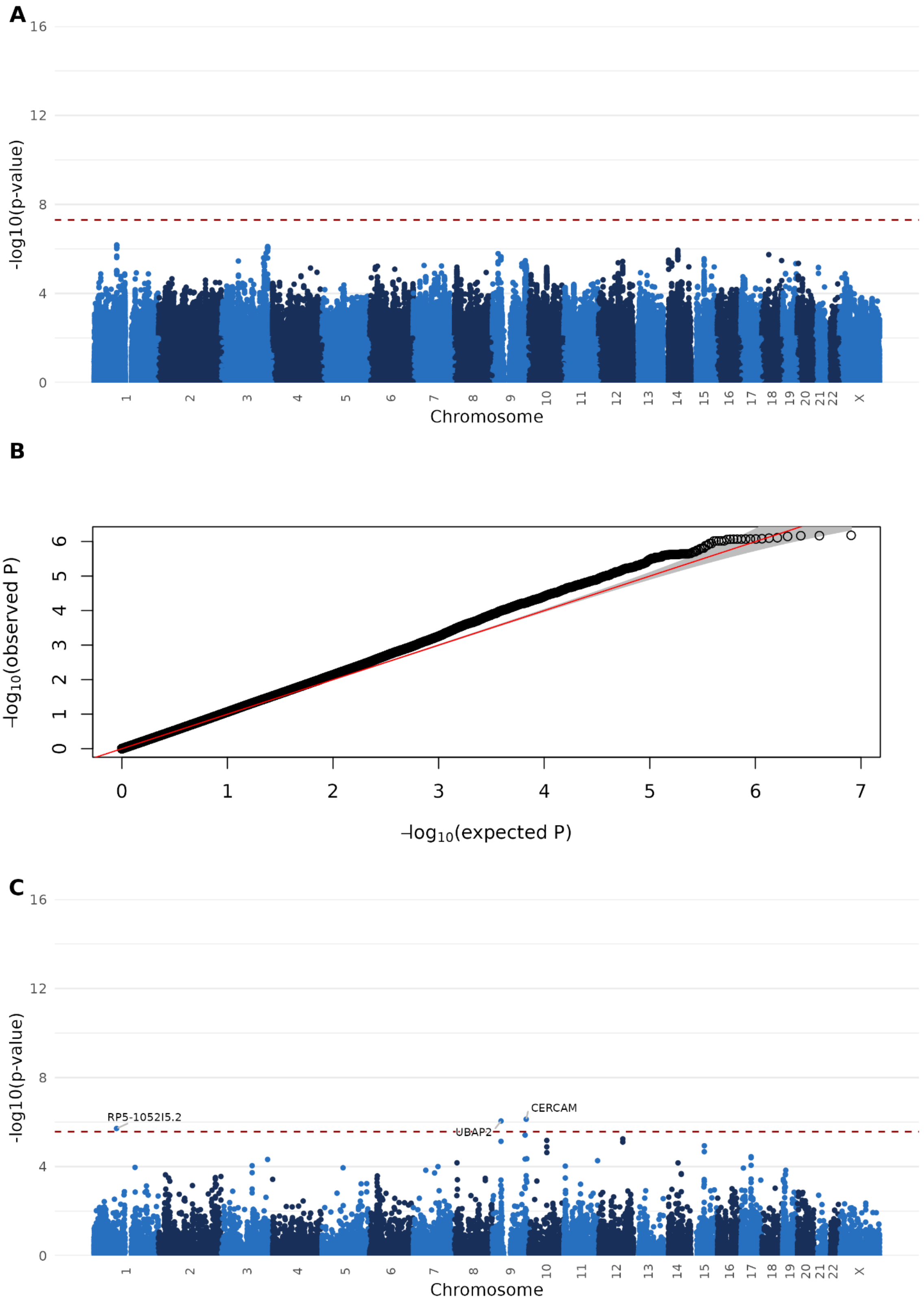

Figure S19.5. GWAS results for MDD-related Cluster 5 membership in FinnGen data (N = 277,252). (A) SNP-based genome-wide Manhattan plot. Association analyses were performed using linear regression to test the association between each SNP and the posterior log odds of cluster membership, controlling for age, sex, the first ten genetic principal components, and the genotyping array. In the plot, nominal p-values are displayed. The significance threshold (5×10^{-8}) is depicted with a dashed dark red line. (B) QQ-plot. (C) Gene-based genome-wide Manhattan plot. MAGMA gene-level analysis was performed to identify putative significant genes using a SNPwise-multi model, defining the SNP set of each gene with a ± 10 kb window. In the plot, nominal p-values are displayed. The statistically significant genes are indicated with labels. The significance threshold (2.7×10^{-6}) is depicted with a dashed dark red line.

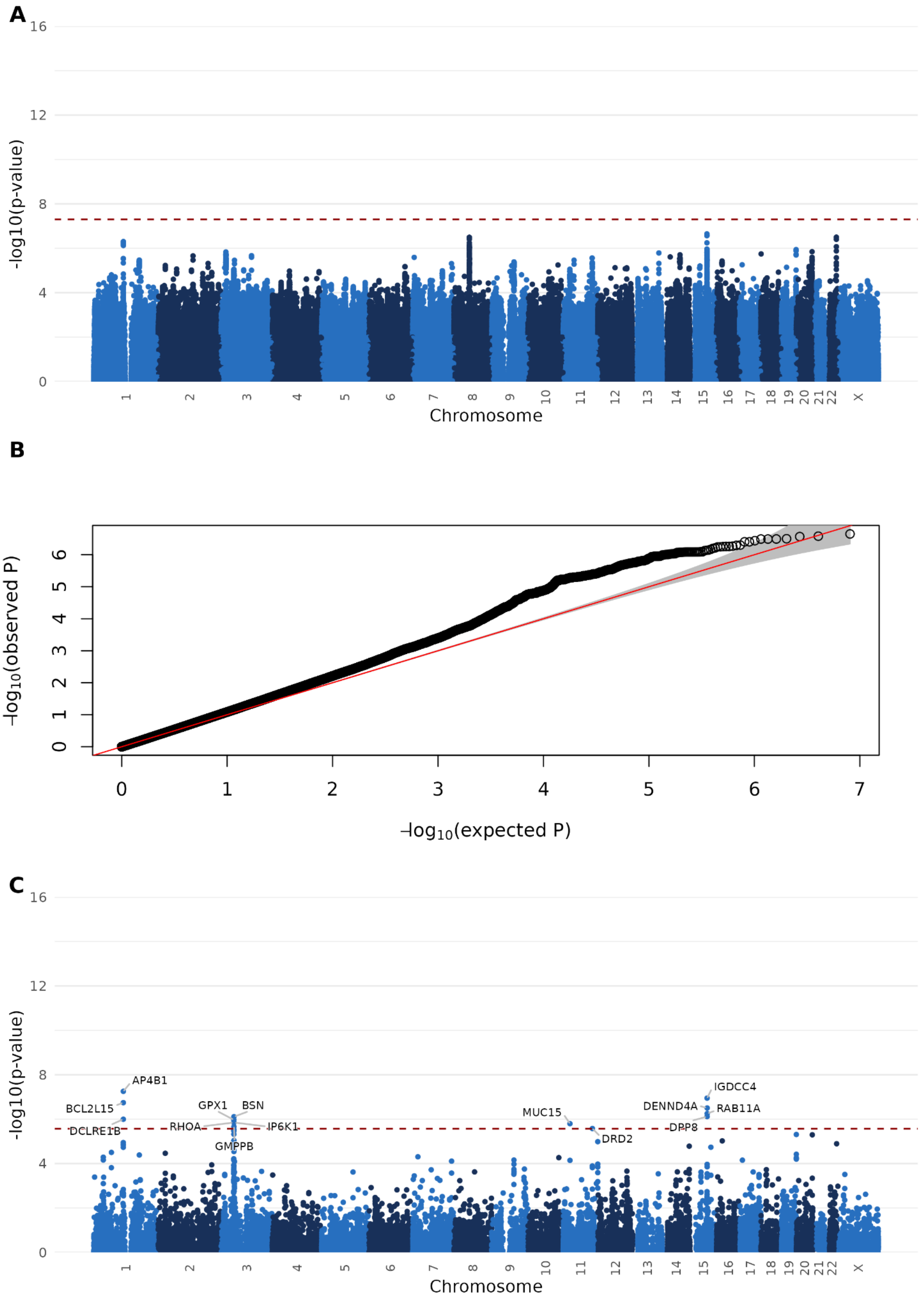

Figure S19.6. GWAS results for MDD-related Cluster 6 membership in FinnGen data (N = 277,252). (A) SNP-based genome-wide Manhattan plot. Association analyses were performed using linear regression to test the association between each SNP and the posterior log odds of cluster membership, controlling for age, sex, the first ten genetic principal components, and the genotyping array. In the plot, nominal p-values are displayed. The significance threshold (5×10^{-8}) is depicted with a dashed dark red line. (B) QQ-plot. (C) Gene-based genome-wide Manhattan plot. MAGMA gene-level analysis was performed to identify putative significant genes using a SNPwise-multi model, defining the SNP set of each gene with a ± 10 kb window. In the plot, nominal p-values are displayed. The statistically significant genes are indicated with labels. The significance threshold (2.7×10^{-6}) is depicted with a dashed dark red line.

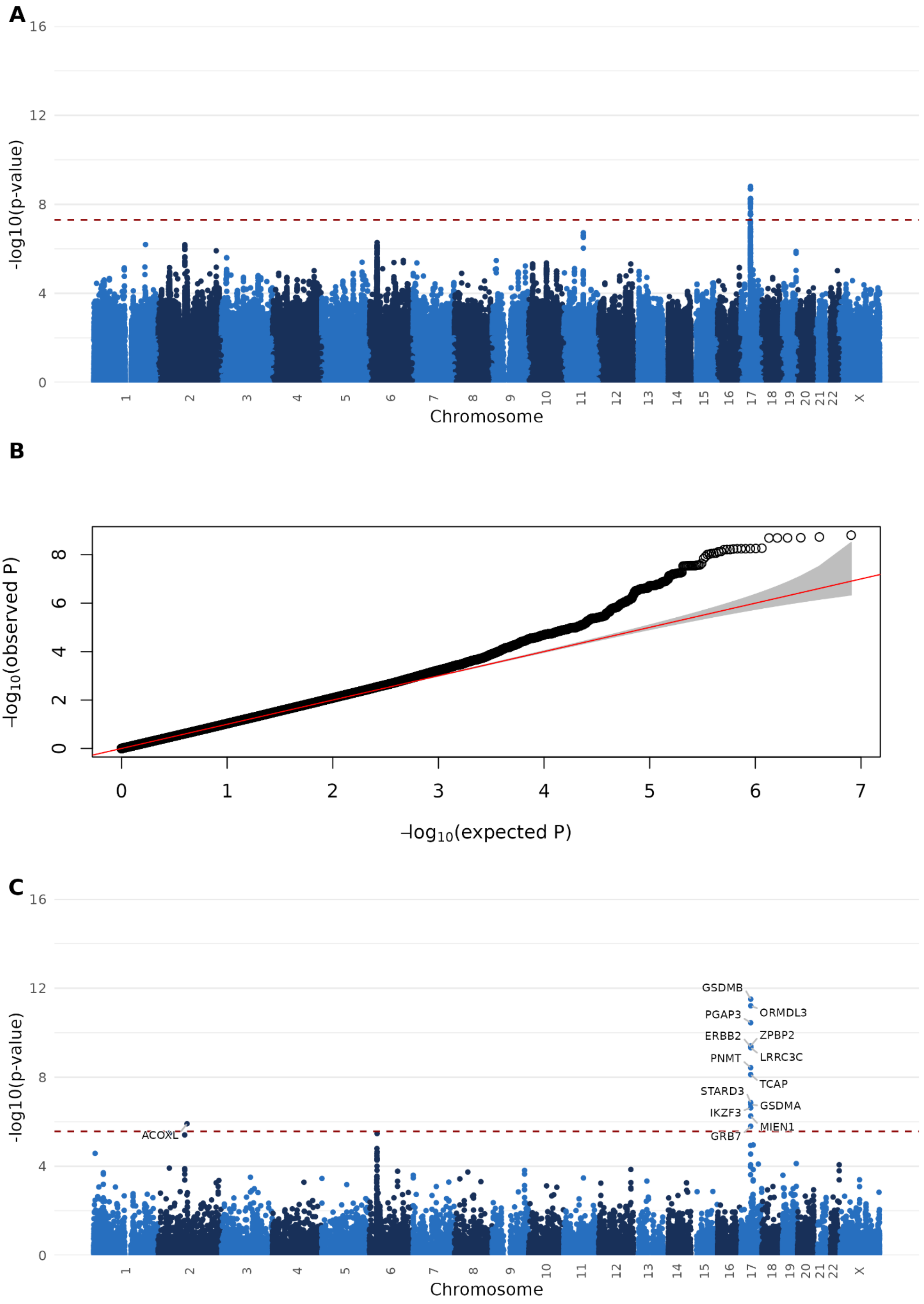

Figure S19.7. GWAS results for MDD-related Cluster 7 membership in FinnGen data (N = 277,252). (A) SNP-based genome-wide Manhattan plot. Association analyses were performed using linear regression to test the association between each SNP and the posterior log odds of cluster membership, controlling for age, sex, the first ten genetic principal components, and the genotyping array. In the plot, nominal p-values are displayed. The significance threshold (5×10^{-8}) is depicted with a dashed dark red line. (B) QQ-plot. (C) Gene-based genome-wide Manhattan plot. MAGMA gene-level analysis was performed to identify putative significant genes using a SNPwise-multi model, defining the SNP set of each gene with a ± 10 kb window. In the plot, nominal p-values are displayed. The statistically significant genes are indicated with labels. The significance threshold (2.7×10^{-6}) is depicted with a dashed dark red line.

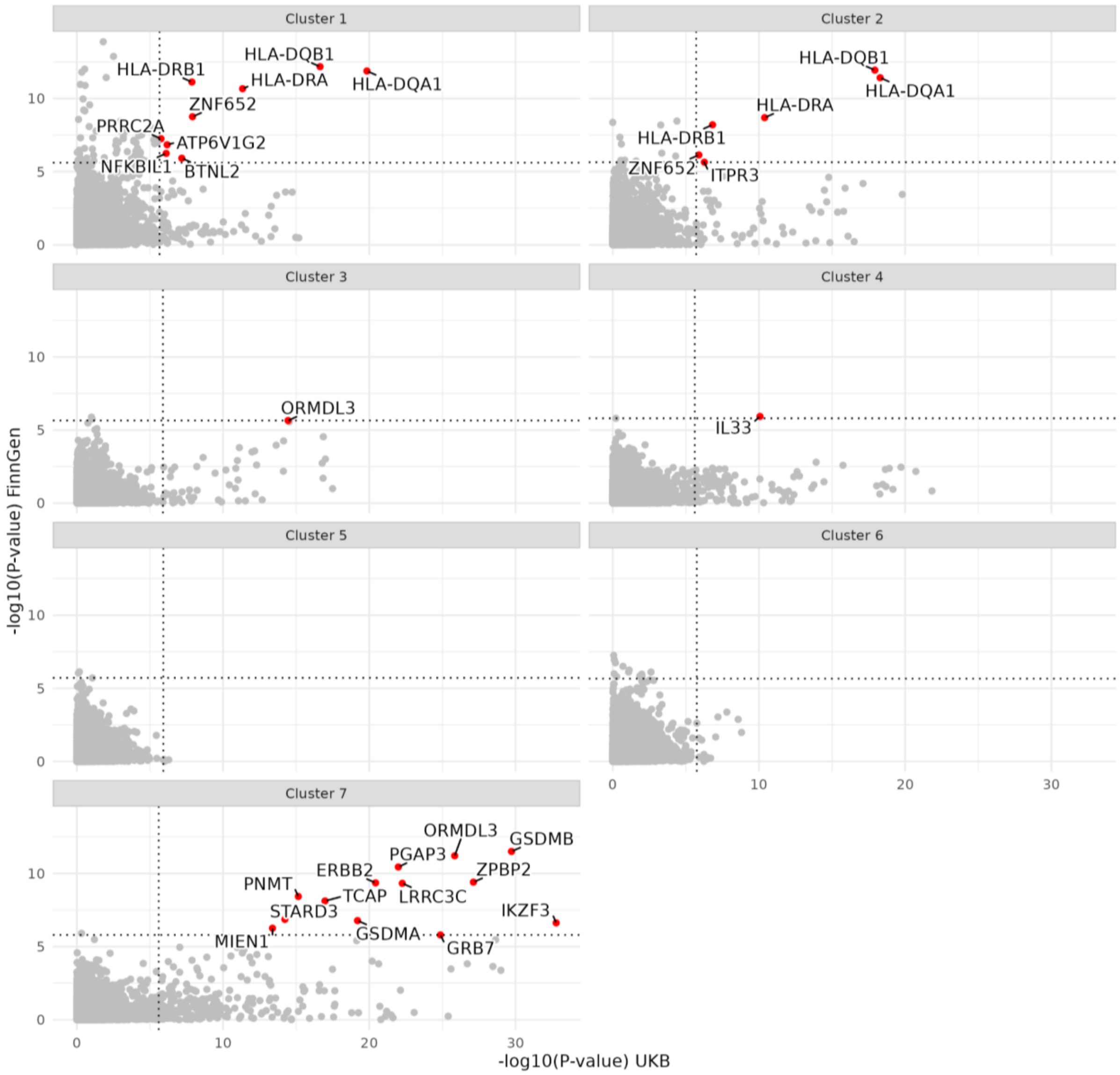

Figure S20. Scatter plot of gene-level aggregated (negative log-transformed) *p*-values in UKB (N = 249,167) versus FinnGen (277,252). Gene-level *p*-values were aggregated with MAGMA. Genes that are significant according to Holm's method are indicated with red dots and shown with labels.

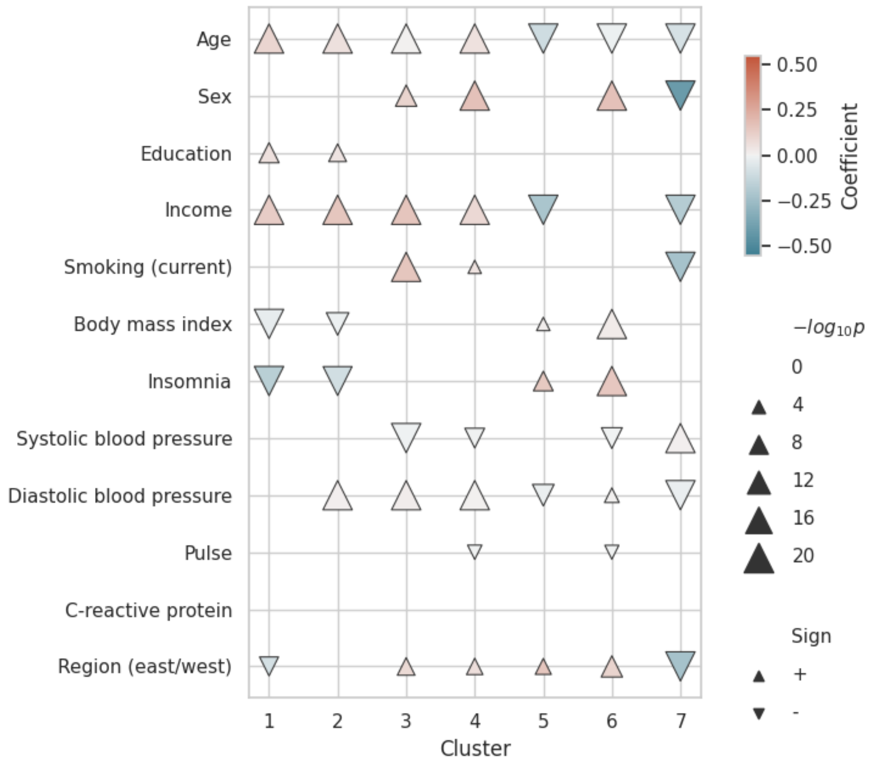

Figure S21. Complex linear regression models involving available non-genetic factors included in the THL data for each cluster (N = 23,786). The "complex regression model" uses all available risk factors at once in a single model. The posterior log odds of being in a given cluster serves as the target variable. The direction of the triangles corresponds to the sign of the coefficient (positive - upwards; negative - downwards) and the color details the magnitude. The size of the triangles is proportional to the -log10 *p*-value, and only nominally significant values are shown (-log10 *p* > 4). CRP: C-reactive protein; bp: blood pressure; sex: 1-males, 2-females.

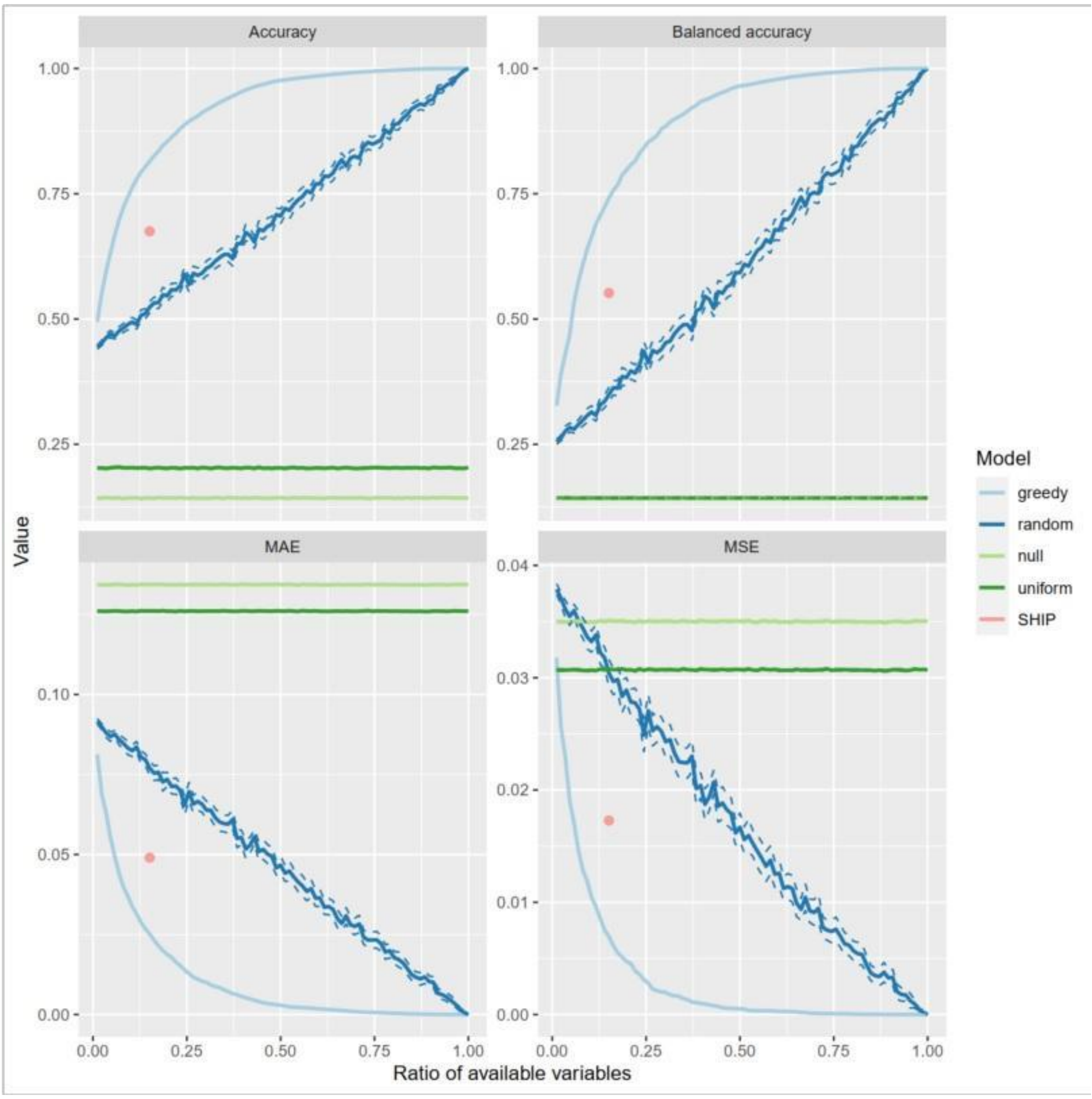

Figure S22. Four measures showing the performance of the clustering method using only a fraction of all consensual disease variables from UKB. The green lines show various baseline null models (a totally random (null) and a uniform cluster assignment). The dark blue line shows how a randomly chosen variable set performs, and the light blue curve shows how a greedily selected (~optimal) variable set performs. The red dot indicates the performance of the SHIP variable set. See methods for details. MAE: Mean absolute error, MSE: Mean squared error.

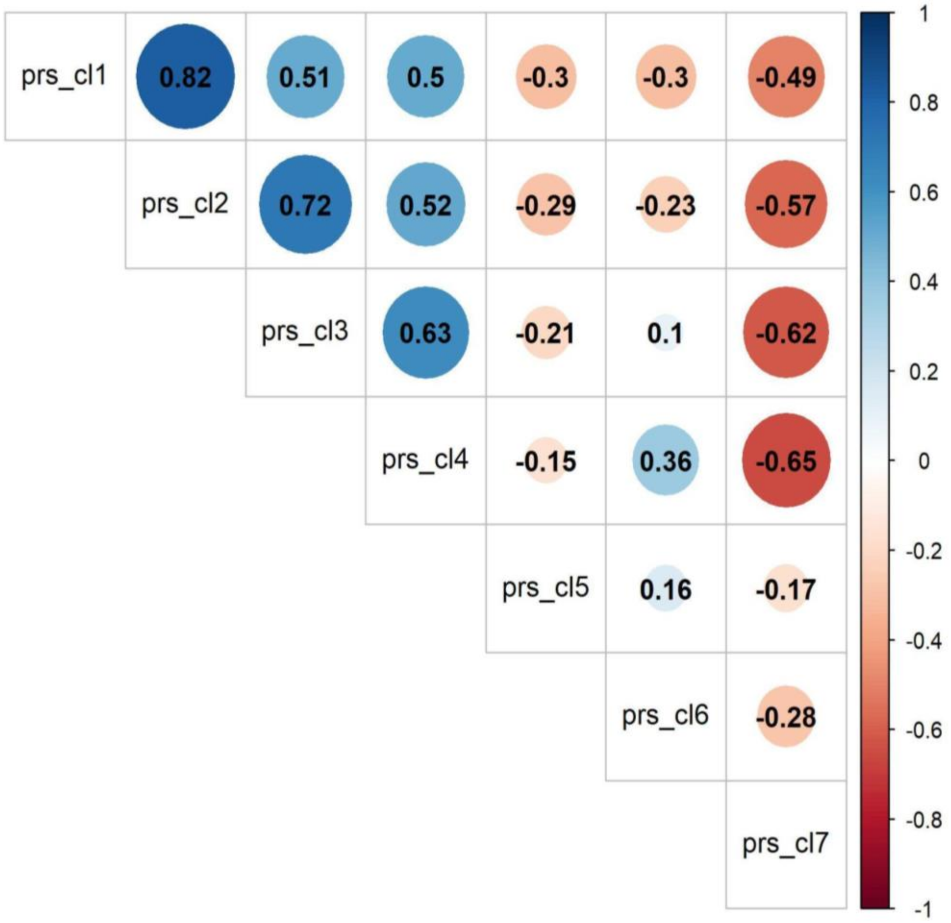

Figure S23. Pearson correlation matrix for polygenic risk scores (PRS) in SHIP (N = 1108). PRS were calculated as described in the online methods and based on MDD-related cluster GWAS from UKB. Correlation between the PRS revealed the same pattern on the genetic level as has been observed in the other cohorts.

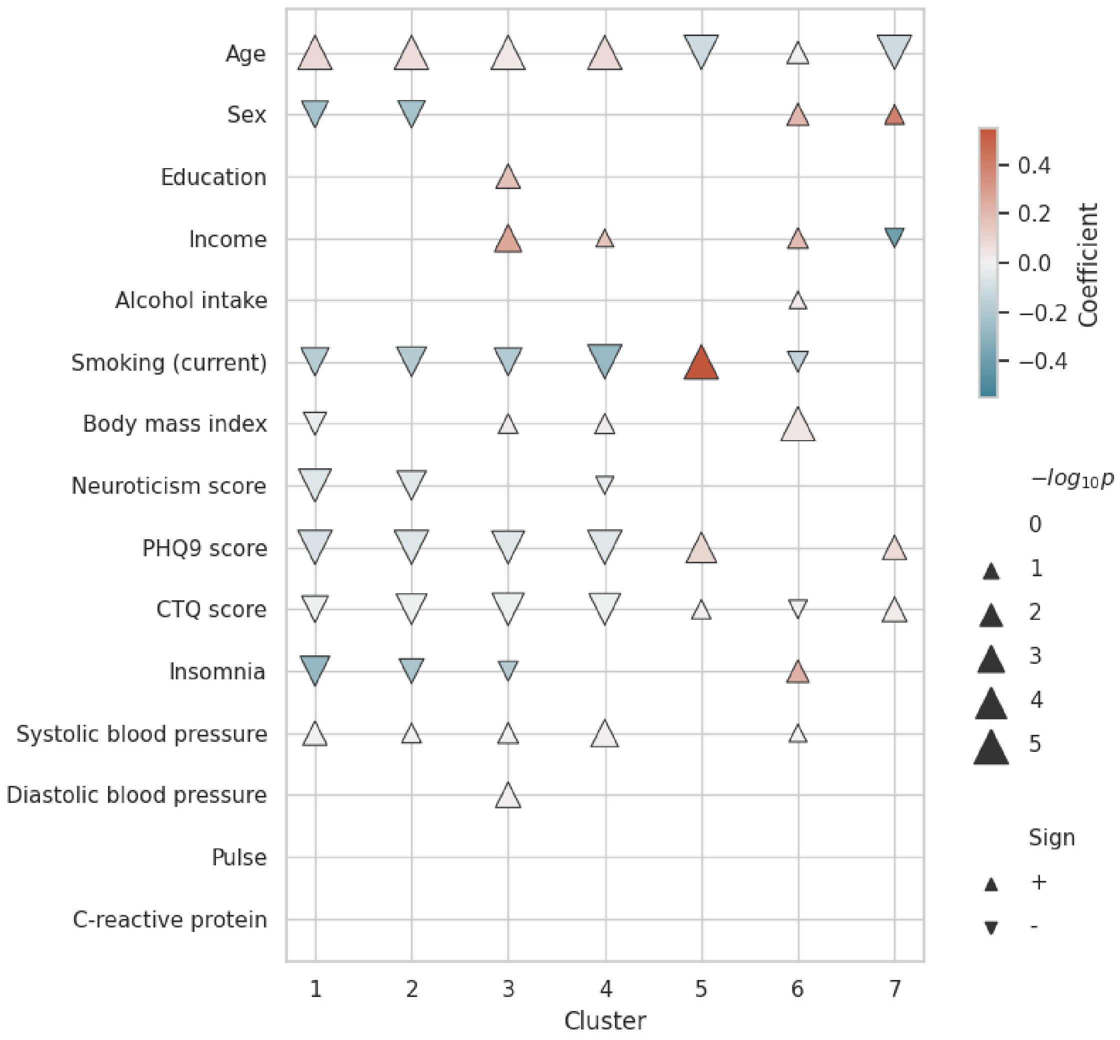

Figure S24. Simple linear regression models involving non-genetic risk factors included in the SHIP dataset for each cluster (N = 1126). The simple linear regression model involves one factor at a time with age and sex as covariates for each cluster. The posterior log odds of being in a given cluster serves as the target variable. The direction of the triangles corresponds to the sign of the coefficient (positive - upwards; negative - downwards) and the color details the magnitude. The size of the triangles is proportional to the -log10 *p*-value, and only values *p* > 0.05 are shown (-log10 *p* > 1.3). CTQ: childhood trauma questionnaire; sex: 1-males, 2-females.

Supplementary Methods

Characterization of the weighted directed MDD-related multimorbidity score

The scores used for clustering are based on the *probability of strong relevance* of each disease with respect to MDD at specific time intervals. The concept of strong relevance has been characterized in previous studies (Lautner-Csorba et al. 2012, Ungvári et al. 2012, Lautner-Csorba et al. 2013, Antal et al. 2014, Marx et al. 2017). We have utilized this measure as a means to reflect direct (non-mediated) relationships between variables more accurately. Moreover, earlier research demonstrated that Bayesian filtering of pairwise comorbidity associations, focusing on strong relevance rather than merely pairwise associations, significantly enhanced the shared molecular background between diseases (Marx et al. 2017).

The posterior probability of strong relevance is then utilized as a weight in a cumulative sum, where a disease's onset (incidence) at a certain time interval is weighted by its probability of strong relevance during that interval. This sum represents a participant's *weighted MDD-related multimorbidity burden*, essentially indicating the expected count of MDD-related comorbid diseases at a given time interval.

To elucidate the relationship between the scores used for clustering and the incidence of MDD, we visualized the score distributions among participants in UKB, distinguishing between those diagnosed with MDD and those not diagnosed, across each time interval (see Figure 1A). Notably, the score distributions were consistently and statistically significantly elevated in the patient group diagnosed with MDD compared to those without MDD diagnosis. Additionally, we conducted logistic regression analyses for each time interval, using the MDD diagnosis as the dependent variable and the interval-specific score, along with covariates such as age, sex, income, and standardized birth year, as predictors (see Figure 1B). The results uniformly indicated a statistically significant and robust positive effect of the scores on MDD occurrence in every time interval. These findings affirm that the weighted direct MDD-related multimorbidity scores employed in the clustering have a substantial impact on the likelihood of MDD occurrence. This significance holds true even when accounting for the chronological year of diagnosis, as evidenced by including the subjects' standardized birth years in the logistic regression models.

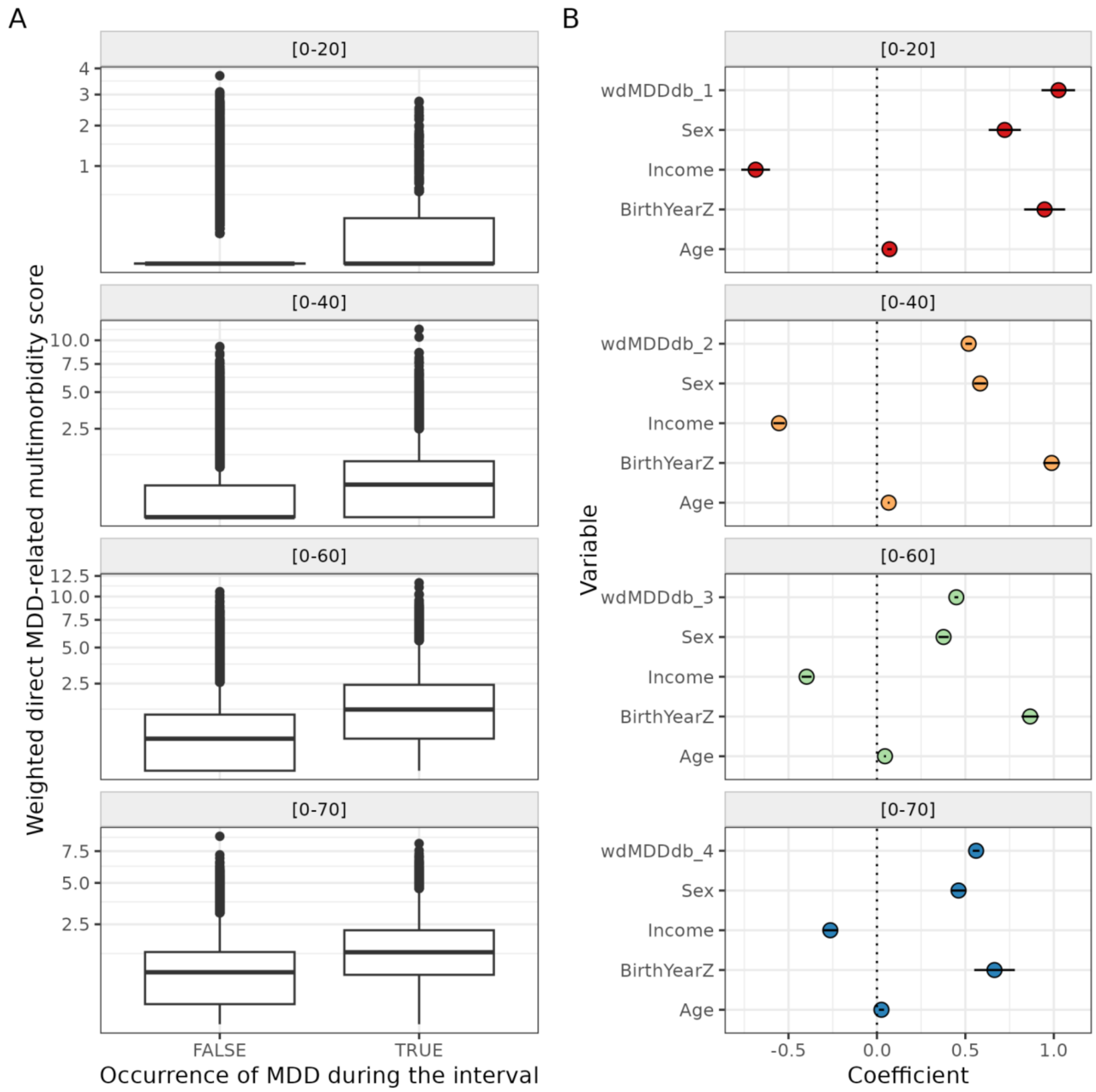

Figure SM1. Characterization of the weighted directed MDD-related multimorbidity score in the UKB (N=502,504). (A) Distribution of the weighted directed MDD-related multimorbidity score among individuals diagnosed and not diagnosed with MDD. (B) Results of logistic regression analyses for each time interval, using the MDD diagnosis as the dependent variable and the interval-specific score (with a prefix of "wdMDDdb"), along with covariates (age, sex, income, and standardized birth year) as predictors. 95% confidence intervals of the coefficient are indicated with lines. Colours indicate different time intervals.

To further explore the relationship between the age of MDD onset and the scores used for clustering, we generated two-dimensional density plots based on the subset of patients diagnosed with MDD (see Figure 2). These plots juxtapose the age at MDD onset against the values of the weighted direct MDD-related multimorbidity scores at various time intervals. The plots reveal a clear age-related trend: higher scores at earlier intervals (e.g., [0-20 years]) are associated with an earlier onset of MDD, whereas higher scores at later intervals (e.g., [0-70

years]) correspond to a later onset of MDD. This observation suggests that the scores also effectively encapsulate information about the age of MDD onset.

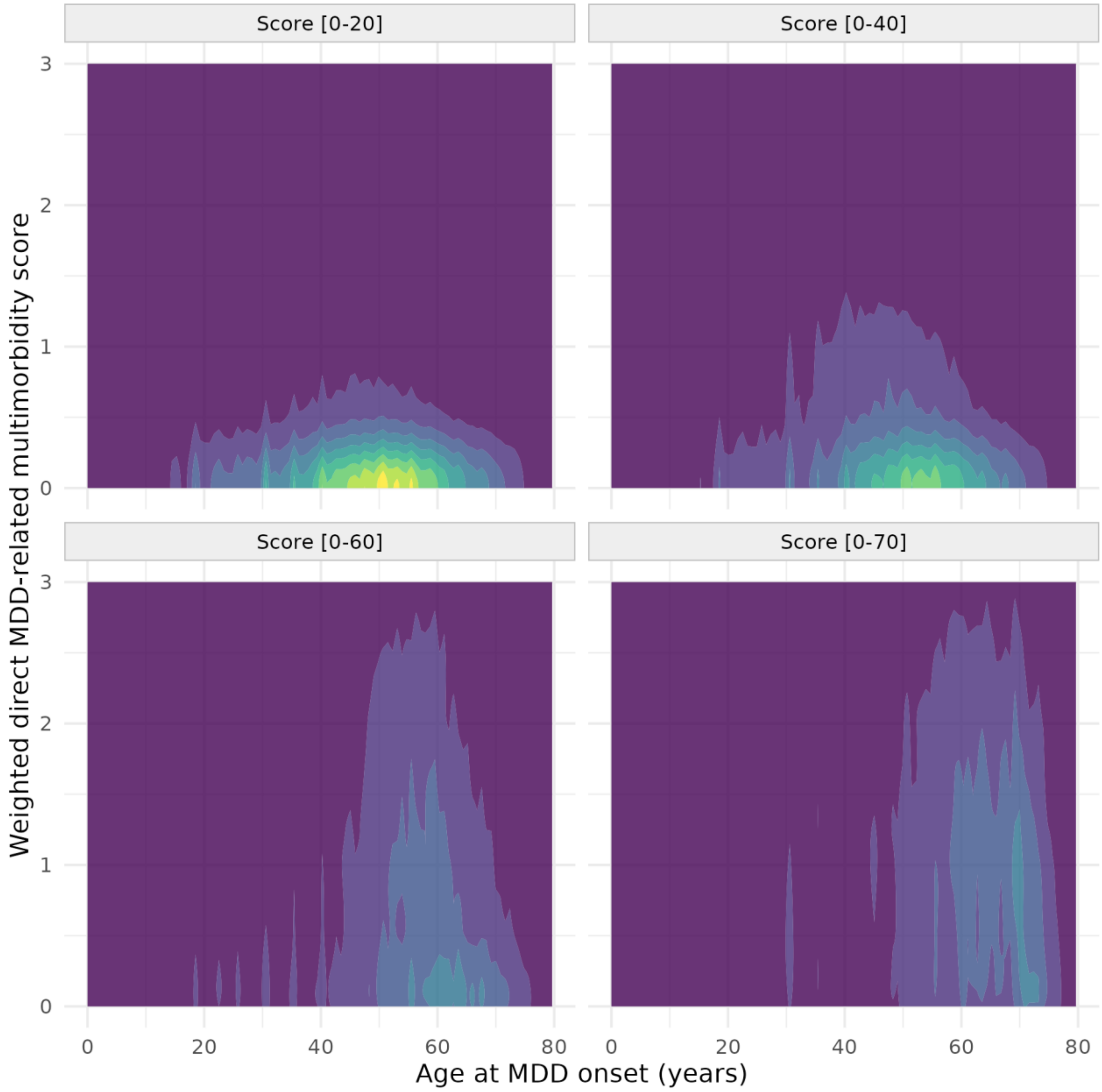

Figure SM2. The relationship between the age of MDD onset and the weighted direct MDD-related multimorbidity score in the UKB (N=53,473). Each box shows a two-dimensional density plot of patients diagnosed with MDD corresponding to a certain time interval, where the x-axis represents the age at MDD onset, and the y-axis corresponds to the weighted directed MDD-related multimorbidity score.

Determination of the optimal number of clusters

Based on the cross-cohort relevance scores, we computed the *weighted direct MDD-related multimorbidity scores* for each participant in each cohort and for each cumulative time interval (see Online Methods). The score for the i-th participant in the t-th time interval is denoted as:

$$multimorbidity\text{-}score^{(t)}(i)$$

These weighted direct MDD-related multimorbidity scores reflect the temporal MDD-related multimorbidity burden of a participant. These scores define a 4-dimensional space of the participants. More specifically, we used the raw score for the first time interval and the difference between the consecutive scores for the subsequent time intervals, i.e.,

$$d^{(1)}(i) = multimorbidity\text{-}score^{(1)}(i)$$
$$d^{(2)}(i) = multimorbidity\text{-}score^{(2)}(i) - multimorbidity\text{-}score^{(1)}(i)$$
$$d^{(3)}(i) = multimorbidity\text{-}score^{(3)}(i) - multimorbidity\text{-}score^{(2)}(i)$$
$$d^{(4)}(i) = multimorbidity\text{-}score^{(4)}(i) - multimorbidity\text{-}score^{(3)}(i)$$

Together, $D(i) = \{d^{(i)}; i = 1..4\}$ define a 4-dimensional vector for the i-th participant.

We clustered all participants whose observed multimorbidity scores were complete (25% of the participants) using the k-means clustering algorithm in this 4-dimensional space. In the case of younger participants, one or more multimorbidity scores were unavailable because there were no observations of their future disease onsets. We assigned them to the nearest cluster (of which cluster center was nearest).

The determination of the optimal number of clusters in our study was conducted through a systematic manual process, employing multiple analytical techniques to ensure accuracy and reliability. During this process, we computed the Adjusted Rand index between consecutive cluster numbers, performed a principal component analysis of participants and disease over- and underrepresentation analysis, compared weighted disease burden among clusters, and computed Silhouette scores of the resulting clusters. The multimorbidity burden is calculated for each individual by summing the relevance score for each diagnosed disease multiplied by the number of years spent in that disease.

More specifically, we followed a heuristic approach: with an increasing number of clusters, (1) we inspected the change in cluster structure by visualizing the switching of individuals between clusters while increasing the number of clusters, (2) we calculated disease risk profiles in the clusters, and (3) assessed the quality of the actual number of clusters based on the other metrics and analyses.

Finally, our investigation led us to select 7 clusters as the optimal solution. The transitions from 5 to 6 clusters and from 6 to 7 clusters demonstrated significant changes in cluster structure,

indicating meaningful distinctions between participant groups. However, beyond 7 clusters, the observed changes were insignificant from all viewpoints, which is also reflected by the high Adjusted Rand indices between 7 and 8 clusters (Adj. Rand index = 0.967) and between 8 and 9 (Adj. Rand index = 0.936). Besides, when using 8 clusters, the smallest cluster consisted of only 1.6% of all participants. This highlights the limitations of using a higher number of clusters, as it would lead to exceedingly small clusters that may not provide meaningful insights or statistical robustness.

In the following, we demonstrate the heuristic process of finding the optimal number of clusters through various types of figures (see Supplementary Methods Figures SM3-SM7).

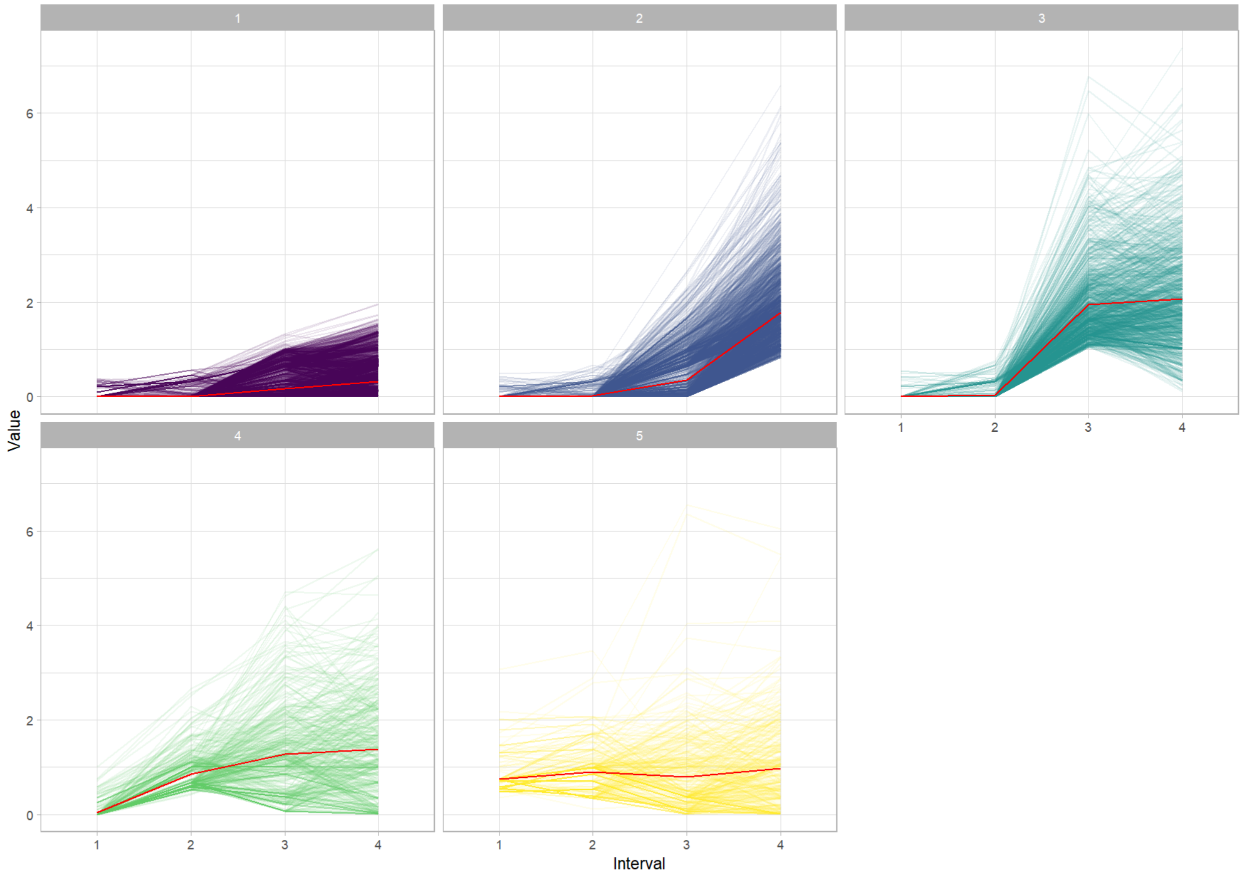

Figure SM3.1. Trajectories of the weighted direct MDD-related multimorbidity score over time using 5 clusters in the discovery cohorts (UKB, N = 502,504; CHSS, N = 645,913; THL, N = 41,092). Each box corresponds to a cluster of participants in which the trajectories of the scores are similar. Each colored line corresponds to the trajectory of a single individual. The red lines show the mean trajectory in a cluster. The x-axis corresponds to the discrete cumulative time intervals (1: 0-20, 2: 0-40, 3: 0-60, and 4: 0-70), and the y-axis shows the value of the weighted direct MDD-related multimorbidity score.

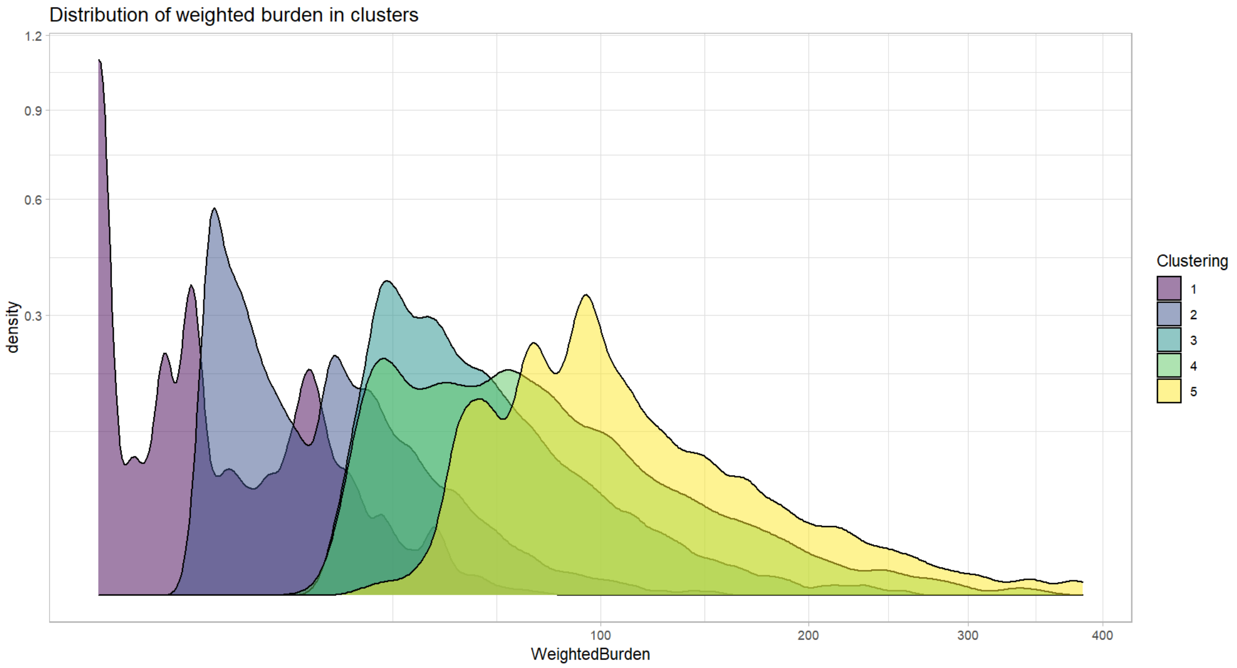

Figure SM3.2. Distribution of weighted direct MDD-related multimorbidity burden in the clusters where the number of clusters is set to 5 in the discovery cohorts (UKB, N = 502,504; CHSS, N = 645,913; THL, N = 41,092). The x-axis corresponds to the weighted disease burden, and the y-axis shows the density of the distribution. Clusters 1 and 2 show multimodal distributions.

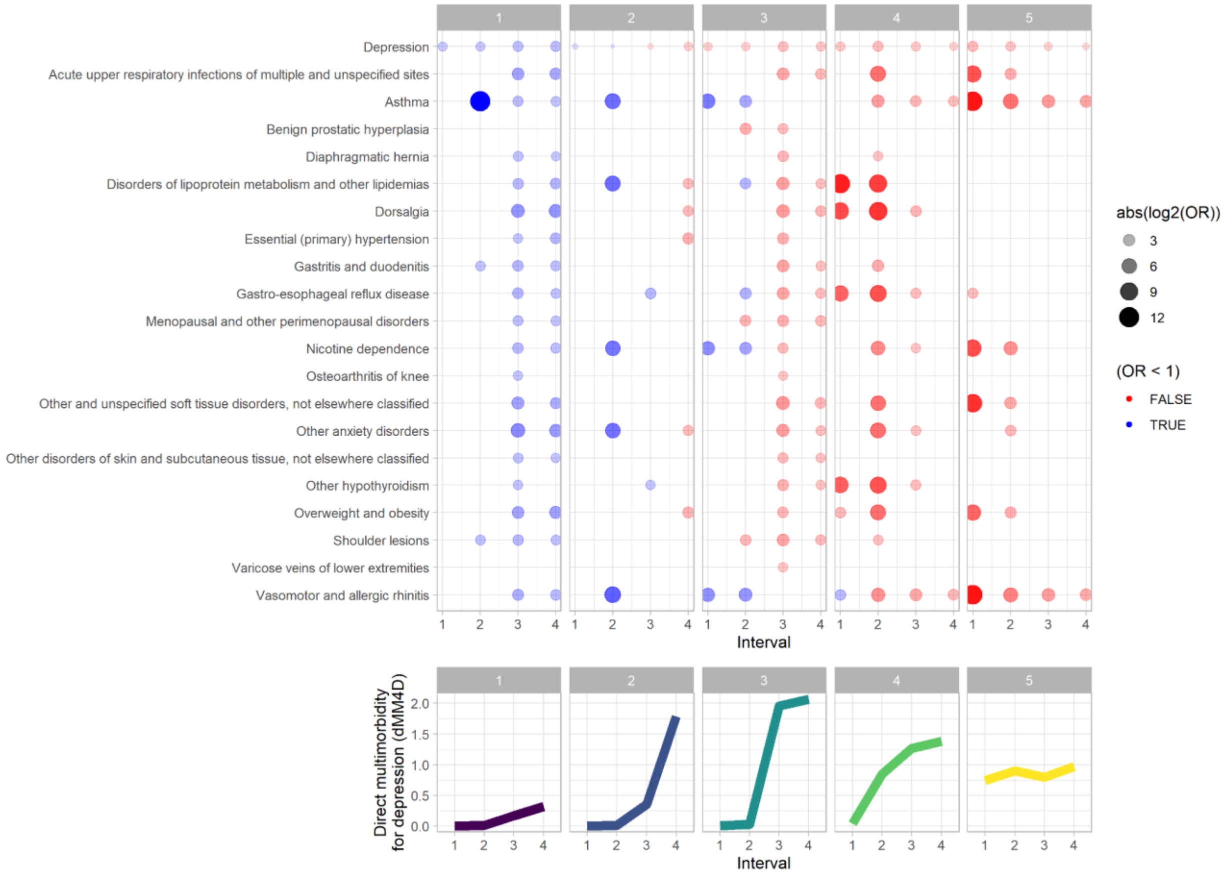

Figure SM3.3. The top over- and underrepresented diseases in each discrete time interval in the clusters where the number of clusters is set to 5 in the discovery cohorts (UKB, N = 502,504; CHSS, N = 645,913; THL, N =

41,092). In the upper panel, the colored dots show the odds ratio of a disease in a specific time interval (indicated by the columns) for the individuals belonging to a given cluster (indicated by the boxes). Fisher's exact test was used to assess the statistical significance of the association between the disease status and the cluster membership. The color and the size of the dots show the direction and the size of the effect (blue: decreased risk, red: increased risk), respectively. For each visible dot, the Bonferroni-adjusted p-value < 0.05, the OR > 3, or OR < ⅓, and the disease prevalence in the time interval exceeds 0.05. Otherwise, the dot is not shown. The lower panel shows the mean trajectory of the weighted direct MDD-related multimorbidity score in the clusters.

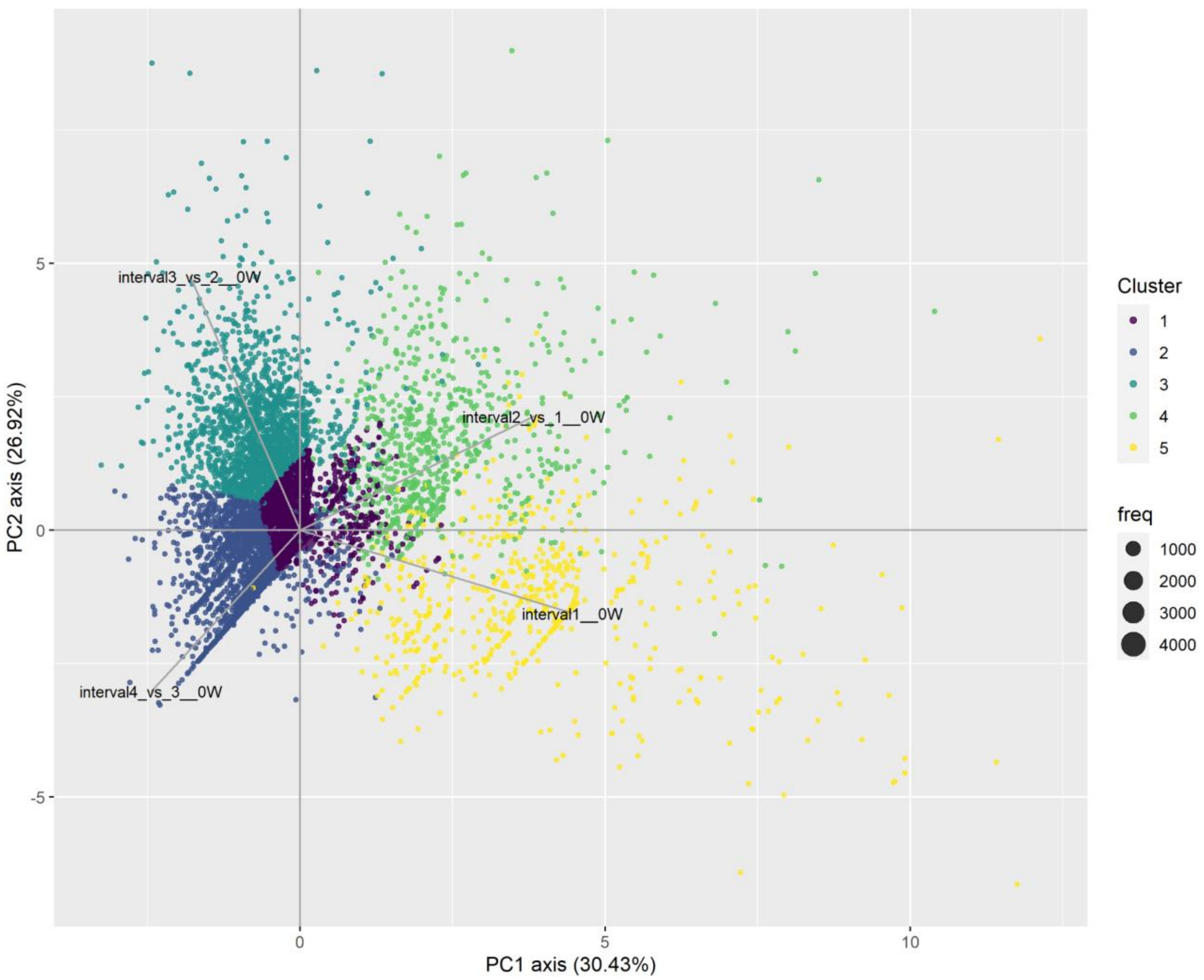

Figure SM3.4. The first two principal components of the 4-dimensional space defined by the temporal MDD-related multimorbidity burden score of the participants where the number of clusters is set to 5 in the discovery cohorts (UKB, N = 502,504; CHSS, N = 645,913; THL, N = 41,092). Dots correspond to individuals, where the size of the dots indicates the number of individuals in that specific region of space. The labeled loading vectors show the strength and direction of the association between each variable in the 4-dimensional space and each principal component.

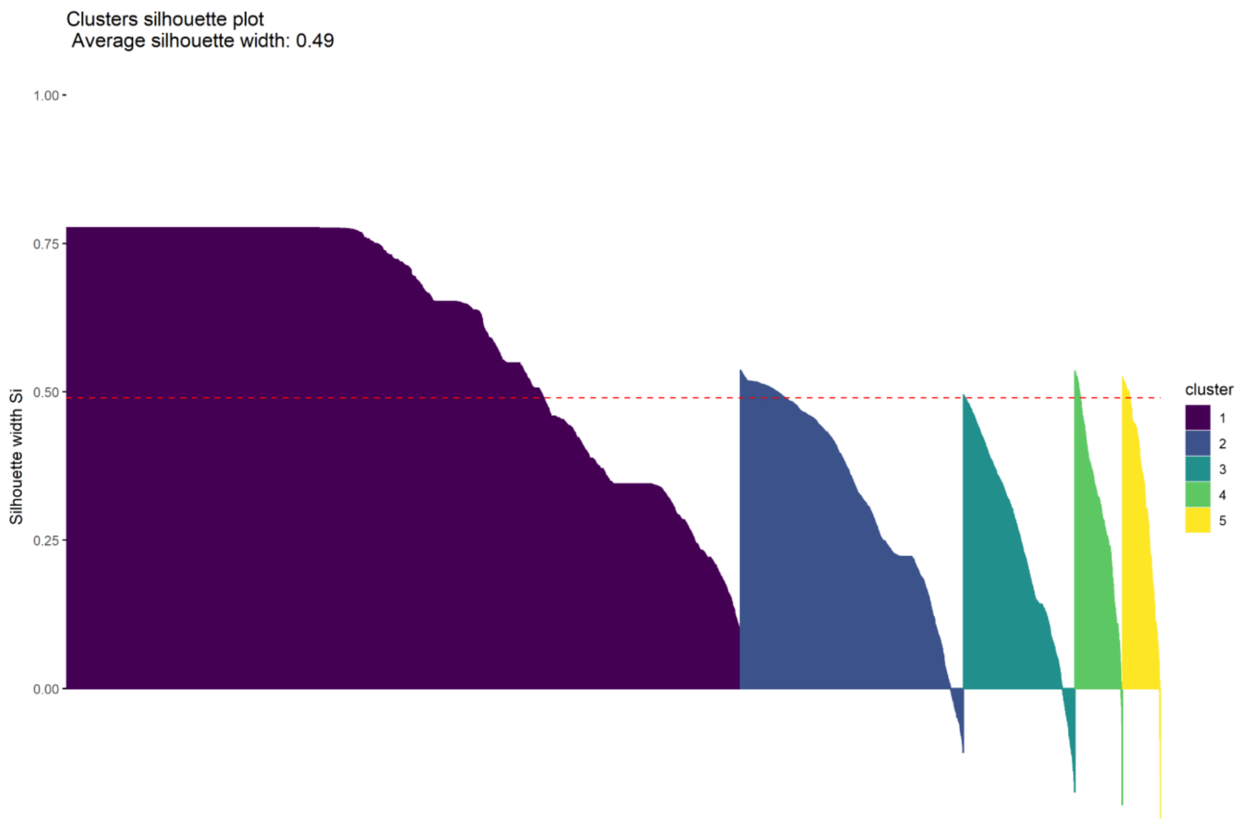

Figure SM3.5. Silhouette plot of the clusters where the number of clusters is set to 5 in the discovery cohorts (UKB, N = 502,504; CHSS, N = 645,913; THL, N = 41,092). Each individual is represented by a vertical line, with its position indicating the cluster it belongs to and its length representing the Silhouette coefficient for that individual.

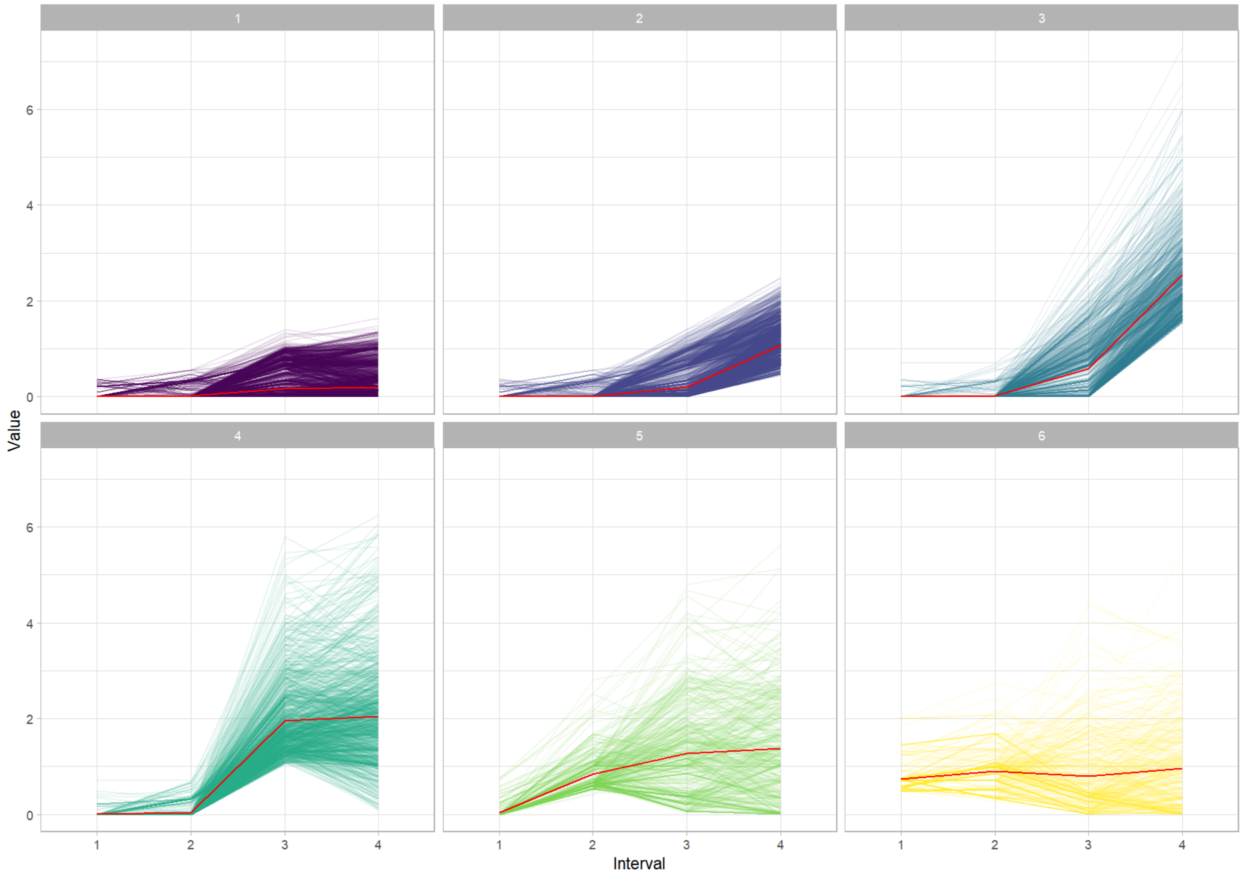

Figure SM4.1. Trajectories of the weighted direct MDD-related multimorbidity score over time using 6 clusters in the discovery cohorts (UKB, N = 502,504; CHSS, N = 645,913; THL, N = 41,092). See the caption of Figure SM3.1 for details.

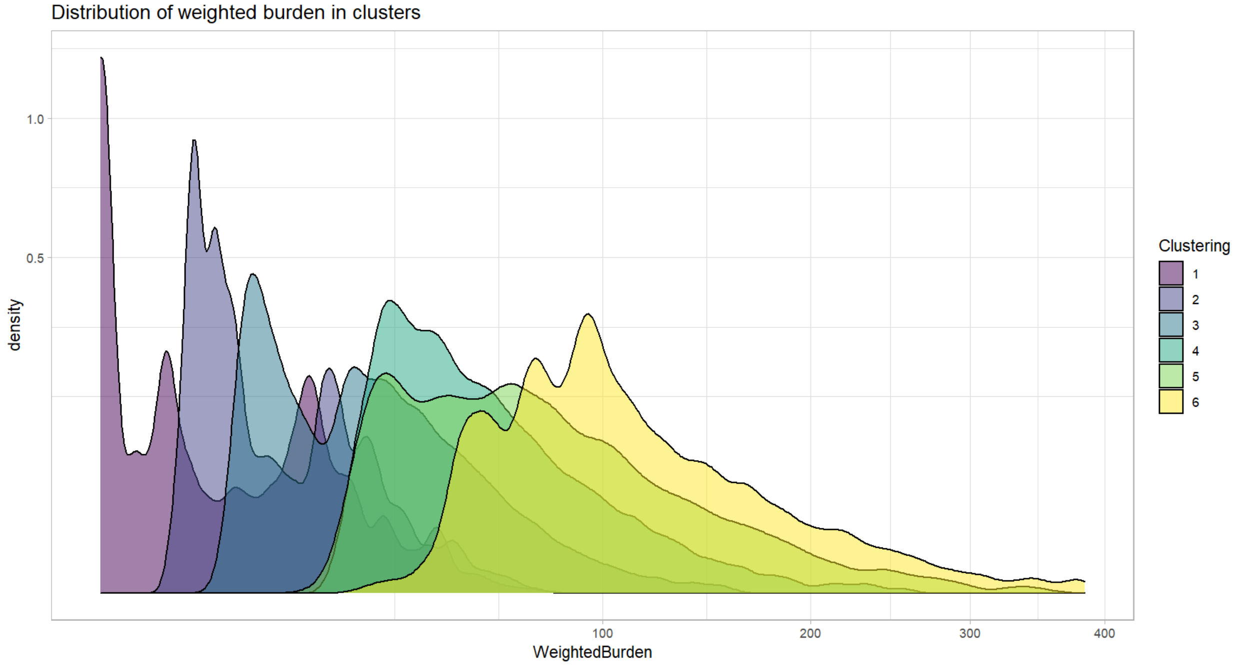

Figure SM4.2. Distribution of weighted direct MDD-related multimorbidity burden in the clusters where the number of clusters is set to 6 in the discovery cohorts (UKB, N = 502,504; CHSS, N = 645,913; THL, N = 41,092). See the caption of Figure SM3.2 for details.

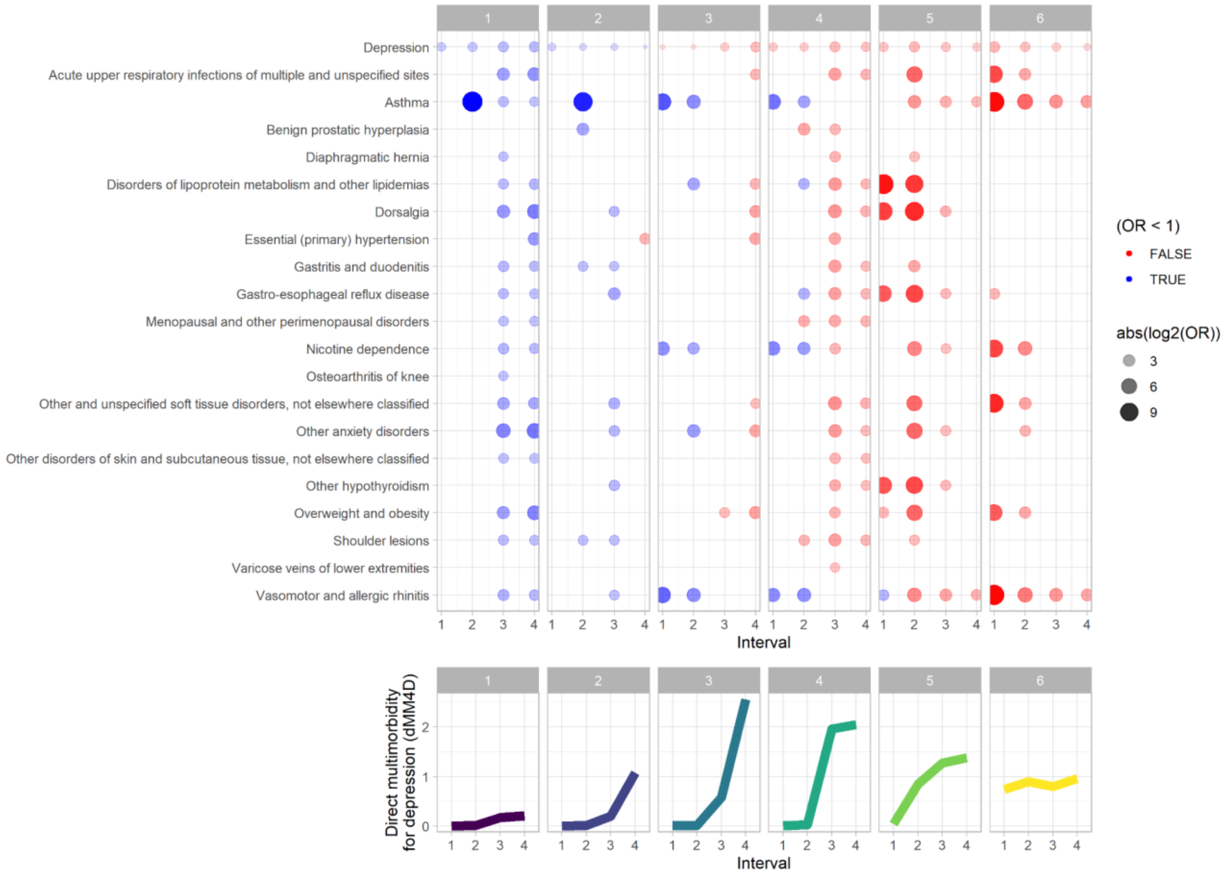

Figure SM4.3. The top over- and underrepresented diseases in each discrete time interval in the clusters where the number of clusters is set to 6 in the discovery cohorts (UKB, N = 502,504; CHSS, N = 645,913; THL, N = 41,092). See the caption of Figure SM3.3 for details.

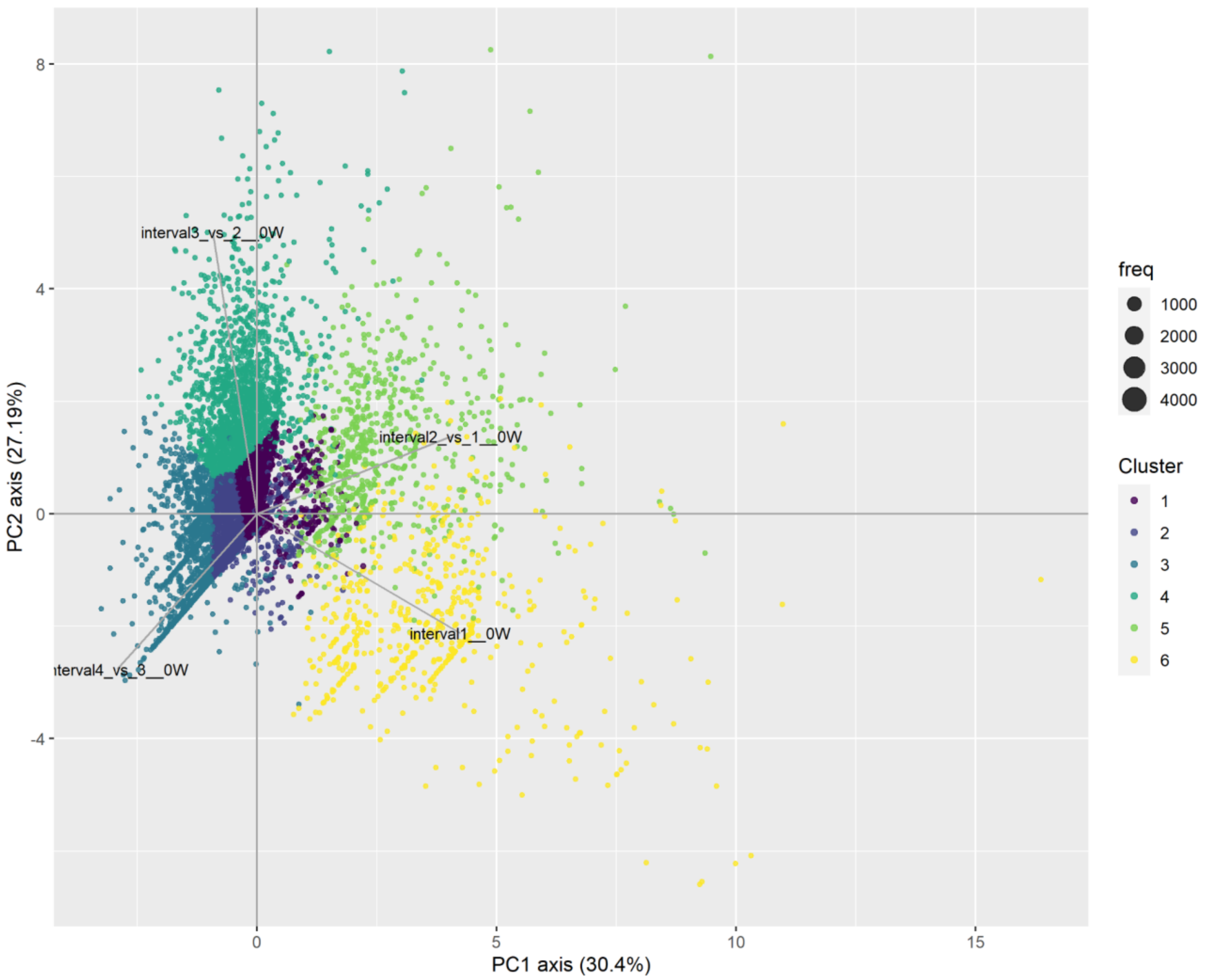

Figure SM4.4. The first two principal components of the 4-dimensional space defined by the temporal MDD-related multimorbidity burden score of the participants where the number of clusters is set to 6 in the discovery cohorts (UKB, N = 502,504; CHSS, N = 645,913; THL, N = 41,092). See the caption of Figure SM3.4 for details.

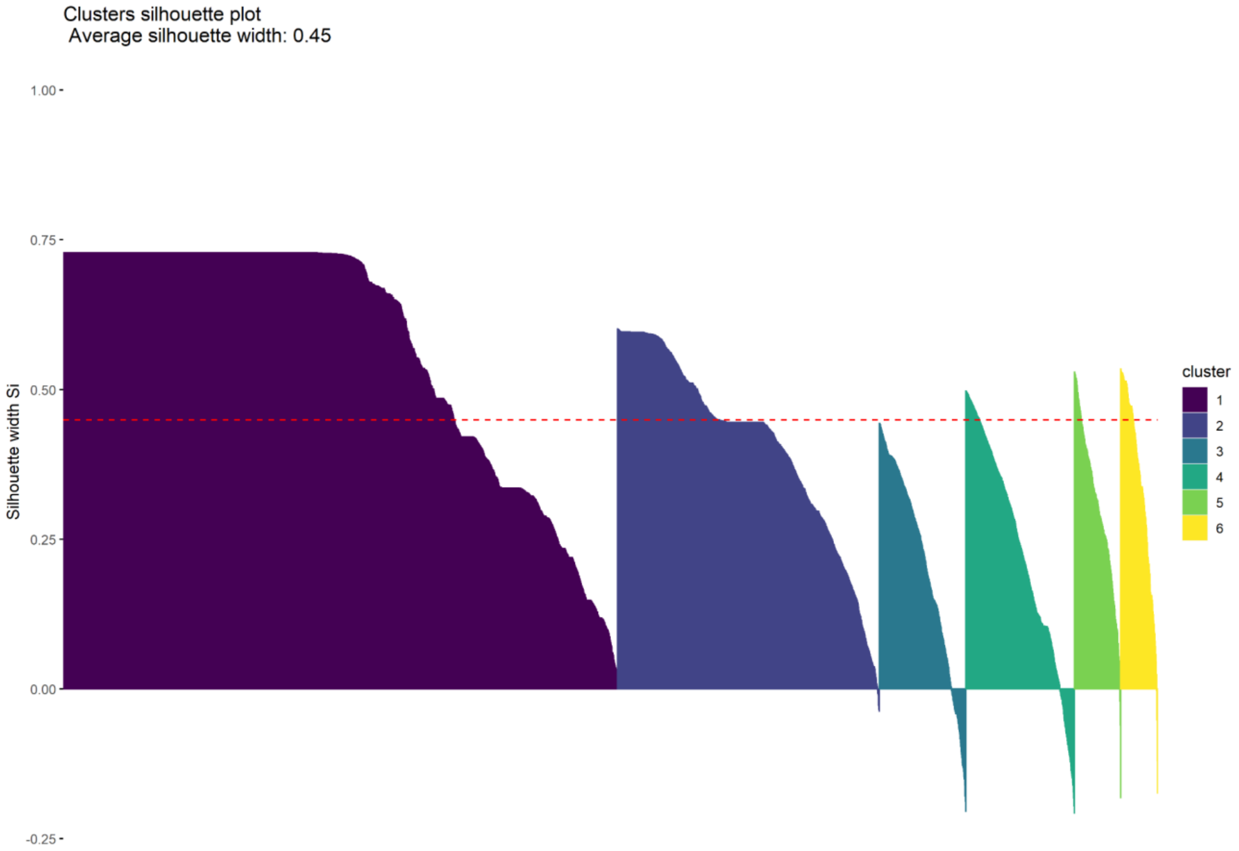

Figure SM4.5. Silhouette plot of the clusters where the number of clusters is set to 6 in the discovery cohorts (UKB, N = 502,504; CHSS, N = 645,913; THL, N = 41,092). See the caption of Figure SM3.5 for details.

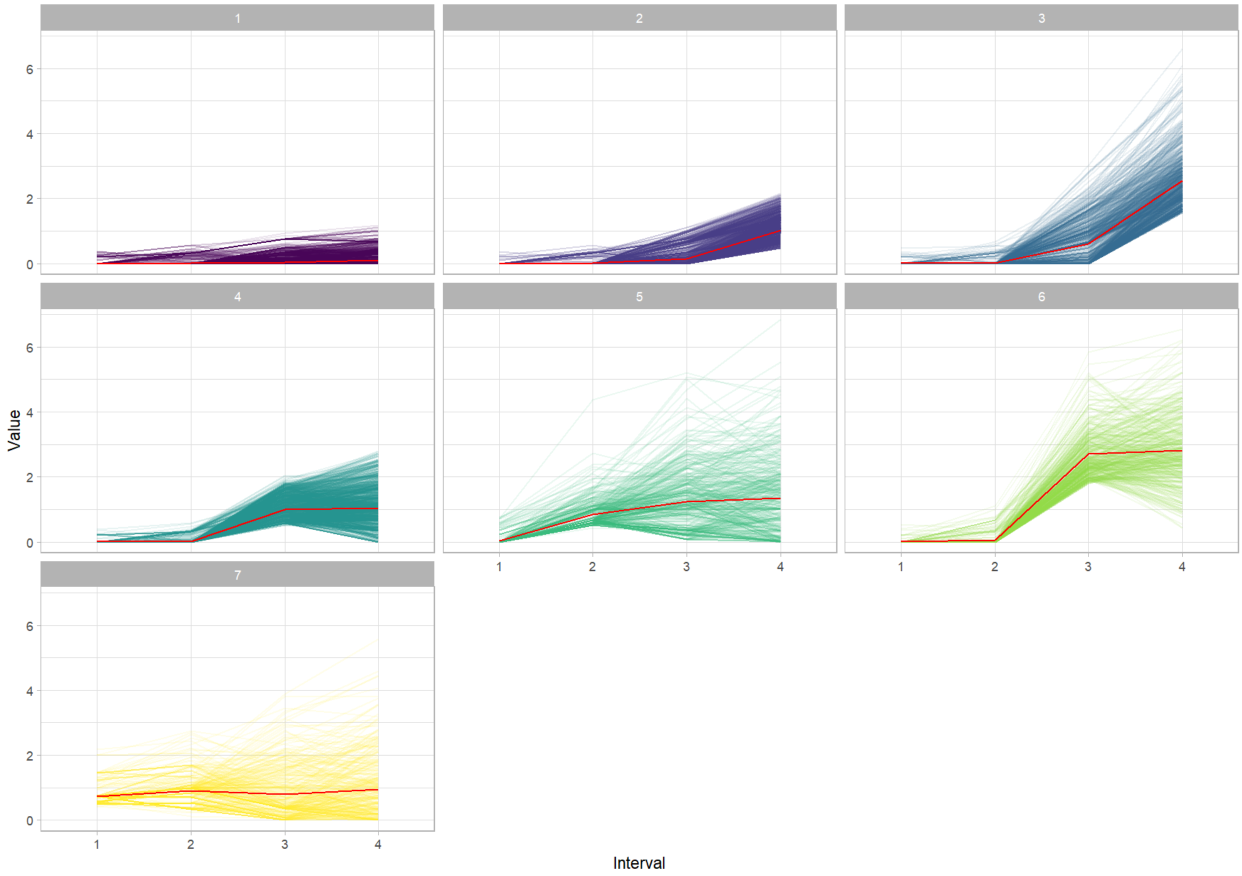

Figure SM5.1. Trajectories of the weighted direct MDD-related multimorbidity score over time using 7 clusters

in the discovery cohorts (UKB, N = 502,504; CHSS, N = 645,913; THL, N = 41,092). See the caption of Figure SM3.1 for details.

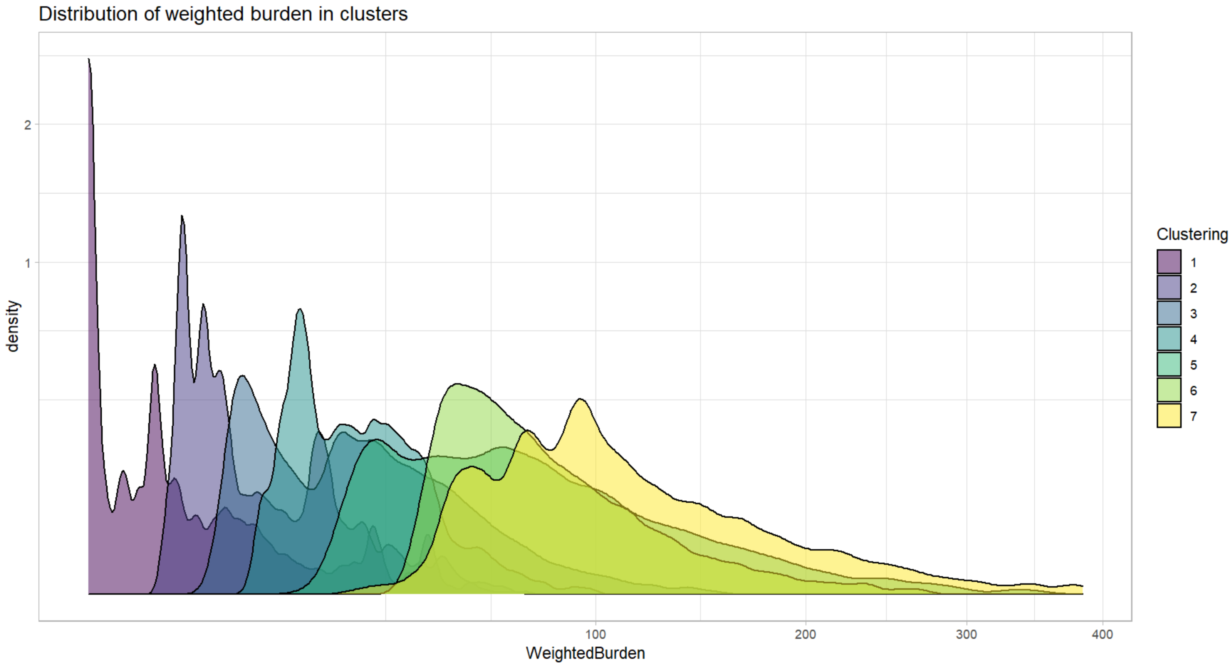

Figure SM5.2. Distribution of weighted direct MDD-related multimorbidity burden in the clusters where the number of clusters is set to 7 in the discovery cohorts (UKB, N = 502,504; CHSS, N = 645,913; THL, N = 41,092). See the caption of Figure SM3.2 for details.

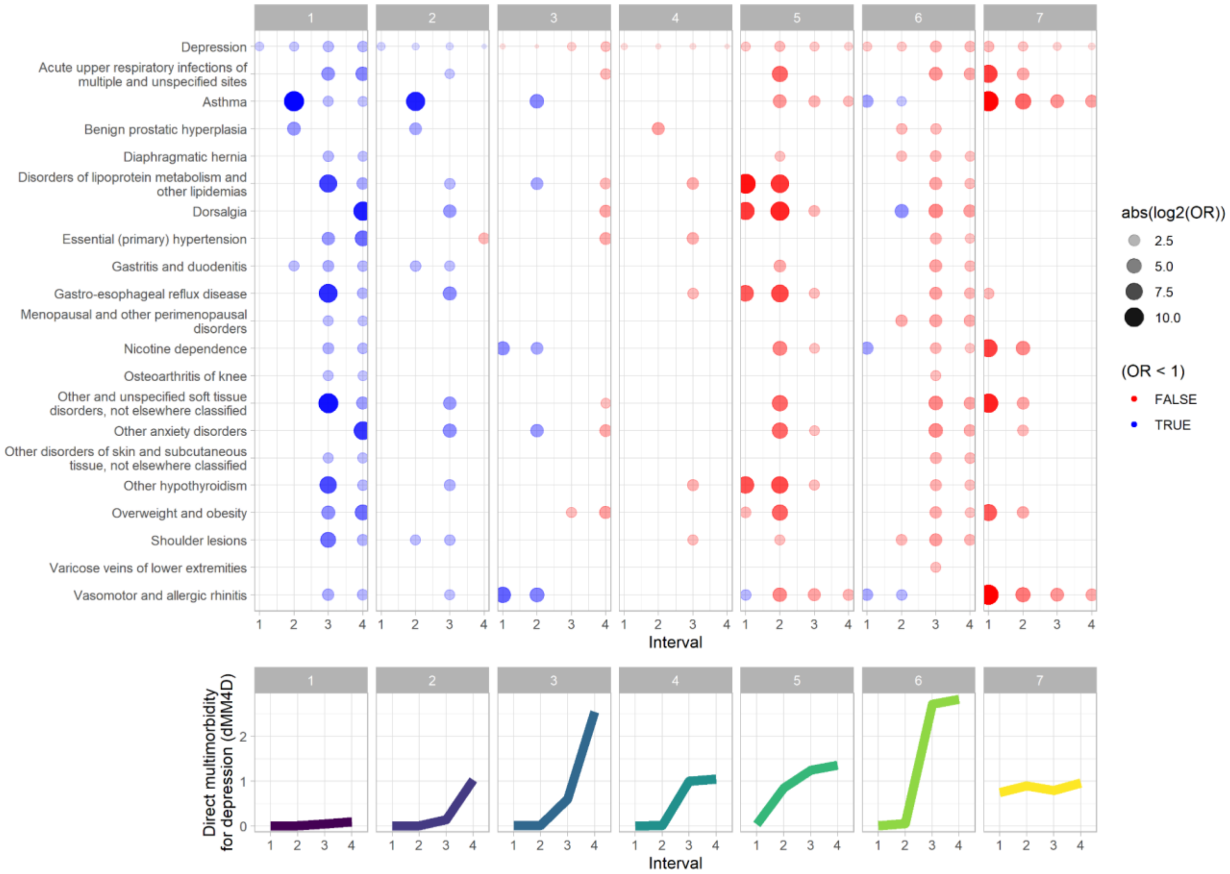

Figure SM5.3. The top over- and underrepresented diseases in each discrete time interval in the clusters where the number of clusters is set to 7 in the discovery cohorts (UKB, N = 502,504; CHSS, N = 645,913; THL, N = 41,092). See the caption of Figure SM3.3 for details.

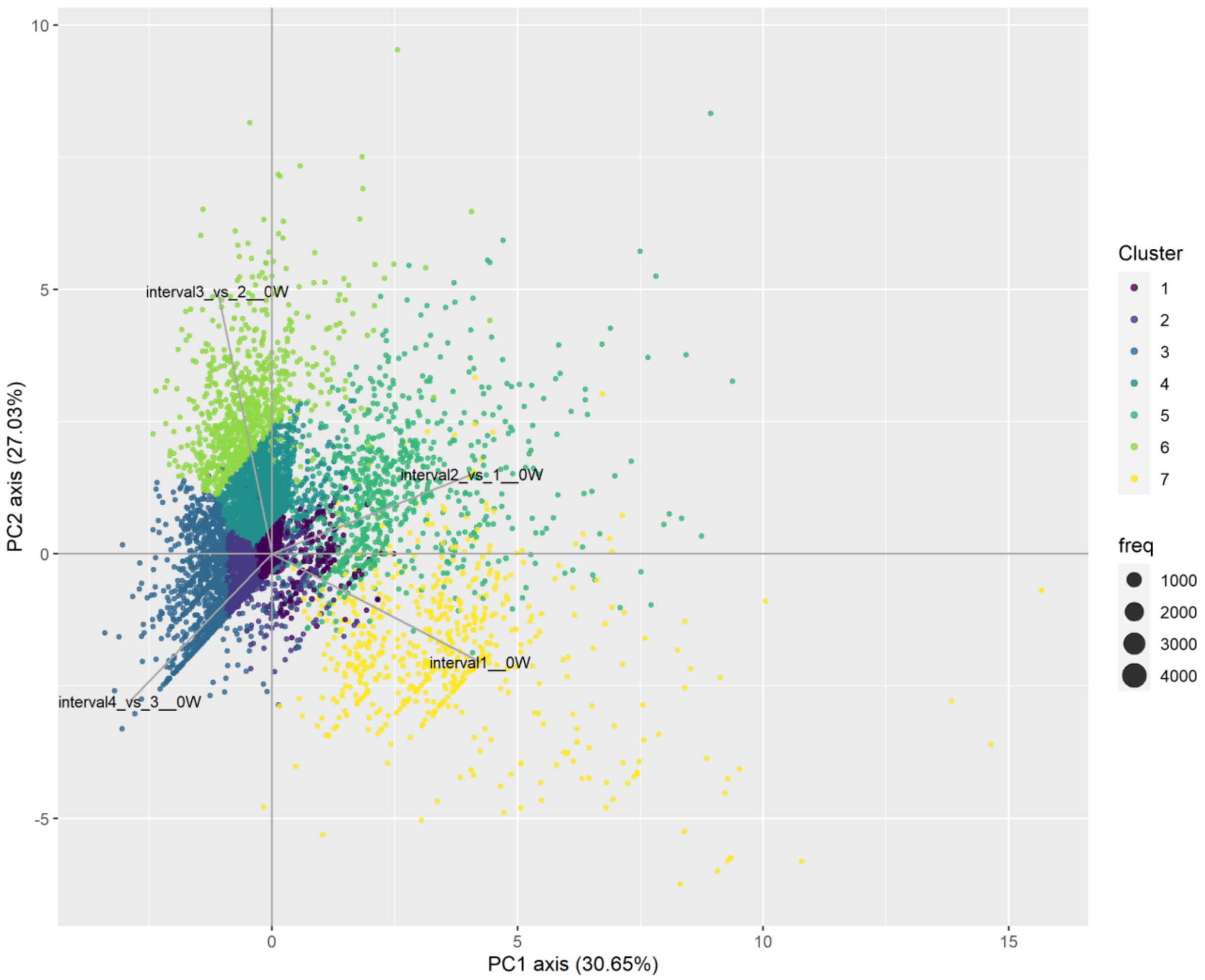

Figure SM5.4. The first two principal components of the 4-dimensional space defined by the temporal MDD-related multimorbidity burden score of the participants where the number of clusters is set to 7 in the discovery cohorts (UKB, N = 502,504; CHSS, N = 645,913; THL, N = 41,092). See the caption of Figure SM3.4 for details.

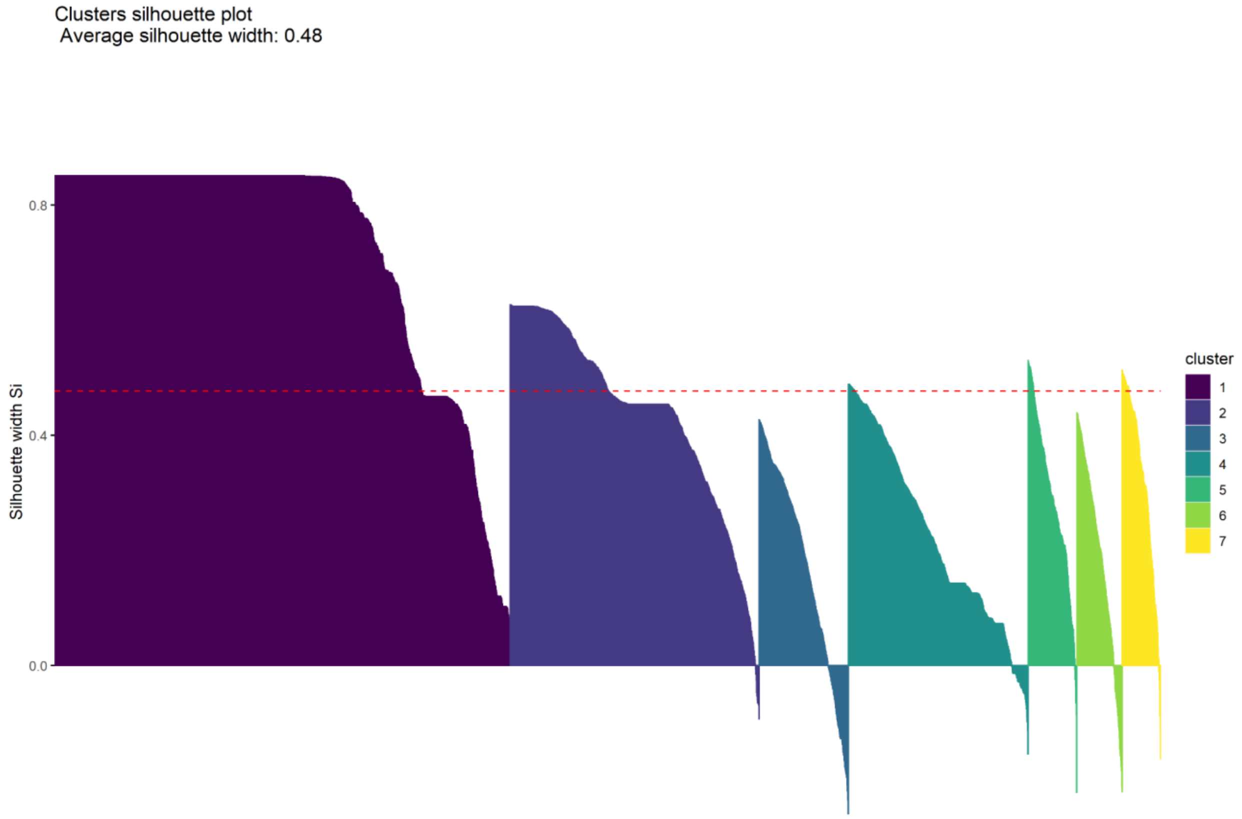

Figure SM5.5. Silhouette plot of the clusters where the number of clusters is set to 7 in the discovery cohorts (UKB, N = 502,504; CHSS, N = 645,913; THL, N = 41,092). See the caption of Figure SM3.5 for details.

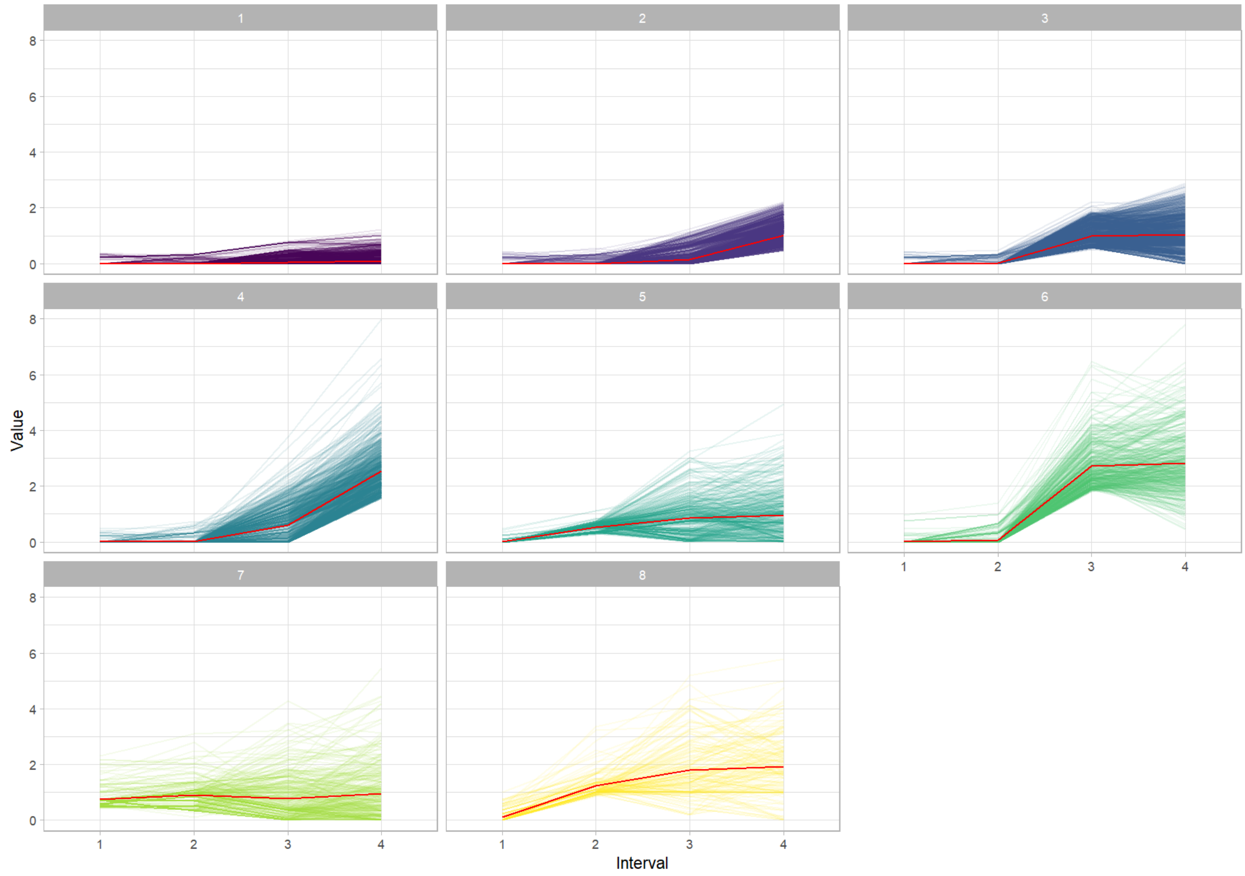

Figure SM6.1. Trajectories of the weighted direct MDD-related multimorbidity score over time using 8 clusters

in the discovery cohorts (UKB, N = 502,504; CHSS, N = 645,913; THL, N = 41,092). See the caption of Figure SM3.1 for details.

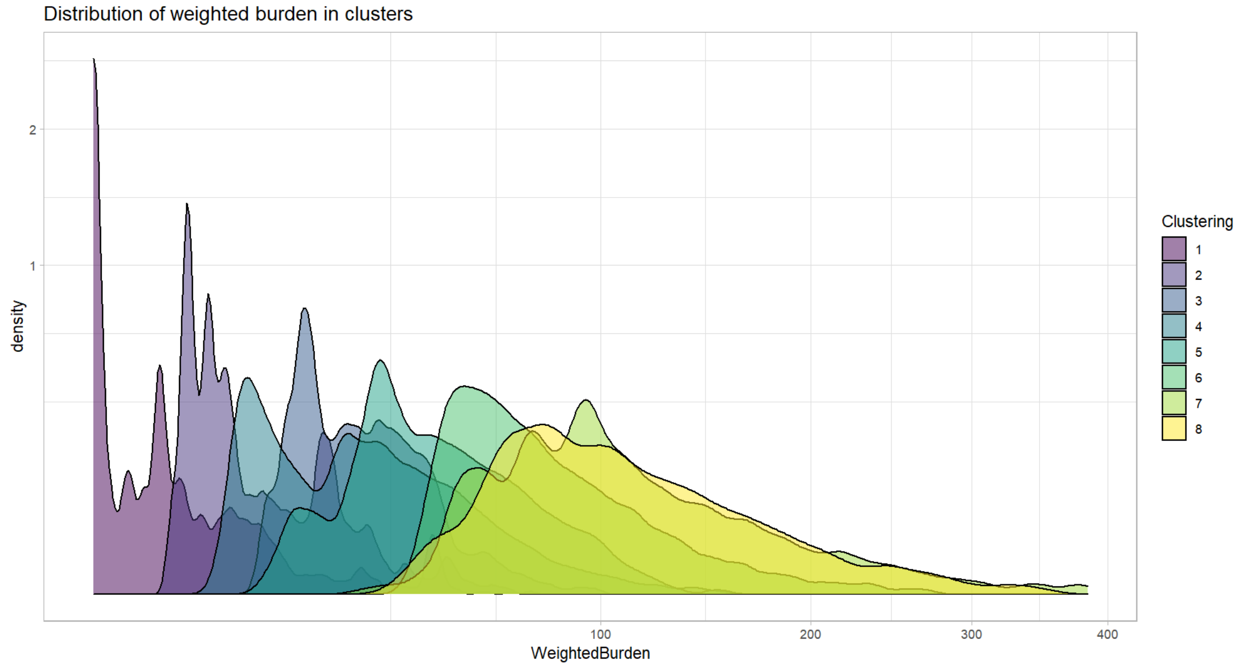

Figure SM6.2. Distribution of weighted direct MDD-related multimorbidity burden in the clusters where the number of clusters is set to 8 in the discovery cohorts (UKB, N = 502,504; CHSS, N = 645,913; THL, N = 41,092). See the caption of Figure SM3.2 for details.

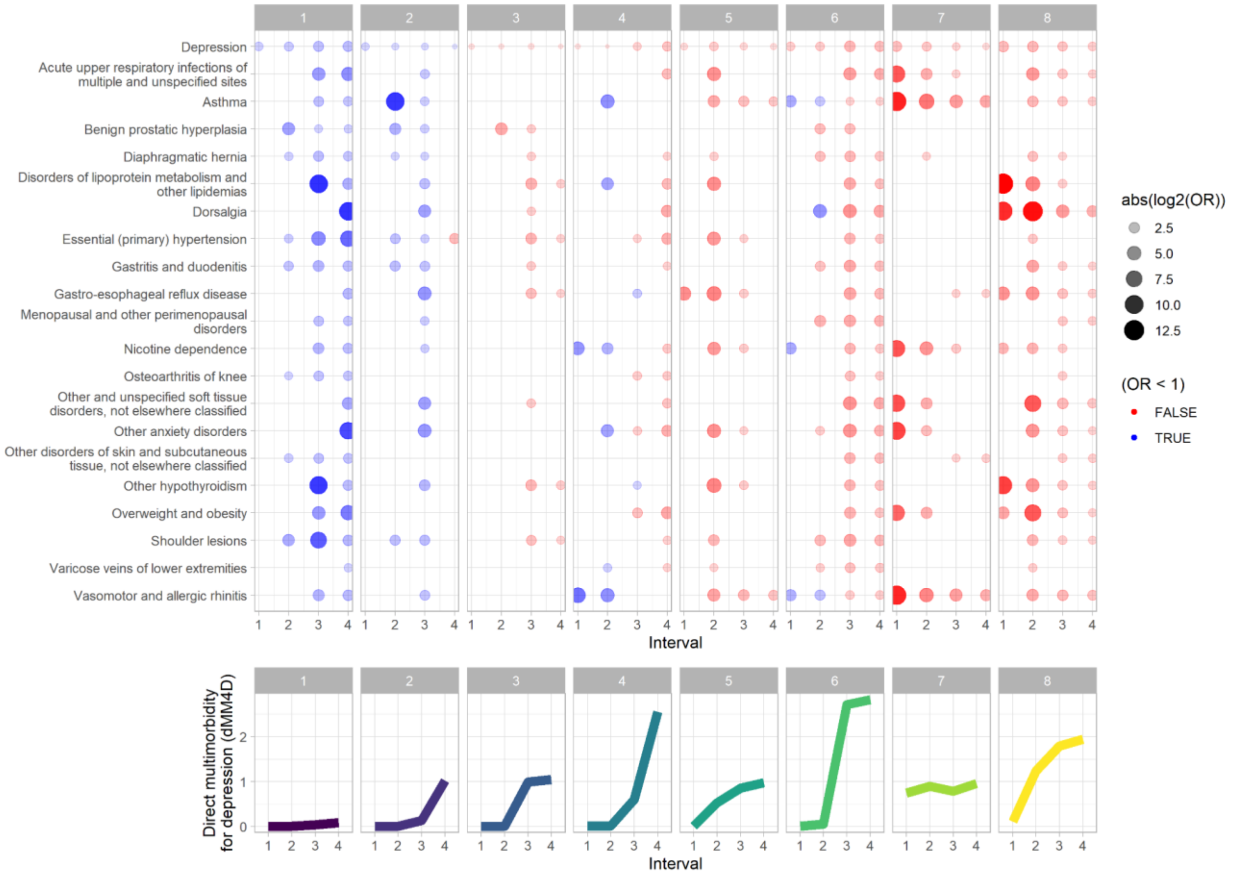

Figure SM6.3. The top over- and underrepresented diseases in each discrete time interval in the clusters where the number of clusters is set to 8 in the discovery cohorts (UKB, N = 502,504; CHSS, N = 645,913; THL, N = 41,092). See the caption of Figure SM3.3 for details.

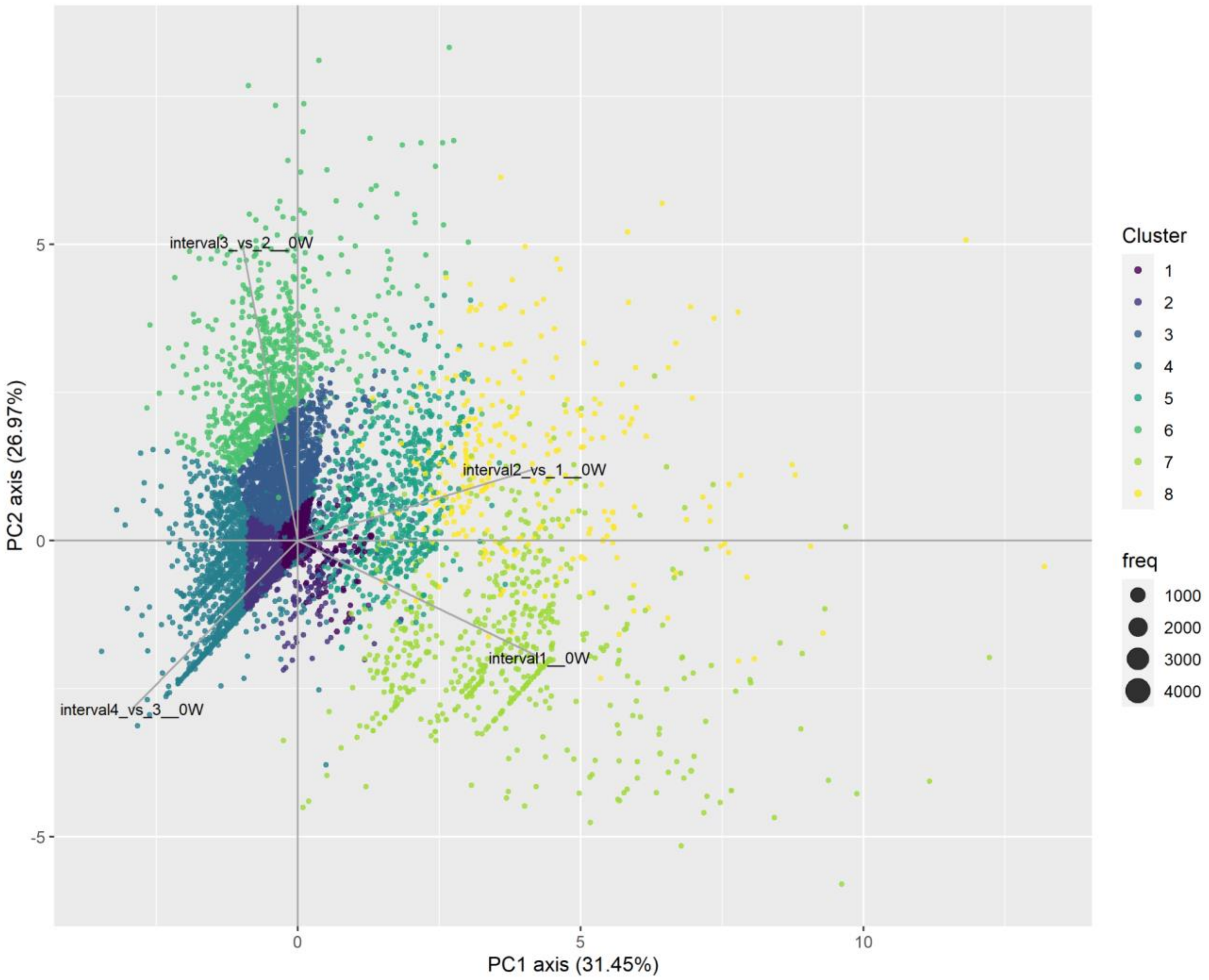

Figure SM6.4. The first two principal components of the 4-dimensional space defined by the temporal MDD-related multimorbidity burden score of the participants where the number of clusters is set to 8 in the discovery cohorts (UKB, N = 502,504; CHSS, N = 645,913; THL, N = 41,092). See the caption of Figure SM3.4 for details.

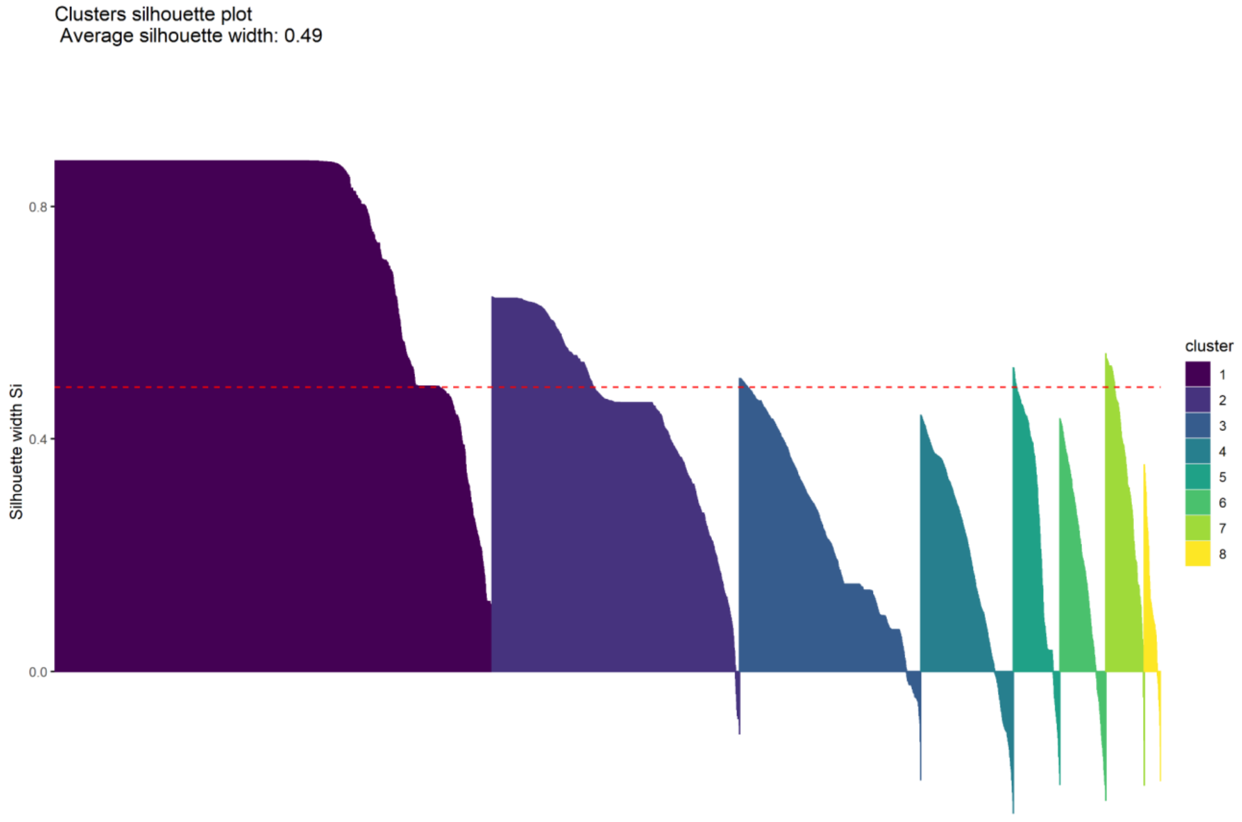

Figure SM6.5. Silhouette plot of the clusters where the number of clusters is set to 8 in the discovery cohorts (UKB, N = 502,504; CHSS, N = 645,913; THL, N = 41,092). See the caption of Figure SM3.5 for details.

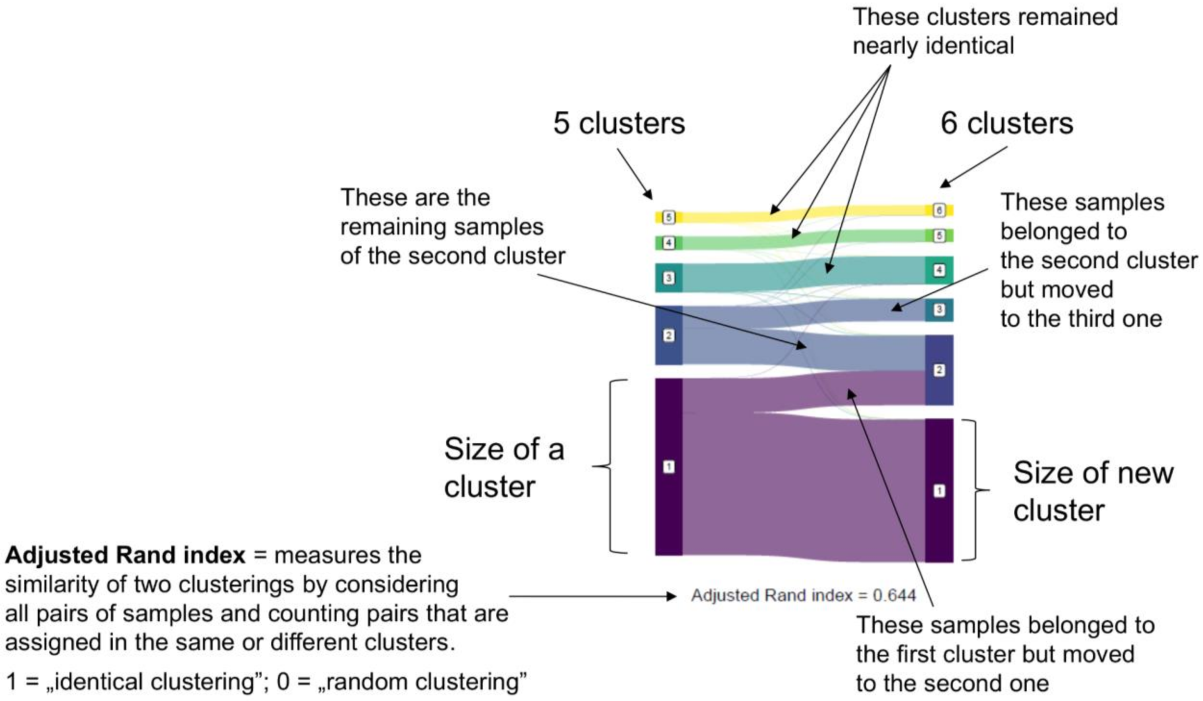

Figure SM7.1. Explanation of the Sankey plots visualizing the difference between the increasing cluster numbers. See Figure SM7.2 for the differences between 5-6, 6-7, 7-8 and 8-9 cluster numbers.

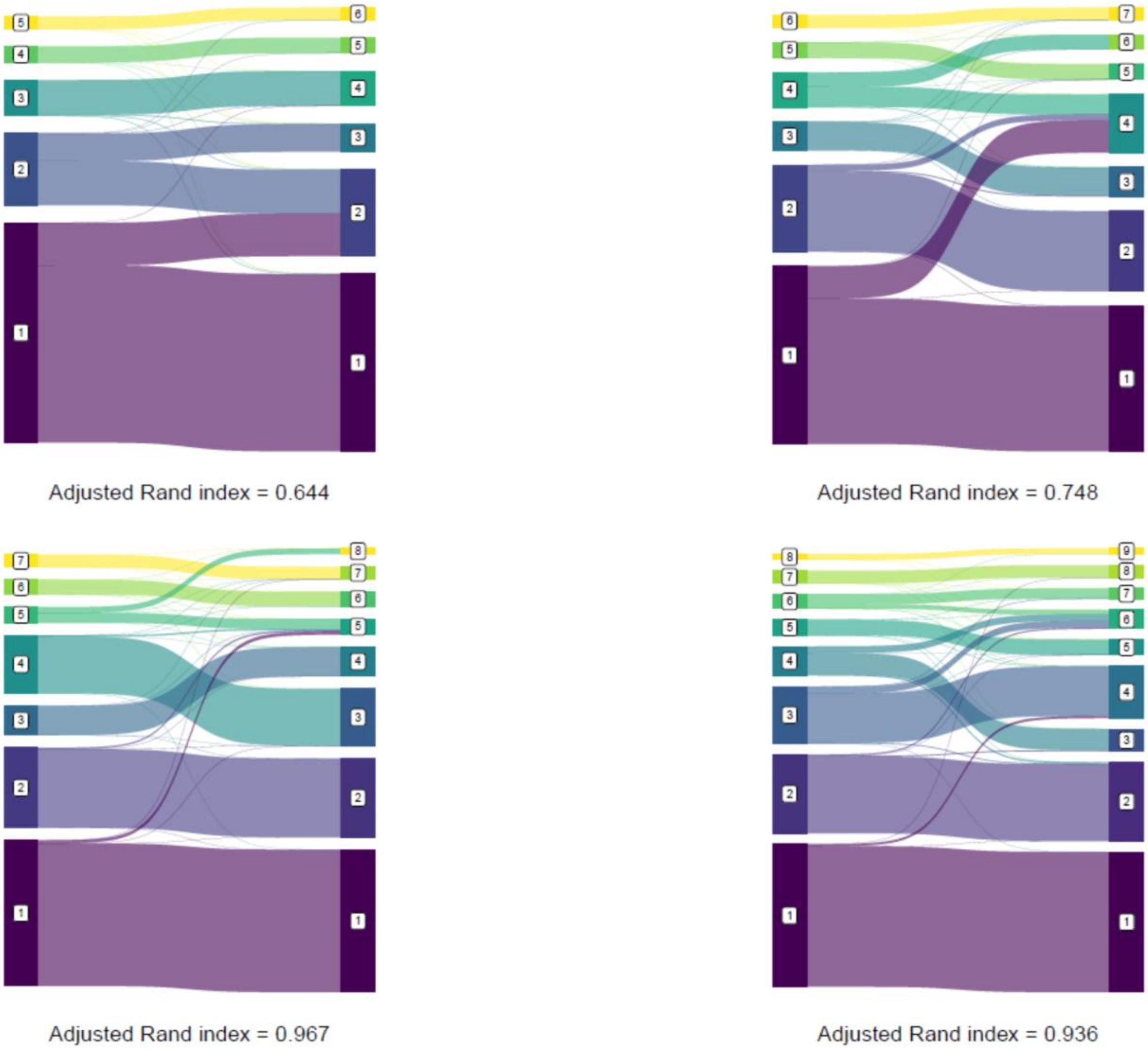

Figure SM7.2. The observed difference between the clusters by increasing the number of clusters in the discovery cohorts (UKB, N = 502,504; CHSS, N = 645,913; THL, N = 41,092). Top left: Increasing the number of clusters from 5 to 6. Top right: Increasing the number of clusters from 6 to 7. Bottom left: Increasing the number of clusters from 7 to 8. Bottom right: Increasing the number of clusters from 8 to 9.

Transferability of clusters in light of different depression phenotypes in UKB

To assess the transferability of clusters between distinct definitions of depression in UKB, we compared results for the 4-item current depressive symptoms score (Supplementary Table S1A) to those for ICD-10 MDD diagnosis, PHQ-9 current depression score, CIDI-based current depression, and CIDI-based lifetime depression.

ICD-10 MDD diagnosis means the lifetime occurrence of either F32 (depressive episode) or F33 (recurrent depressive disorder). Considering the subsample who were included in the genetic analysis, ICD-10 MDD diagnoses were available for 220,315 controls and 28,851 MDD cases.

PHQ-9 score (Davis et al, 2020) was calculated as a Likert scale: the sum of items ‘20514-0.0’, ‘20510-0.0’, ‘20517-0.0’, ‘20519-0.0’, ‘20511-0.0’, ‘20507-0.0’, ‘20508-0.0’, ‘20518-0.0’, and ‘20513-0.0’, with values of 1: Not at all, 2: Several days, 3: More than half the days, 4: Nearly every day. The sum was divided by the number of responded items and multiplied by 9 (total item number), yielding a scale of 9-36.

For the CIDI-depression diagnosis, we followed the work of Cai et al, 2020. This definition is based on the DSM-5, and involves the following criteria: one of the two cardinal symptoms of depression must be present; the total number of depressive symptoms should reach 5; it should have a severe impact on carrying out everyday roles (data-field 20440=3); and another disorder should not better explain these symptoms (we excluded participants with substance abuse disorders, schizophrenia, manic and bipolar disorders). In case of questionnaire items concerning the whole lifetime (CIDI-based lifetime depression), the coding of the symptoms was binary; for the items concerning the last 2 weeks (PHQ-9), the response fell between 1-4, and in this case, only the value of 4 was considered as the presence of the respective symptom (CIDI-based current depression).

As we did not find guidelines for the handling of the missing values for the unanswered questions, we took a data-conserving approach, and retained all the incomplete rows where the MDD-status could be determined with 100% certainty, e.g. we retained participants where the criteria for MDD was already met based on the present data, even though some questions were left unanswered (but they would not change the outcome). Note that this approach skews the distribution, as it is easier to determine the certain lack of MDD than the certain presence.

PHQ-9 score is available for an n = 157,321 in the complete UKB. To align with our other analyses, we filtered for those who were also included in the genetic analysis. In the resulting sub-sample, CIDI-based current depressive episode has 348 cases and 78,453 controls, while CIDI-based lifetime depression phenotype has 11,349 cases and 66,161 controls. For some participants, the CIDI-based depression status was undeterminable.

Linear regression models were run for cluster membership probability log-odds as the outcome. Predictors in each model were sex and age, in addition to the respective depression phenotype. Standardized regression coefficients of each depression phenotype, with a 95% confidence interval, are displayed in the below figures for each cluster membership.

Our results show a robust negative association of depression with Clusters 1-3, and their order in that also seems irrespective of depression definition (Figure SM8): Cluster 1 < Cluster 2 < Cluster 3, with the first showing the strongest negative association, although confidence intervals are high and do overlap in the case of CIDI-based current depression. Cluster 4 shows no major association with different depression phenotypes. Clusters 5 and 6 show a strong positive association with depression irrespective of its definition, but their order is not robust between different depression definitions (Figure SM8). Particularly, Cluster 5 shows the strongest association with depression according to the CIDI-based current depression, while Cluster 6 shows a stronger association with depression according to the current depression score and ICD-10 MDD; and there is no considerable difference between the two clusters in CIDI-based lifetime depression and PHQ-9 score. Regarding Cluster 7, it seems unrelated to ICD-10 MDD and CIDI-based lifetime depression, but shows a negative association with current depression score, CIDI-based current depression and PHQ-9 depression score (Figure SM8).

In summary, cluster membership showed a consistent association pattern with depression with minor differences according to the types of definitions. Thus, we included the ICD-10 MDD diagnosis (Figure 3 B-C) and current depression score (Figure 5., Supplementary Figure S12.) as the main depression descriptors for further analysis because they have the highest sample size (Supplementary Table S1A and S1B).

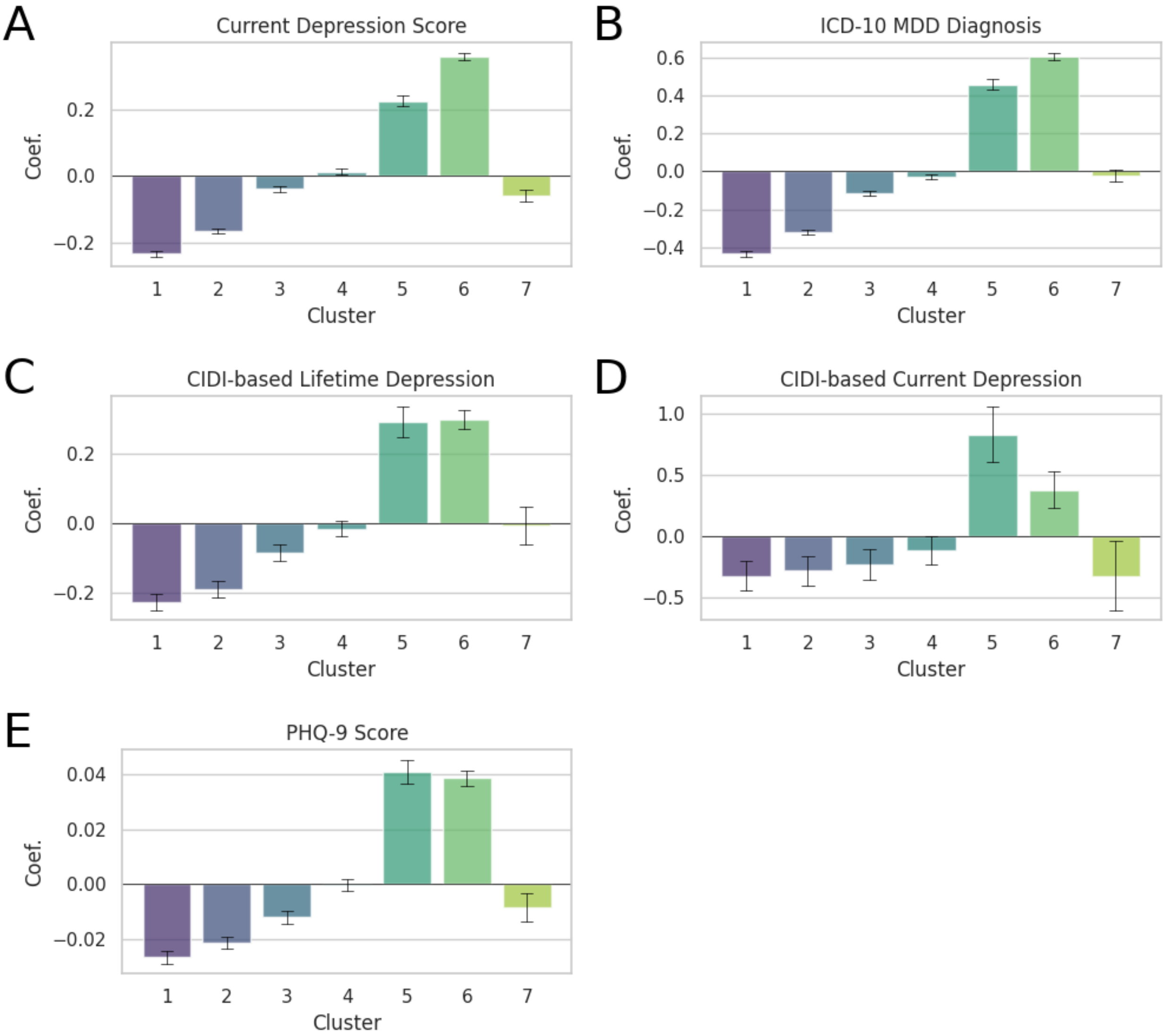

Figure SM8 A-E. Regression coefficient of depression in linear regression models for cluster membership probability log-odds as the target variable. Predictors in each model were sex and age, in addition to the respective depression phenotype. Standardized regression coefficients of each depression phenotype, with a 95% confidence interval, are displayed for each cluster membership.

References

1.) Lautner-Csorba O, Gézsi A, Semsei ÁF, et al. (2012) Candidate gene association study in pediatric acute lymphoblastic leukemia evaluated by Bayesian network based Bayesian multilevel analysis of relevance. BMC Med Genomics 5, 42. https://doi.org/10.1186/1755-8794-5-42
2.) Ungvári I, Hullám G, Antal P, et al. (2012) Evaluation of a Partial Genome Screening of Two Asthma Susceptibility Regions Using Bayesian Network Based Bayesian Multilevel Analysis of Relevance. PLoS ONE 7(3): e33573. https://doi.org/10.1371/journal.pone.0033573
3.) Lautner-Csorba O, Gézsi A, Erdélyi DJ, Hullám G, Antal P, et al. (2013) Roles of Genetic Polymorphisms in the Folate Pathway in Childhood Acute Lymphoblastic Leukemia Evaluated by Bayesian Relevance and Effect Size Analysis. PLoS ONE 8(8): e69843. https://doi.org/10.1371/journal.pone.0069843
4.) Antal P, Millinghoffer A, Hullám G, et al. (2014) Bayesian, systems-based, multilevel analysis of associations for complex phenotypes: from interpretation to decisions. Probabilistic Graphical Models for Genetics, Genomics and Postgenomics. Oxford University Press: Oxford, UK.
5.) Marx P, Antal P, Bolgar B, Bagdy G, Deakin B, Juhasz G (2017) Comorbidities in the diseasome are more apparent than real: What Bayesian filtering reveals about the comorbidities of depression. PLoS Comput Biol 13(6): e1005487. https://doi.org/10.1371/journal.pcbi.1005487
6.) Davis KAS, Coleman JRI, Adams M, et al. (2020) Mental health in UK Biobank – development, implementation and results from an online questionnaire completed by 157 366 participants: a reanalysis. BJPsych Open 6(e18): 1-8. https://doi.org/10.1192/bjo.2019.100
7.) Cai N, Revez JA, Adams MJ, et al. (2020) Minimal phenotyping yields genome-wide association signals of low specificity for major depression. Nat Genet 52(4): 437-447. https://doi.org/10.1038/s41588-020-0594-5
