# Supplementary material for: Unique genetic and risk-factor profiles in clusters of major depressive disorder-related multimorbidity trajectories
Source: Nat Commun. 2024 Aug 21;15:7190. doi: 10.1038/s41467-024-51467-7 (PMC11339304; doi:10.1038/s41467-024-51467-7)
Supplement: Supplementary file 3 — Description of Additional Supplementary Files [file 41467_2024_51467_MOESM3_ESM.pdf]

Description of Additional Supplementary Files

Supplementary Data 1A-B: Extended cohort description.

Supplementary Data 2: Descriptive statistics of onset and prevalence of cross-cohort diseases in the entire cohorts and in the MDD subpopulation of the cohorts.

Supplementary Data 3: Consensual relevance scores of the cross-cohort diseases.

Supplementary Data 4-10: Significant GWAS loci for MDD-related clusters in UKB (N = 249,167). The GWAS summary statistics for all seven MDD-related clusters for each cohort were first processed with FUMA to identify lead SNPs and significant loci. The maximum p-value of lead SNPs was set to 5×10^{-8}, $r^2 \geq 0.6$ was set as the threshold for independent significant SNPs, and the maximum distance between LD blocks of independent significant SNPs was set to 250 kb.

Supplementary Data 11-18: MAGMA gene-based results from GWAS for MDD-related clusters in UKB (N = 249,167). MAGMA gene-level analysis was performed to identify putative significant genes using a SNPwise-multi model. We defined the SNP set of each gene including ± 10 kb downstream or upstream of the gene, respectively. We used the 1000 Genomes European panel data to evaluate the LD between SNPs. We employed Holm's correction method to adjust the p-values of the genes.

Supplementary Data 19: Gene-set based functional enrichment results for MDD-related clusters in UKB (N = 249,167). The g:Profiler R package was used for functional enrichment analysis of each cluster's sets of significant genes. We used Gene Ontology (excluding IEA evidence codes) and KEGG biological pathway data sources. We applied the g:SCS method for p-value adjustment, and the $p<0.01$ threshold was used to indicate statistical significance.

Supplementary Data 20: Variance explained by the PRS based on UKB results in the SHIP (N=1108) and THL (N=30,961) cohorts. PRS was calculated using PRS-CS. The original effect sizes were taken from the UKB GWAS on cluster membership for all seven clusters. The LD reference panel was constructed using a European subsample of the UK Biobank. For the remaining parameters, the default options implemented in PRS-CS were adopted. The PRSs for membership in Clusters 1-7 were calculated in the GWAS samples of the THL and SHIP cohorts. PRSs in the SHIP cohort were correlated with the cluster probabilities, whereas in the THL cohorts, due to the larger sample size, regression analyses between two factors could be performed adjusted for age, sex, batch, region and cohort.

Supplementary Data 21A-B: Replicated loci and genes from FinnGen GWAS (N = 277,252, p<0.05). The FinnGen GWAS analyses were performed with Regenie (v2.2.4). We applied linear regression models to assess the direct effect of each SNP on the seven MDD-related clusters that reflected the posterior log-odds of cluster membership. All analyses were adjusted for age, sex, and the first ten genetic principal components.

Supplementary Data 22-29: MAGMA gene-based results from GWAS for MDD-related clusters in FinnGen (N = 277,252). MAGMA gene-level analysis was performed to identify putative significant genes using a SNPwise-multi model. We defined the SNP set of each gene including ± 10 kb downstream or

upstream of the gene, respectively. We used the 1000 Genomes European panel data to evaluate the LD between SNPs. We employed Holm's correction method to adjust the p-values of the genes.

Supplementary Data 30: Gene-set based functional enrichment results for MDD-related clusters in FinnGen (N = 277,252). The g:Profiler R package was used for functional enrichment analysis of each cluster's sets of significant genes. We used Gene Ontology (excluding IEA evidence codes) and KEGG biological pathway data sources. We applied the g:SCS method for p-value adjustment, and the $p<0.01$ threshold was used to indicate statistical significance.

Supplementary Data 31: Genetic correlation between cohorts (UKB, N = 249,167; FinnGen, N = 277,252; THL, N = 30,961). Genetic correlations between the clusters were computed using the LD score regression method.
