# Supplementary material for: Unique genetic and risk-factor profiles in clusters of major depressive disorder-related multimorbidity trajectories
Source: Nat Commun. 2024 Aug 21;15:7190. doi: 10.1038/s41467-024-51467-7 (PMC11339304; doi:10.1038/s41467-024-51467-7)
Supplement: Supplementary file 5 — Reporting Summary [file 41467_2024_51467_MOESM5_ESM.pdf]

nature portfolio

Corresponding author(s): Gabriella Juhasz

Last updated by author(s): Jul 24, 2024

Reporting Summary

Nature Portfolio wishes to improve the reproducibility of the work that we publish. This form provides structure for consistency and transparency in reporting. For further information on Nature Portfolio policies, see our Editorial Policies and the Editorial Policy Checklist.

Statistics

For all statistical analyses, confirm that the following items are present in the figure legend, table legend, main text, or Methods section.

n/a	Confirmed	
☐	☒	The exact sample size (*n*) for each experimental group/condition, given as a discrete number and unit of measurement
☐	☒	A statement on whether measurements were taken from distinct samples or whether the same sample was measured repeatedly
☐	☒	The statistical test(s) used AND whether they are one- or two-sided<br>*Only common tests should be described solely by name; describe more complex techniques in the Methods section.*
☐	☒	A description of all covariates tested
☐	☒	A description of any assumptions or corrections, such as tests of normality and adjustment for multiple comparisons
☐	☒	A full description of the statistical parameters including central tendency (e.g. means) or other basic estimates (e.g. regression coefficient) AND variation (e.g. standard deviation) or associated estimates of uncertainty (e.g. confidence intervals)
☐	☒	For null hypothesis testing, the test statistic (e.g. *F*, *t*, *r*) with confidence intervals, effect sizes, degrees of freedom and *P* value noted<br>*Give P values as exact values whenever suitable.*
☐	☒	For Bayesian analysis, information on the choice of priors and Markov chain Monte Carlo settings
☐	☒	For hierarchical and complex designs, identification of the appropriate level for tests and full reporting of outcomes
☐	☒	Estimates of effect sizes (e.g. Cohen's *d*, Pearson's *r*), indicating how they were calculated

Our web collection on statistics for biologists contains articles on many of the points above.

Software and code

Policy information about availability of computer code

Data collection

No software was used for data collection, we used existing population cohorts and biobank data.

Data analysis

MDD-related multimorbidity posteriors: Inference over Bayesian network structures was performed with an in-house developed software called BN-BMLA (https://doi.org/10.1371/journal.pcbi.1005487). A standalone version of the software is available for access at the following GitHub link: https://github.com/gezsi/mdd-clustering.
MDD-related multimorbidity clusters: Clusters can be computed with a command line R script that is available online: https://github.com/gezsi/mdd-clustering.
All other computations were performed in R statistical software (version 4.1.1) or Python (version 3.8).
Non-genetic risk factor profiles of the clusters were computed using the statsmodels Python package (v0.13.1).
GWAS: Plink 2.0 (UKB, THL, SHIP), Regenie v2.2.4 (FinnGen) was used for genome-wide association study. Furthermore we used several freely available post-GWAS tools (e.g. FUMA, MAGMA (v1.10), g:Profiler (v0.2.3, database version: e110_eg57_p18_4b54a898) R package, fgsea (v1.18) R package, Complex-Traits Genetics Virtual Lab, LDSC (v1.01), PRS-CS (v1.0.0)). For FinnGen QC steps https://finngen.gitbook.io/finngen-analyst-handbook/finngen-data-specifics/genotype-data/imputation-panel/sisu-v4-reference-panel was applied, for Finrisk QC steps https://finngen.gitbook.io/finngen-analyst-handbook/finngen-data-specifics/genotype-data/imputation-panel/sisu-v3-reference-panel was used, and for SHIP imputation Eagle and minimac3 software were implemented in the Michigan Imputation Server.

For manuscripts utilizing custom algorithms or software that are central to the research but not yet described in published literature, software must be made available to editors and reviewers. We strongly encourage code deposition in a community repository (e.g. GitHub). See the Nature Portfolio guidelines for submitting code & software for further information.

Data

Policy information about availability of data

All manuscripts must include a data availability statement. This statement should provide the following information, where applicable:

- Accession codes, unique identifiers, or web links for publicly available datasets
- A description of any restrictions on data availability
- For clinical datasets or third party data, please ensure that the statement adheres to our policy

The following cohorts and biobank data were used for analysis which are available for further research upon application to the data owners: UK Biobank (https://www.ukbiobank.ac.uk/, application number:1602), Catalan Health Surveillance System (CHSS) registry data from all citizens living in the integrated health district of Barcelona-Esquerra ("AISBE") (https://doi.org/10.1186/s12913-019-4174-2), Finnish population surveys (THL, https://thl.fi/en/web/thlfi-en/research-and-development/research-and-projects/previous-research-and-projects), FinnGen project (https://www.finngen.fi/en), and Study of Health in Pomerania (SHIP, https://doi.org/10.1093/ije/dyac034).

Research involving human participants, their data, or biological material

Policy information about studies with human participants or human data. See also policy information about sex, gender (identity/presentation), and sexual orientation and race, ethnicity and racism.

Reporting on sex and gender	In all analyses self-reported sex was covariate. Sex distribution of investigated cohorts can be seen in Table 1. To increase the statistical power, at this stage of our project we have not done sex stratified analysis. Gender information has not been collected and used in this study.<br>For genetic analysis we selected participants without putative sex chromosome aneuploidy and where the genetic sex did not match the participant-reported sex, participants were removed.
Reporting on race, ethnicity, or other socially relevant groupings	To determine MDD-related multimorbidity clusters whole cohort data were used regardless of race, ethnicity, or other socially relevant groupings. In the analysis age-groups ([0-20], [0-40], [0-60], and [0-70]), sex and household income status (self-reported) variables were used beside first onsets of diseases.<br><br>For genetic analysis we selected participants with White European ancestry (defined both by self-report and genetic ancestry) as they were overrepresented in our cohorts and provided the highest statistical power for analysis. All analyses were adjusted for age, sex, the first ten genetic principal components and site-specific variables (genotyping array in the UKB cohort, geographical region in the THL cohorts).
Population characteristics	Basic population charactersitics are summarised in Table 1 and Supplementary Table S1. In short:<br>UK Biobank: We extracted data from the UK Biobank (UKB) database, which includes medical (first onset of diseases using ICD-10 codes), phenotypic (age, sex, education, household income, alcohol intake, smoking, BMI, stress, neuroticism, insomnia, depression score, blood pressure, pulse rate, CRP) and genotypic data of participants recruited from NHS patient registers of people aged 40–69 years.<br>CHSS: Catalan Health Surveillance System (CHSS) has collected detailed information on healthcare utilization from the entire population of Catalonia (northeastern Spain; 7.5 million inhabitants). We considered only registry data from all citizens living in the integrated health district of Barcelona-Esquerra ("AISBE"). They have data on first onset of diseases using ICD-10 codes, age, sex, and household income but do not have genotypic data.<br>THL: Participants from the Finnish population surveys were analysed, including medical (first onset of diseases using ICD-10 codes), phenotypic (age, sex, education, household income, alcohol intake, smoking, BMI, insomnia, depression score, blood pressure, pulse rate, CRP) and genotypic data of participants.<br>FinnGen: FinnGen is a public–private project aiming to collect genotype data from half a million Finnish people and combine these data with data from various health registries. This cohort has medical (first onset of diseases using ICD-10 codes), phenotypic (age, sex,) and genotypic data of participants.<br>SHIP: This is a general population-based research project on adult residents in northeastern Germany, who provided data on age, sex, household income, education, life events and mental disorders, have a limited set of information on first onset of diseases using ICD-10 codes (37 diseses) and genotypic data.
Recruitment	Our cohorts represent different recruitment strategies, see below. The observed differences between cohorts included age range, birth year, and socioeconomic factors, which may have influenced the availability of medical care and disease diagnosis and affected the prevalence rates of lifetime MDD diagnosis (7–26%) in the cohorts.<br><br>UK Biobank: Participants were recruited from NHS patient registers of people aged 40–69 years on a voluntary basis. Individuals who are willing to participate in medical research may be overrepresented.<br>CHSS: Registry data from all citizens living in the integrated health district of Barcelona-Esquerra ("AISBE") was used. Approximately 50% of the records were from the period after 2012, when the Catalan health system underwent digitization and implemented electronic medical records and less data available from the previous time periods.<br>THL: Participants from the Finnish population surveys were involved. These participants, aged 20–100 years, were chosen at random from the Finnish population and represented different parts of Finland.<br>FinGen: The participants consist of legacy subjects recruited before the start of the FinnGen project and prospective subjects; these latter subjects were recruited on a voluntary basis during hospital visits if the patient provided consent for their data to be entered in the biobank.<br>SHIP: This general population-based research project recruited adult residents in northeastern Germany with White

	European ancestry between 1997 and 2001.
Ethics oversight	UK Biobank Ethics statement: Under the application number 1602 we used data from the UK Biobank (UKB) database resource which includes medical and phenotypic data of recruited participants on the NHS patient registers of people aged 40–69 years (DOI: 10.1371/journal.pone.0075362). Ethical approval was given by the National Research Ethics Service Committee North West–Haydock (11/NW/0382, 21/NW/0157), and all participants gave written informed consent. All procedures were in accordance with the Declaration of Helsinki.<br><br>Finngen/THL DF 10 Ethics statement: Patients and control subjects in FinnGen provided informed consent for biobank research, based on the Finnish Biobank Act. Alternatively, separate research cohorts, collected prior the Finnish Biobank Act came into effect (in September 2013) and start of FinnGen (August 2017), were collected based on study-specific consents and later transferred to the Finnish biobanks after approval by Fimea (Finnish Medicines Agency), the National Supervisory Authority for Welfare and Health. Recruitment protocols followed the biobank protocols approved by Fimea. The Coordinating Ethics Committee of the Hospital District of Helsinki and Uusimaa (HUS) statement number for the FinnGen study is Nr HUS/990/2017.<br><br>The FinnGen study is approved by Finnish Institute for Health and Welfare (permit numbers: THL/2031/6.02.00/2017, THL/1101/5.05.00/2017, THL/341/6.02.00/2018, THL/2222/6.02.00/2018, THL/283/6.02.00/2019, THL/1721/5.05.00/2019 and THL/1524/5.05.00/2020), Digital and population data service agency (permit numbers: VRK43431/2017-3, VRK/6909/2018-3, VRK/4415/2019-3), the Social Insurance Institution (permit numbers: KELA58/522/2017, KELA 131/522/2018, KELA 70/522/2019, KELA 98/522/2019, KELA 134/522/2019, KELA 138/522/2019, KELA 2/522/2020, KELA 16/522/2020), Findata permit numbers THL/2364/14.02/2020, THL/4055/14.06.00/2020, THL/3433/14.06.00/2020, THL/4432/14.06/2020, THL/5189/14.06/2020, THL/5894/14.06.00/2020, THL/6619/14.06.00/2020, THL/209/14.06.00/2021, THL/688/14.06.00/2021, THL/1284/14.06.00/2021, THL/1965/14.06.00/2021, THL/5546/14.02.00/2020, THL/2658/14.06.00/2021, THL/4235/14.06.00/2021, Statistics Finland (permit numbers: TK-53-1041-17 and TK/143/07.03.00/2020 (earlier TK-53-90-20) TK/1735/07.03.00/2021, TK/3112/07.03.00/2021) and Finnish Registry for Kidney Diseases permission/extract from the meeting minutes on 4thJuly 2019.<br><br>The Biobank Access Decisions for FinnGen samples and data utilized in FinnGen Data Freeze 10 include: THL Biobank BB2017_55, BB2017_111, BB2018_19, BB_2018_34, BB_2018_67, BB2018_71, BB2019_7, BB2019_8, BB2019_26, BB2020_1, BB2021_65, Finnish Red Cross Blood Service Biobank 7.12.2017, Helsinki Biobank HUS/359/2017, HUS/248/2020, HUS/150/2022 § 12, §13, §14, §15, §16, §17, §18, and §23, Auria Biobank AB17-5154 and amendment #1 (August 17 2020) and amendments BB_2021-0140, BB_2021-0156 (August 26 2021, Feb 2 2022), BB_2021-0169, BB_2021-0179, BB_2021-0161, AB20-5926 and amendment #1 (April 23 2020)and it´s modification (Sep 22 2021), Biobank Borealis of Northern Finland_2017_1013, 2021_5010, 2021_5018, 2021_5015, 2021_5023, 2021_5017, 2022_6001, Biobank of Eastern Finland 1186/2018 and amendment 22 § /2020, 53§/2021, 13§/2022, 14§/2022, 15§/2022, Finnish Clinical Biobank Tampere MH0004 and amendments (21.02.2020 & 06.10.2020), §8/2021, §9/2022, §10/2022, §12/2022, §20/2022, §21/2022, §22/2022, §23/2022, Central Finland Biobank 1-2017, and Terveystalo Biobank STB 2018001 and amendment 25th Aug 2020, Finnish Hematological Registry and Clinical Biobank decision 18th June 2021, Arctic biobank P0844: ARC_2021_1001.<br><br>CHSS Ethics statement: The Ethics Committee for Human Research at Hospital Clinic de Barcelona approved the study protocol on the 24th of march of 2021 (HCB/2020/1051) in the context of the EU project: ERAPERMED2019-108 - TRAJECTOME. All the data were handled in compliance with the General Data Protection Regulation 2016/679 on data protection and privacy for all individuals within the European Union. The study was conducted in conformity with the Helsinki Declaration (Stronghold Version, Brazil, October 2013) and in accordance with the protocol and the relevant legal requirements (Biomedical Research Act 14/2007 of 3 July).<br><br>SHIP Ethics statement: The study followed the recommendations of the Declaration of Helsinki. The medical ethics committee of the University of Greifswald approved the study protocol, and oral and written informed consents were obtained from each of the study participants.

Note that full information on the approval of the study protocol must also be provided in the manuscript.

Field-specific reporting

Please select the one below that is the best fit for your research. If you are not sure, read the appropriate sections before making your selection.

☒ Life sciences ☐ Behavioural & social sciences ☐ Ecological, evolutionary & environmental sciences

For a reference copy of the document with all sections, see nature.com/documents/nr-reporting-summary-flat.pdf

Life sciences study design

All studies must disclose on these points even when the disclosure is negative.

Sample size	No sample size calculation was performed. Our consortium uses the largest available cohorts and biobank data that contains relevant variables that allow us to test our hypothesis both in discovery phase and in validation phase. Our replicated findings support that these sample sizes were sufficient.
Data exclusions	To determine MDD-related multimorbidity clusters there were no exclusion criteria, all participants with available necessary data from the discovery samples and then from the validation samples were used in the analysis. To control for uncertain participant trajectories in the subsequent analyses, participants were excluded if they were both under 60 years and had a maximum posterior probability <0.25 for any of

the clusters.
For the GWAS analyses, we used standard quality control steps to exclude participants. We included participants with White European ancestry (defined both by self-report and genetic ancestry) as they were overrepresented in our cohorts and provided the highest statistical power for analysis.

Replication

Primary analyses were all performed in the UKB cohort, as this was the largest cohort with all information types available. To identify distinct MDD-related multimorbidity-based clusters and assess their biological profiles, we used individual disease onset information from large cohorts divided into discovery (UKB, CHSS, THL) and validation cohorts (FinnGen, SHIP).
Regarding the effects of MDD, the same disease burden pattern in terms of onset age and prevalence was observed in all five cohorts. This pattern was also reflected in the correlations of cluster membership probabilities throughout all cohorts.
Validation of genetic findings was performed in the Finnish cohorts (FinnGen and THL cohorts), and the overall pattern of correlations among the clusters was replicated in GWAS analyses. Using the data from the FinnGen cohort, which had a sample size comparable to that of the UKB cohort, a large proportion of genetic findings were replicated at the levels of SNPs, genes, and functional enrichment.
The reliability of the modifiable behavioural risk factors was validated in the THL cohorts. Although overall effects were weaker due to the smaller sample size, the pattern was similar to that in the UKB cohort.
Furthermore, in the SHIP cohort we demonstrated that the MDD-related multimorbidity clusters can be applied to settings with limited disease information, which further supports the generalizability of our approach.

Randomization

We have not used randomisation for our study. We used all the participants with available necessary data from the cohorts in the analysis to determine MDD-related multimorbidity clusters. The genotypic and phenotypic (e.g. stress, neuroticism, smoking, BMI) profiles of these clusters were analysed using the posterior log-odds of the cluster memberships as target variables.

Blinding

We have not used any intervention in our study thus blinding was not applied.

Reporting for specific materials, systems and methods

We require information from authors about some types of materials, experimental systems and methods used in many studies. Here, indicate whether each material, system or method listed is relevant to your study. If you are not sure if a list item applies to your research, read the appropriate section before selecting a response.

Materials & experimental systems

n/a	Involved in the study	
☒	☐	Antibodies
☒	☐	Eukaryotic cell lines
☒	☐	Palaeontology and archaeology
☒	☐	Animals and other organisms
☒	☐	Clinical data
☒	☐	Dual use research of concern
☒	☐	Plants

Methods

n/a	Involved in the study	
☒	☐	ChIP-seq
☒	☐	Flow cytometry
☒	☐	MRI-based neuroimaging
